# Supplementary material for: Rotavirus Seasonality: An Application of Singular Spectrum Analysis and Polyharmonic Modeling
Source: Int J Environ Res Public Health. 2019 Nov 6;16(22):4309. doi: 10.3390/ijerph16224309 (PMC6888479; doi:10.3390/ijerph16224309)
Supplement: Supplementary file 1 [file ijerph-16-04309-s001.pdf]

Table S1. The daily rates of rotaviral infection and daily average values of temperature in °C for three Russian cities from 1 January 2005 to 31 December 2011.

Data	Chelyabinsk		Yekaterinburg		Barnaul	
	RI rates	Temperature	RI rates	Temperature	RI rates	Temperature
01.01.2005	0.913159	-13.3	3.066779	-12.1		
02.01.2005	3.652654	-6.6	1.533376	-5.8		
03.01.2005	6.392177	-8.2	4.600089	-5.5		
04.01.2005	3.652692	-15.2	3.833375	-9.9		
05.01.2005	0.913178	-13.5	2.300005	-12.6		
06.01.2005	2.739548	-12.3	1.533324	-12.3		
07.01.2005	2.739562	-17.4	3.066621	-19.3		
08.01.2005	2.739577	-13.8	2.299946	-16.1		
09.01.2005	1.826394	-8.1	0.766642	-12.5		
10.01.2005	4.566009	0.2	2.299906	-0.9		
11.01.2005	0.000000	-5.6	0.000000	-2.8		
12.01.2005	2.739634	-6.3	4.599733	-4.8		
13.01.2005	0.913216	-10.5	3.066462	-11.7		
14.01.2005	1.826442	-17.2	1.533218	-8.8		
15.01.2005	3.652903	-13.9	3.066409	-3.8		
16.01.2005	1.826461	-9.7	1.533191	-4.6		
17.01.2005	0.913235	-5.4	3.066357	-7.7		
18.01.2005	0.000000	-12.7	5.366078	-11.4		
19.01.2005	5.479470	-14.5	3.832880	-12.4		
20.01.2005	2.739749	-7.5	3.832847	-7.1		
21.01.2005	0.913255	-11.2	0.766563	-11.1		
22.01.2005	1.826519	-18.5	3.832781	-15.8		
23.01.2005	1.826528	-19.6	6.132396	-17.0		
24.01.2005	5.479614	-20.1	7.665429	-17.3		
25.01.2005	1.826548	-28.3	1.533073	-26.9		
26.01.2005	0.913279	-22.6	4.599178	-22.6		
27.01.2005	1.826567	-22.2	3.066093	-21.2		
28.01.2005	0.000000	-20.3	6.132132	-20.7		
29.01.2005	5.479758	-20.3	4.599060	-24.8		
30.01.2005	4.566489	-23.3	3.832517	-23.2		
31.01.2005	4.566513	-20.6	2.299490	-21.1		
01.02.2005	3.653229	-17.2	1.532980	-18.1		
02.02.2005	5.479873	-12.6	3.065934	-18.2		
03.02.2005	1.826634	-14.3	5.365339	-16.5		
04.02.2005	3.653287	-15.8	6.131763	-19.3		
05.02.2005	5.479959	-13.8	3.065855	-14.3		
06.02.2005	3.653325	-9.5	0.766457	-10.4		
07.02.2005	5.480017	-11.2	3.832253	-11.9		

Data	Chelyabinsk		Yekaterinburg		Barnaul	
	RI rates	Temperature	RI rates	Temperature	RI rates	Temperature
08.02.2005	3.653364	-11.9	0.766444	-11.4		
09.02.2005	8.220112	-11.5	5.365061	-11.2		
10.02.2005	1.826701	-14.9	6.897877	-17.3		
11.02.2005	3.653421	-16.9	3.065697	-16.5		
12.02.2005	4.566801	-17.9	6.897758	-18.5		
13.02.2005	3.653460	-23.8	4.598466	-18.4		
14.02.2005	5.480219	-24.2	6.131235	-20.5		
15.02.2005	4.566873	-23.3	6.131182	-21.0		
16.02.2005	3.653517	-15.3	3.065565	-11.5		
17.02.2005	5.480305	-5.4	2.299154	-8.9		
18.02.2005	5.480334	-10.5	3.065512	-10.4		
19.02.2005	1.826788	-14.9	3.831857	-17.3		
20.02.2005	2.740196	-18.2	6.130918	-19.7		
21.02.2005	5.480420	-19.3	4.598149	-19.3		
22.02.2005	3.653633	-22.9	5.364461	-22.6		
23.02.2005	2.740239	-15.9	4.598070	-15.1		
24.02.2005	2.740253	-13.1	5.364369	-12.4		
25.02.2005	9.134226	-10.9	2.298995	-12.5		
26.02.2005	2.740282	-13.2	9.195902	-12.4		
27.02.2005	0.913432	-12.5	5.364230	-11.9		
28.02.2005	5.480622	-9.3	7.663120	-9.3		
01.03.2005	3.653767	-1.7	8.429359	-1.2		
02.03.2005	3.653786	-3.5	4.597793	-5.7		
03.03.2005	6.394160	-11.4	7.662922	-12.4		
04.03.2005	8.221105	-11.1	8.429142	-12.8		
05.03.2005	9.134610	-10.9	9.195348	-11.2		
06.03.2005	3.653863	-10.4	7.662724	-10.3		
07.03.2005	9.134706	-7.6	6.130127	-8.9		
08.03.2005	4.567377	-9.4	6.130074	-6.9		
09.03.2005	9.134802	-9.3	10.727537	-6.4		
10.03.2005	2.740455	-2.6	6.129968	-5.6		
11.03.2005	2.740469	-4.6	9.194873	-5.7		
12.03.2005	5.480967	-8.6	6.129863	-8.2		
13.03.2005	2.740498	-6.8	3.831131	-8.6		
14.03.2005	8.221538	-1.8	6.895977	-1.2		
15.03.2005	3.654036	0.1	9.194557	-0.3		
16.03.2005	2.740541	-3.5	8.428271	-5.1		
17.03.2005	8.221667	-6.0	2.298600	-7.2		
18.03.2005	6.394664	-4.4	7.661933	-7.4		
19.03.2005	0.913528	-4.9	6.895680	-7.0		
20.03.2005	4.567665	0.4	5.363261	-0.3		

Data	Chelyabinsk		Yekaterinburg		Barnaul	
	RI rates	Temperature	RI rates	Temperature	RI rates	Temperature
21.03.2005	6.394764	-6.1	5.363215	-7.6		
22.03.2005	7.308341	-5.6	3.830835	-7.6		
23.03.2005	2.740642	-5.3	6.129283	-7.2		
24.03.2005	1.827104	-5.5	5.363076	-9.1		
25.03.2005	6.394899	-6.9	6.895324	-9.3		
26.03.2005	6.394933	-7.7	4.596843	-7.3		
27.03.2005	8.222099	-3.4	6.129072	-4.9		
28.03.2005	3.654286	-2.5	7.661273	-3.8		
29.03.2005	1.827152	-6.7	9.193449	-6.3		
30.03.2005	3.654324	-8.1	5.362799	-8.6		
31.03.2005	0.000000	-8.3	9.193291	-9.1		
01.04.2005	0.913591	-7.3	7.661010	-6.8		
02.04.2005	1.827191	-3.9	6.128755	-2.1		
03.04.2005	3.654401	-0.8	6.894790	-0.3		
04.04.2005	5.481630	-5.1	9.959056	-6.3		
05.04.2005	4.568049	-7.4	2.298224	-7.4		
06.04.2005	3.654458	1.7	7.660680	1.7		
07.04.2005	5.481716	5.1	7.660614	4.0		
08.04.2005	2.740873	4.3	6.894493	3.0		
09.04.2005	4.568145	5.9	5.362338	5.4		
10.04.2005	3.654535	8.6	6.128333	8.2		
11.04.2005	1.827277	8.3	5.362245	8.1		
12.04.2005	3.654574	5.9	6.128228	7.2		
13.04.2005	7.309186	2.7	6.128175	1.3		
14.04.2005	3.654612	4.3	3.064061	3.4		
15.04.2005	3.654631	3.8	8.426096	5.3		
16.04.2005	1.827325	6.9	13.022036	6.3		
17.04.2005	2.741002	8.6	7.659955	10.2		
18.04.2005	6.395706	9.4	13.021812	8.8		
19.04.2005	4.568385	11.6	3.829912	5.9		
20.04.2005	4.568409	11.4	6.893782	9.8		
21.04.2005	5.482120	12.2	3.829846	10.3		
22.04.2005	6.395840	13.3	7.659626	11.9		
23.04.2005	4.568481	18.6	4.595736	17.4		
24.04.2005	4.568505	19.7	5.361646	17.6		
25.04.2005	5.482235	15.4	2.297828	12.9		
26.04.2005	1.827421	7.9	6.893426	7.3		
27.04.2005	3.654862	7.1	4.595578	6.6		
28.04.2005	1.827441	7.2	3.829615	5.8		
29.04.2005	3.654900	9.9	3.829582	9.0		
30.04.2005	2.741190	7.5	7.659098	6.5		

Data	Chelyabinsk		Yekaterinburg		Barnaul	
	RI rates	Temperature	RI rates	Temperature	RI rates	Temperature
01.05.2005	1.827469	7.7	9.956742	6.7		
02.05.2005	0.913739	9.7	5.361277	8.2		
03.05.2005	1.827489	11.6	3.829450	7.8		
04.05.2005	0.913749	13.3	2.297650	9.9		
05.05.2005	6.396277	14.7	7.658769	7.4		
06.05.2005	0.913759	7.3	3.063481	4.4		
07.05.2005	1.827527	7.7	4.595182	7.6		
08.05.2005	5.482610	10.2	4.595143	10.6		
09.05.2005	1.827546	6.9	6.892655	9.4		
10.05.2005	5.482668	9.7	5.360908	11.3		
11.05.2005	4.568914	14.8	5.360862	14.9		
12.05.2005	2.741363	17.8	1.531662	17.8		
13.05.2005	2.741377	19.8	1.531648	19.0		
14.05.2005	5.482783	20.4	3.829088	19.4		
15.05.2005	0.000000	21.1	2.297433	19.3		
16.05.2005	1.827613	20.8	6.126435	19.8		
17.05.2005	0.913812	21.1	8.423776	20.1		
18.05.2005	1.827633	21.1	4.594747	18.9		
19.05.2005	2.741463	18.7	6.126277	19.1		
20.05.2005	6.396782	20.2	1.531556	19.3		
21.05.2005	6.396815	19.1	1.531543	17.9		
22.05.2005	6.396849	12.5	1.531530	12.2		
23.05.2005	3.655361	14.3	1.531517	13.5		
24.05.2005	4.569226	14.7	2.297255	15.8		
25.05.2005	5.483100	17.2	0.765745	16.2		
26.05.2005	4.569274	20.8	3.062954	19.2		
27.05.2005	0.913860	18.8	2.297196	17.2		
28.05.2005	6.397051	15.1	1.531451	13.3		
29.05.2005	1.827738	12.4	0.765719	11.4		
30.05.2005	3.655496	10.3	3.828561	8.3		
31.05.2005	0.000000	9.6	2.297117	8.6		
01.06.2005	4.569418	10.1	2.297097	8.3		
02.06.2005	1.827777	14.3	3.062770	12.6		
03.06.2005	3.655573	24.6	3.062743	21.8		
04.06.2005	0.913898	23.8	1.531359	22.9		
05.06.2005	4.569514	23.2	0.765673	20.8		
06.06.2005	3.655631	13.1	1.531332	12.8		
07.06.2005	3.655650	12.1	0.000000	12.3		
08.06.2005	0.913917	13.9	2.296959	14.2		
09.06.2005	1.827844	13.8	3.828232	15.7		
10.06.2005	1.827854	16.2	2.296919	14.3		

Data	Chelyabinsk		Yekaterinburg		Barnaul	
	RI rates	Temperature	RI rates	Temperature	RI rates	Temperature
11.06.2005	0.000000	18.0	0.000000	16.2		
12.06.2005	0.913936	19.5	1.531253	17.3		
13.06.2005	1.827883	18.7	1.531240	16.6		
14.06.2005	1.827892	17.4	0.000000	16.2		
15.06.2005	2.741853	19.3	2.296821	18.3		
16.06.2005	0.000000	15.1	0.765600	14.0		
17.06.2005	2.741882	11.5	1.531187	10.4		
18.06.2005	2.741896	13.8	0.000000	12.6		
19.06.2005	1.827940	15.4	0.000000	15.2		
20.06.2005	7.311799	17.4	0.000000	14.8		
21.06.2005	0.000000	20.3	0.000000	17.4		
22.06.2005	1.827969	19.6	0.765561	17.2		
23.06.2005	4.569947	18.3	0.765554	14.4		
24.06.2005	1.827988	15.6	0.000000	14.3		
25.06.2005	5.483994	12.2	0.000000	12.2		
26.06.2005	7.312030	15.8	0.000000	17.0		
27.06.2005	1.828017	19.4	0.765528	20.4		
28.06.2005	4.570067	17.5	0.765521	15.2		
29.06.2005	3.656073	17.4	0.000000	16.3		
30.06.2005	1.828046	18.1	0.765508	16.6		
01.07.2005	4.570139	20.0	0.000000	19.4		
02.07.2005	0.914033	19.7	0.000000	19.3		
03.07.2005	3.656150	18.2	0.000000	17.1		
04.07.2005	1.828084	16.6	0.000000	15.3		
05.07.2005	0.000000	13.9	0.000000	11.8		
06.07.2005	1.828104	11.4	0.000000	11.4		
07.07.2005	4.570283	13.9	0.000000	13.7		
08.07.2005	3.656246	11.9	0.000000	13.1		
09.07.2005	0.000000	13.4	0.000000	12.7		
10.07.2005	5.484426	13.9	0.000000	13.5		
11.07.2005	1.828152	15.1	0.000000	15.3		
12.07.2005	1.828161	13.3	0.000000	12.9		
13.07.2005	0.914085	13.9	0.000000	14.4		
14.07.2005	0.914090	13.3	0.765416	14.5		
15.07.2005	0.000000	18.4	0.000000	19.6		
16.07.2005	6.398699	19.6	0.000000	20.7		
17.07.2005	1.828209	17.6	0.765396	17.6		
18.07.2005	4.570548	18.6	0.000000	18.9		
19.07.2005	3.656457	20.7	0.000000	21.3		
20.07.2005	3.656477	21.2	0.000000	22.4		
21.07.2005	5.484744	21.8	0.000000	22.4		

Data	Chelyabinsk		Yekaterinburg		Barnaul	
	RI rates	Temperature	RI rates	Temperature	RI rates	Temperature
22.07.2005	3.656515	20.9	0.765363	22.3		
23.07.2005	0.000000	20.6	0.765357	21.7		
24.07.2005	0.914138	21.1	0.000000	22.3		
25.07.2005	2.742430	21.2	0.000000	21.2		
26.07.2005	2.742444	21.7	0.000000	23.2		
27.07.2005	0.914153	24.2	0.000000	24.7		
28.07.2005	6.399103	24.4	0.000000	25.2		
29.07.2005	6.399137	21.6	0.000000	21.9		
30.07.2005	3.656669	22.4	1.530621	22.7		
31.07.2005	5.485032	19.7	0.000000	23.3		
01.08.2005	3.656707	19.3	0.000000	19.2		
02.08.2005	1.828363	19.6	0.000000	18.3		
03.08.2005	3.656746	19.3	0.765284	18.9		
04.08.2005	2.742574	21.3	0.000000	21.9		
05.08.2005	1.828392	19.3	0.000000	21.0		
06.08.2005	3.656803	17.9	0.000000	16.9		
07.08.2005	2.742617	18.9	0.000000	18.9		
08.08.2005	2.742631	19.4	0.000000	20.3		
09.08.2005	2.742646	17.6	0.000000	17.4		
10.08.2005	1.828440	13.3	0.000000	12.2		
11.08.2005	0.914225	12.1	0.000000	12.8		
12.08.2005	0.914230	19.3	0.000000	19.7		
13.08.2005	0.914235	21.5	0.000000	20.5		
14.08.2005	1.828479	22.6	1.530424	21.3		
15.08.2005	0.000000	22.2	1.530411	21.1		
16.08.2005	1.828498	23.3	0.000000	22.6		
17.08.2005	1.828508	18.8	0.765192	17.7		
18.08.2005	3.657034	18.6	0.000000	17.8		
19.08.2005	4.571317	15.6	0.000000	14.2		
20.08.2005	1.828536	13.9	0.000000	12.1		
21.08.2005	2.742819	10.7	0.000000	10.9		
22.08.2005	3.657111	12.3	0.000000	10.8		
23.08.2005	1.828565	9.5	0.000000	9.9		
24.08.2005	3.657150	9.4	0.000000	8.1		
25.08.2005	1.828584	8.8	0.000000	9.2		
26.08.2005	0.914297	13.3	0.000000	14.1		
27.08.2005	0.000000	13.4	0.765126	14.4		
28.08.2005	4.571533	12.2	0.000000	12.3		
29.08.2005	3.657246	12.1	0.765113	12.3		
30.08.2005	2.742949	12.6	0.765107	12.7		
31.08.2005	3.657284	12.8	1.530200	11.7		

Data	Chelyabinsk		Yekaterinburg		Barnaul	
	RI rates	Temperature	RI rates	Temperature	RI rates	Temperature
01.09.2005	5.485955	13.5	0.765094	13.4		
02.09.2005	4.571654	12.2	1.530174	12.7		
03.09.2005	2.743007	11.4	1.530161	11.6		
04.09.2005	1.828681	11.2	0.000000	12.2		
05.09.2005	0.914345	12.6	1.530135	11.3		
06.09.2005	1.828700	12.4	0.765061	11.4		
07.09.2005	1.828710	10.2	0.765054	9.9		
08.09.2005	6.400517	11.8	0.000000	11.4		
09.09.2005	2.743093	13.9	0.765041	13.4		
10.09.2005	3.657477	18.1	0.000000	15.6		
11.09.2005	0.914374	12.4	1.530056	10.0		
12.09.2005	3.657515	9.1	1.530043	6.2		
13.09.2005	2.743151	10.2	0.000000	7.5		
14.09.2005	2.743165	13.4	0.765008	9.9		
15.09.2005	0.000000	14.6	0.765002	12.8		
16.09.2005	0.914398	16.0	0.764995	16.6		
17.09.2005	3.657611	16.5	0.000000	18.8		
18.09.2005	2.743223	17.6	0.000000	19.1		
19.09.2005	1.828825	11.8	0.000000	7.0		
20.09.2005	0.914417	5.6	0.764969	7.2		
21.09.2005	0.000000	7.2	0.000000	6.4		
22.09.2005	3.657708	10.0	0.000000	9.4		
23.09.2005	3.657727	9.9	0.764949	8.8		
24.09.2005	0.914437	8.9	0.764942	7.6		
25.09.2005	1.828883	6.1	0.000000	4.4		
26.09.2005	0.914446	7.8	0.764929	7.1		
27.09.2005	4.572255	9.5	0.000000	9.3		
28.09.2005	1.828912	10.6	0.000000	11.3		
29.09.2005	4.572303	9.9	0.764910	8.7		
30.09.2005	0.914465	9.8	0.000000	8.1		
01.10.2005	2.743411	9.9	0.764896	8.7		
02.10.2005	0.914475	11.9	0.764890	11.4		
03.10.2005	2.743440	12.6	0.764883	12.1		
04.10.2005	3.657939	10.8	0.764877	11.3		
05.10.2005	1.828979	9.0	0.764870	8.1		
06.10.2005	3.657977	10.1	0.000000	8.1		
07.10.2005	2.743497	8.7	1.529714	8.6		
08.10.2005	3.658016	4.2	0.764850	3.6		
09.10.2005	0.914509	6.0	1.529688	4.8		
10.10.2005	0.914514	6.2	0.764837	5.3		
11.10.2005	0.914518	6.4	2.294492	6.8		

Data	Chelyabinsk		Yekaterinburg		Barnaul	
	RI rates	Temperature	RI rates	Temperature	RI rates	Temperature
12.10.2005	0.914523	7.1	0.000000	7.9		
13.10.2005	2.743584	8.9	0.764818	9.2		
14.10.2005	4.572664	8.8	0.764811	8.2		
15.10.2005	0.000000	6.9	1.529609	7.4		
16.10.2005	0.000000	5.7	1.529596	2.8		
17.10.2005	0.000000	7.9	1.529583	6.4		
18.10.2005	3.658208	10.6	0.764785	9.8		
19.10.2005	2.743671	11.9	0.764778	8.8		
20.10.2005	2.743685	3.5	0.764772	-0.2		
21.10.2005	0.000000	-0.6	0.000000	-3.3		
22.10.2005	0.000000	-3.1	0.000000	-2.7		
23.10.2005	0.000000	2.7	2.294256	3.3		
24.10.2005	2.743743	5.7	0.000000	6.4		
25.10.2005	3.658343	7.6	0.764739	9.3		
26.10.2005	1.829181	3.3	0.000000	1.3		
27.10.2005	5.487572	-0.9	0.000000	-4.6		
28.10.2005	2.743800	-6.2	0.764719	-5.6		
29.10.2005	0.914605	-5.6	0.764712	-3.7		
30.10.2005	1.829220	-1.0	0.000000	-0.6		
31.10.2005	1.829229	2.1	0.000000	1.7		
01.11.2005	3.658478	3.9	0.000000	2.4		
02.11.2005	2.743873	3.4	0.764686	3.3		
03.11.2005	3.658516	2.8	0.000000	1.7		
04.11.2005	0.914634	2.5	1.529346	1.9		
05.11.2005	1.829277	3.1	0.000000	2.7		
06.11.2005	0.914643	4.9	0.764660	3.7		
07.11.2005	0.914648	2.2	1.529307	0.1		
08.11.2005	1.829306	-0.8	0.764647	-3.4		
09.11.2005	1.829316	-4.9	0.000000	-5.9		
10.11.2005	1.829325	-7.9	0.000000	-6.1		
11.11.2005	1.829335	-5.4	0.000000	-2.4		
12.11.2005	4.573362	-2.5	0.000000	-1.2		
13.11.2005	4.573386	-1.7	0.000000	-0.4		
14.11.2005	0.914682	-0.3	0.000000	0.2		
15.11.2005	3.658747	-0.3	0.000000	-0.7		
16.11.2005	1.829383	-1.5	0.764594	-1.4		
17.11.2005	3.658786	-3.2	2.293763	-1.8		
18.11.2005	5.488207	-3.7	1.529162	-1.4		
19.11.2005	0.914706	-5.0	0.764575	-2.6		
20.11.2005	1.829422	-6.5	0.764568	-6.4		
21.11.2005	1.829431	-5.6	1.529123	-6.9		

Data	Chelyabinsk		Yekaterinburg		Barnaul	
	RI rates	Temperature	RI rates	Temperature	RI rates	Temperature
22.11.2005	1.829441	-5.8	0.764555	-7.3		
23.11.2005	1.829451	-6.2	0.000000	-5.9		
24.11.2005	0.914730	-3.2	0.764542	-3.1		
25.11.2005	6.403144	-1.8	0.000000	-0.5		
26.11.2005	4.573699	-2.6	0.764529	-2.1		
27.11.2005	3.658978	-1.3	0.000000	-0.7		
28.11.2005	8.232744	-0.9	0.764515	0.3		
29.11.2005	6.403279	-0.8	0.764509	-0.4		
30.11.2005	7.318072	-6.2	3.058009	-4.2		
01.12.2005	0.000000	-4.8	0.000000	-6.7		
02.12.2005	1.829537	-5.9	1.528978	-5.2		
03.12.2005	4.573867	-7.1	0.000000	-8.5		
04.12.2005	0.914778	-6.7	0.764476	-9.3		
05.12.2005	6.403481	-13.4	0.000000	-13.9		
06.12.2005	3.659152	-8.8	2.293389	-8.7		
07.12.2005	0.914793	-8.1	0.000000	-6.9		
08.12.2005	1.829595	-11.2	0.000000	-9.8		
09.12.2005	0.914802	-8.8	1.528886	-8.2		
10.12.2005	4.574036	-6.7	0.000000	-6.4		
11.12.2005	1.829624	-8.0	0.764430	-7.1		
12.12.2005	4.574084	-10.1	1.528847	-9.3		
13.12.2005	1.829643	-9.3	0.000000	-11.3		
14.12.2005	0.000000	-3.6	0.764410	-3.8		
15.12.2005	1.829662	-6.5	1.528808	-7.7		
16.12.2005	3.659344	-7.8	0.764397	-6.9		
17.12.2005	3.659363	-4.3	0.000000	-5.2		
18.12.2005	2.744537	-4.9	1.528768	-6.1		
19.12.2005	0.914850	-9.2	0.764378	-8.5		
20.12.2005	4.574276	-8.3	0.000000	-7.9		
21.12.2005	7.318881	-13.5	0.764364	-9.4		
22.12.2005	6.404054	-9.9	2.293074	-10.6		
23.12.2005	4.574349	-13.8	0.764351	-12.1		
24.12.2005	4.574373	-15.5	0.764345	-8.2		
25.12.2005	2.744638	-15.9	2.293015	-11.0		
26.12.2005	0.914884	-11.6	1.528663	-11.4		
27.12.2005	6.404223	-13.4	3.057300	-15.6		
28.12.2005	1.829788	-14.9	3.057274	-16.7		
29.12.2005	1.829797	-10.6	0.764312	-9.9		
30.12.2005	3.659614	-10.4	3.057222	-9.9		
31.12.2005	0.914908	-17.1	0.000000	-18.2		
01.01.2006	5.489478	-17.4	0.764292	-17.7	0.000000	-26.9

Data	Chelyabinsk		Yekaterinburg		Barnaul	
	RI rates	Temperature	RI rates	Temperature	RI rates	Temperature
02.01.2006	3.659666	-5.0	0.000000	-6.2	0.000000	-30.8
03.01.2006	3.659680	-6.7	1.528542	-8.1	0.000000	-28.9
04.01.2006	1.829847	-10.0	3.057040	-11.1	0.000000	-17.9
05.01.2006	5.489561	-11.1	0.764249	-16.2	0.000000	-13.4
06.01.2006	7.319442	-22.3	2.292716	-24.6	0.000000	-13.2
07.01.2006	1.829867	-21.9	0.764228	-19.3	0.000000	-21.7
08.01.2006	2.744811	-20.4	2.292652	-21.4	0.000000	-20.1
09.01.2006	2.744822	-25.9	1.528413	-29.4	0.000000	-17.4
10.01.2006	4.574720	-23.5	5.349370	-25.4	0.000000	-27.2
11.01.2006	3.659790	-11.1	2.292555	-14.6	0.000000	-30.4
12.01.2006	4.574755	-8.9	2.292523	-10.7	0.000000	-27.1
13.01.2006	1.829909	-6.0	3.820818	-5.3	0.000000	-29.8
14.01.2006	5.489747	-4.7	3.820764	-9.2	0.000000	-14.4
15.01.2006	7.319690	-26.6	8.405564	-29.2	0.000000	-10.6
16.01.2006	1.829929	-36.5	1.528263	-35.5	0.000000	-8.2
17.01.2006	6.404777	-28.7	1.528241	-30.1	0.000000	-7.7
18.01.2006	5.489829	-30.9	1.528220	-31.0	0.000000	-6.6
19.01.2006	7.319800	-33.3	3.056397	-32.2	0.000000	-9.8
20.01.2006	1.829957	-34.8	4.584532	-33.9	0.000000	-31.3
21.01.2006	2.744946	-31.9	3.820389	-30.2	0.000000	-33.2
22.01.2006	2.744956	-24.7	2.292201	-24.0	0.000000	-32.9
23.01.2006	2.744966	-34.1	2.292169	-33.8	3.311517	-29.6
24.01.2006	1.829984	-31.7	2.292137	-30.4	0.000000	-31.5
25.01.2006	9.149956	-26.8	3.820175	-26.0	0.000000	-38.8
26.01.2006	3.659996	-21.1	5.348170	-19.7	0.000000	-34.2
27.01.2006	8.235023	-20.2	6.876122	-21.3	0.000000	-32.6
28.01.2006	7.320048	-20.8	6.112023	-25.1	0.000000	-32.2
29.01.2006	4.575047	-19.9	4.583953	-20.8	0.000000	-24.4
30.01.2006	5.490077	-17.9	5.347870	-17.1	0.000000	-23.7
31.01.2006	7.320130	-16.9	6.875736	-16.9	1.656005	-21.8
01.02.2006	9.150197	-16.8	3.055840	-15.7	0.000000	-22.1
02.02.2006	10.980278	-15.4	3.055797	-16.6	0.000000	-20.2
03.02.2006	8.235240	-16.2	3.819693	-18.3	0.000000	-10.0
04.02.2006	7.320240	-5.9	3.819639	-17.3	0.000000	-17.7
05.02.2006	3.660134	-16.9	4.583503	-21.3	0.000000	-16.1
06.02.2006	5.490222	-17.4	4.583439	-22.0	0.000000	-6.5
07.02.2006	3.660162	-13.1	8.402853	-17.6	1.656220	-7.5
08.02.2006	10.065482	-10.4	8.402735	-13.3	1.656251	-7.3
09.02.2006	6.405331	-19.1	5.347120	-20.6	0.000000	-2.0
10.02.2006	1.830101	-16.8	12.221818	-17.8	0.000000	-1.9
11.02.2006	2.745162	-12.6	5.346970	-14.2	0.000000	-14.2

Data	Chelyabinsk		Yekaterinburg		Barnaul	
	RI rates	Temperature	RI rates	Temperature	RI rates	Temperature
12.02.2006	3.660230	-13.7	3.819211	-15.0	0.000000	-16.6
13.02.2006	7.320488	-13.9	11.457472	-9.6	0.000000	-16.9
14.02.2006	7.320516	-12.6	9.165849	-8.9	0.000000	-20.2
15.02.2006	2.745204	-11.6	2.291430	-10.9	0.000000	-15.8
16.02.2006	8.235642	-15.8	7.637993	-8.2	0.000000	-14.0
17.02.2006	12.811047	-10.7	9.165464	-7.8	0.000000	-10.9
18.02.2006	10.065861	-13.7	5.346445	-12.5	0.000000	-9.9
19.02.2006	5.490490	-18.1	10.692741	-18.0	0.000000	-16.1
20.02.2006	12.811192	-16.3	6.873809	-16.8	3.313242	-17.3
21.02.2006	10.981063	-10.3	5.346221	-15.2	0.000000	-17.3
22.02.2006	5.490552	-14.1	6.873616	-14.2	0.000000	-17.4
23.02.2006	4.575477	-17.2	5.346071	-14.9	0.000000	-18.3
24.02.2006	7.320791	-5.8	9.164564	-4.5	0.000000	-15.0
25.02.2006	4.575512	-2.9	10.691842	-6.1	0.000000	-15.6
26.02.2006	8.235952	-2.3	6.109538	-4.9	0.000000	-13.7
27.02.2006	8.235983	-7.6	9.164179	-3.0	1.656837	-11.4
28.02.2006	18.302253	-6.3	6.873038	-0.8	4.970603	-8.9
01.03.2006	10.981393	-5.3	5.345621	-3.9	1.656899	-5.1
02.03.2006	10.066315	-9.9	10.691092	-10.2	3.313859	-5.9
03.03.2006	4.575615	-10.1	9.163665	-9.6	0.000000	-11.1
04.03.2006	5.490758	-7.9	8.399908	-9.4	0.000000	-5.7
05.03.2006	4.575649	-8.1	4.581704	-7.3	1.657022	-9.8
06.03.2006	10.981600	-5.6	9.163280	-7.1	0.000000	-5.9
07.03.2006	12.811914	-7.4	12.217535	-9.8	0.000000	-3.4
08.03.2006	3.660561	-4.7	4.581511	-5.5	0.000000	-10.2
09.03.2006	9.151436	0.7	3.054298	1.6	0.000000	-12.6
10.03.2006	8.236324	1.7	6.108511	2.4	0.000000	-10.5
11.03.2006	7.321204	-2.4	6.871978	-4.8	1.657207	-6.8
12.03.2006	1.830308	-6.1	6.108339	-6.8	0.000000	-3.5
13.03.2006	8.236416	-3.8	8.398849	-5.6	1.657269	-3.3
14.03.2006	5.490965	-5.6	5.344647	-8.9	0.000000	-6.2
15.03.2006	12.812300	-1.7	6.108083	-1.4	0.000000	-10.8
16.03.2006	8.236509	-0.9	5.344497	-1.3	0.000000	-8.4
17.03.2006	10.066883	-1.8	7.634889	-3.5	1.657392	-3.4
18.03.2006	10.066921	-3.2	6.871304	-4.0	0.000000	-3.7
19.03.2006	2.745534	-1.5	8.398143	-1.4	1.657454	-5.1
20.03.2006	8.236633	0.5	8.398025	1.7	4.972454	-5.7
21.03.2006	14.642959	1.8	9.924800	2.6	0.000000	-5.8
22.03.2006	2.745565	-3.6	5.344048	-4.7	3.315093	-2.8
23.03.2006	6.406343	-4.1	9.924521	-6.2	0.000000	-1.8
24.03.2006	7.321562	0.9	7.634140	-1.4	0.000000	-1.7

Data	Chelyabinsk		Yekaterinburg		Barnaul	
	RI rates	Temperature	RI rates	Temperature	RI rates	Temperature
25.03.2006	3.660795	2.3	6.107227	2.1	0.000000	-0.7
26.03.2006	3.660809	-2.2	5.343748	-4.4	0.000000	2.7
27.03.2006	6.406439	-2.4	9.160583	-4.3	0.000000	-1.0
28.03.2006	7.321672	-4.7	6.870341	-6.2	1.657732	3.4
29.03.2006	8.236912	-0.8	6.870245	-3.0	0.000000	-2.6
30.03.2006	11.897807	4.8	3.816749	2.1	0.000000	-0.7
31.03.2006	10.067413	2.3	4.580035	0.9	0.000000	1.9
01.04.2006	7.321782	2.1	3.053314	-1.2	0.000000	-0.4
02.04.2006	5.491357	5.7	9.159813	2.7	0.000000	-4.6
03.04.2006	10.067526	3.3	6.869764	1.9	1.657917	-0.4
04.04.2006	12.813264	0.1	6.106371	-0.9	1.657948	-0.4
05.04.2006	7.321892	-3.9	4.579714	-5.2	0.000000	-6.0
06.04.2006	6.406680	-5.6	4.579650	-5.6	1.658009	-6.5
07.04.2006	13.728652	-7.6	9.159171	-6.2	0.000000	-5.0
08.04.2006	1.830494	-5.8	5.342775	-3.6	0.000000	-5.3
09.04.2006	2.745751	1.6	3.816214	2.0	0.000000	-6.6
10.04.2006	6.406776	4.1	3.816161	2.0	8.290665	-5.4
11.04.2006	7.322058	5.6	3.816108	3.1	3.316328	-3.9
12.04.2006	8.237346	8.2	5.342476	4.6	0.000000	-4.2
13.04.2006	8.237377	11.5	3.816001	6.4	0.000000	-1.4
14.04.2006	6.406873	8.2	2.289568	5.1	1.658257	3.9
15.04.2006	1.830542	7.8	2.289536	7.4	0.000000	2.2
16.04.2006	5.491647	9.6	5.342176	7.6	0.000000	0.8
17.04.2006	5.491667	6.6	3.052629	2.2	1.658349	3.8
18.04.2006	2.745844	3.1	5.342027	0.1	4.975140	10.8
19.04.2006	8.237563	6.4	4.578816	5.9	1.658411	0.8
20.04.2006	7.322306	9.2	3.815626	11.2	1.658442	0.9
21.04.2006	8.237625	7.1	5.341802	4.9	0.000000	4.9
22.04.2006	2.745885	7.5	2.289312	5.1	0.000000	9.0
23.04.2006	3.661194	10.8	3.815466	7.8	0.000000	3.9
24.04.2006	5.491812	12.2	3.052330	4.4	1.658565	4.7
25.04.2006	3.661222	3.2	3.052287	2.2	1.658596	8.3
26.04.2006	4.576544	0.5	3.815306	1.6	0.000000	8.9
27.04.2006	4.576561	1.1	2.289151	0.6	1.658658	1.6
28.04.2006	8.237842	0.1	7.630398	-0.5	0.000000	2.1
29.04.2006	3.661277	0.4	1.526058	0.7	0.000000	1.6
30.04.2006	1.830645	2.6	3.052074	3.7	0.000000	2.4
01.05.2006	0.915326	6.5	2.289023	6.2	0.000000	1.3
02.05.2006	5.491977	11.1	3.051988	10.7	0.000000	2.5
03.05.2006	5.491998	9.4	3.814932	8.6	0.000000	3.4
04.05.2006	7.322691	2.7	5.340830	3.1	0.000000	2.3

Data	Chelyabinsk		Yekaterinburg		Barnaul	
	RI rates	Temperature	RI rates	Temperature	RI rates	Temperature
05.05.2006	1.830680	3.9	1.525930	4.4	0.000000	3.6
06.05.2006	2.746030	9.5	6.866589	7.9	0.000000	4.2
07.05.2006	3.661387	12.1	3.814718	11.3	0.000000	6.0
08.05.2006	5.492101	13.3	3.051732	11.7	0.000000	7.8
09.05.2006	5.492122	15.5	3.814611	15.3	0.000000	8.9
10.05.2006	7.322856	17.7	2.288735	17.6	0.000000	14.2
11.05.2006	8.238245	20.1	2.288703	19.1	0.000000	16.6
12.05.2006	5.492184	16.3	4.577341	12.0	0.000000	18.8
13.05.2006	3.661470	14.7	5.340156	9.6	0.000000	21.2
14.05.2006	2.746112	5.0	3.051475	6.1	0.000000	13.1
15.05.2006	3.661497	11.1	6.102865	10.5	0.000000	11.2
16.05.2006	6.407644	17.2	0.762847	16.6	0.000000	11.3
17.05.2006	2.746143	13.7	7.628368	9.5	0.000000	14.2
18.05.2006	1.830769	13.8	6.102609	9.7	0.000000	16.4
19.05.2006	4.576940	7.2	1.525631	4.0	0.000000	16.0
20.05.2006	4.576957	10.6	3.814024	9.1	0.000000	16.9
21.05.2006	2.746185	11.6	4.576764	8.3	0.000000	6.0
22.05.2006	2.746195	16.1	0.762783	14.7	0.000000	9.6
23.05.2006	4.577009	20.8	3.051091	17.7	0.000000	11.7
24.05.2006	3.661621	15.9	2.288286	14.1	0.000000	17.2
25.05.2006	7.323270	14.4	2.288254	13.9	0.000000	22.2
26.05.2006	7.323297	15.4	2.288222	13.8	0.000000	19.6
27.05.2006	5.492494	16.9	2.288190	15.8	0.000000	15.2
28.05.2006	0.000000	22.3	2.288158	14.4	0.000000	13.4
29.05.2006	6.407957	20.8	1.525417	17.8	0.000000	12.2
30.05.2006	6.407982	17.2	5.338885	17.0	0.000000	9.0
31.05.2006	8.238864	13.6	1.525374	12.3	0.000000	13.0
01.06.2006	3.661731	12.2	2.288030	10.8	0.000000	17.2
02.06.2006	3.661745	17.7	0.000000	15.9	0.000000	17.1
03.06.2006	4.577199	19.4	4.575931	17.9	0.000000	18.3
04.06.2006	1.830886	16.7	1.525289	16.1	0.000000	19.2
05.06.2006	3.661786	19.1	1.525268	19.3	0.000000	20.7
06.06.2006	6.408150	25.2	3.813116	25.6	0.000000	21.4
07.06.2006	3.661814	23.4	3.050450	25.2	0.000000	23.7
08.06.2006	0.915457	22.5	0.762602	21.7	0.000000	24.6
09.06.2006	0.915460	21.4	0.762591	21.9	0.000000	24.2
10.06.2006	0.915464	23.7	0.762580	23.8	0.000000	19.7
11.06.2006	1.830935	21.3	0.762570	19.1	0.000000	19.4
12.06.2006	0.000000	14.3	0.000000	13.1	1.660081	24.3
13.06.2006	0.000000	12.8	0.762548	10.9	0.000000	24.2
14.06.2006	0.915478	12.9	0.762538	13.5	0.000000	17.4

Data	Chelyabinsk		Yekaterinburg		Barnaul	
	RI rates	Temperature	RI rates	Temperature	RI rates	Temperature
15.06.2006	0.915481	22.3	0.762527	23.2	0.000000	10.8
16.06.2006	1.830969	22.6	1.525033	21.4	0.000000	12.7
17.06.2006	0.000000	21.4	0.762506	22.7	0.000000	17.4
18.06.2006	0.915491	23.3	1.524990	20.5	0.000000	20.2
19.06.2006	1.830990	18.9	0.762484	17.1	1.660298	21.6
20.06.2006	0.915498	15.6	0.762474	13.6	0.000000	22.4
21.06.2006	4.577508	14.0	0.762463	16.2	0.000000	23.8
22.06.2006	1.831010	15.2	0.000000	17.5	0.000000	25.9
23.06.2006	3.662034	19.5	0.762442	20.7	0.000000	26.4
24.06.2006	0.915512	16.6	0.000000	17.8	0.000000	24.8
25.06.2006	0.915515	17.4	0.000000	18.3	0.000000	23.3
26.06.2006	1.831038	17.6	0.762410	18.6	0.000000	23.4
27.06.2006	0.000000	18.9	0.000000	19.6	0.000000	22.7
28.06.2006	1.831052	21.4	0.000000	21.7	0.000000	21.4
29.06.2006	1.831059	19.8	0.000000	19.6	0.000000	23.1
30.06.2006	1.831065	21.9	1.524734	22.2	0.000000	22.8
01.07.2006	0.000000	21.7	0.000000	19.1	0.000000	22.2
02.07.2006	0.000000	14.2	0.000000	10.6	0.000000	21.5
03.07.2006	0.000000	8.9	0.000000	7.1	0.000000	23.9
04.07.2006	1.831093	9.6	0.000000	8.2	0.000000	26.3
05.07.2006	0.000000	12.3	0.000000	11.9	0.000000	20.7
06.07.2006	6.408874	15.1	0.000000	13.6	0.000000	13.6
07.07.2006	0.915557	11.0	0.762292	11.2	0.000000	17.5
08.07.2006	0.915560	12.1	0.000000	13.5	0.000000	18.1
09.07.2006	0.915564	14.6	0.000000	16.4	0.000000	12.4
10.07.2006	0.000000	17.1	0.000000	18.9	0.000000	14.3
11.07.2006	0.915571	18.5	0.000000	21.1	0.000000	16.3
12.07.2006	2.746722	20.3	0.762239	22.5	0.000000	18.3
13.07.2006	0.915577	21.4	0.762228	23.0	0.000000	20.6
14.07.2006	1.831162	21.8	0.762218	23.9	0.000000	21.6
15.07.2006	0.000000	23.4	0.762207	23.9	0.000000	18.0
16.07.2006	0.000000	22.4	0.000000	21.4	0.000000	18.3
17.07.2006	2.746774	21.4	1.524371	21.4	1.661165	20.3
18.07.2006	0.915595	22.3	0.762175	22.1	0.000000	20.5
19.07.2006	2.746794	24.8	0.000000	25.1	0.000000	18.9
20.07.2006	2.746805	19.2	0.000000	19.5	1.661258	18.6
21.07.2006	0.000000	18.8	0.000000	15.8	0.000000	20.8
22.07.2006	0.000000	11.4	0.000000	10.8	0.000000	24.4
23.07.2006	1.831224	9.7	0.000000	7.8	0.000000	19.4
24.07.2006	0.000000	13.3	0.000000	10.7	0.000000	14.7
25.07.2006	0.915619	15.4	0.000000	14.6	0.000000	16.6

Data	Chelyabinsk		Yekaterinburg		Barnaul	
	RI rates	Temperature	RI rates	Temperature	RI rates	Temperature
26.07.2006	2.746867	14.4	0.762090	11.7	0.000000	18.2
27.07.2006	2.746877	16.7	0.762079	16.0	0.000000	17.7
28.07.2006	1.831258	15.8	1.524137	12.8	0.000000	15.7
29.07.2006	0.000000	10.7	0.000000	11.2	0.000000	21.1
30.07.2006	0.915636	13.6	0.000000	12.3	0.000000	22.2
31.07.2006	0.000000	11.1	0.762036	9.8	0.000000	19.9
01.08.2006	0.000000	11.8	1.524052	10.7	0.000000	21.3
02.08.2006	0.915646	14.2	0.000000	12.3	0.000000	19.1
03.08.2006	0.000000	13.7	0.762004	12.6	1.661692	20.1
04.08.2006	0.915653	14.3	0.761994	13.2	0.000000	19.0
05.08.2006	0.000000	13.7	0.000000	14.6	0.000000	15.9
06.08.2006	0.000000	14.7	0.000000	14.5	0.000000	16.6
07.08.2006	1.831327	13.7	0.761962	12.8	0.000000	15.0
08.08.2006	0.000000	11.8	0.000000	12.6	0.000000	14.2
09.08.2006	0.915671	12.8	0.000000	12.1	0.000000	15.3
10.08.2006	0.915674	14.1	1.523860	13.6	0.000000	14.6
11.08.2006	2.747032	17.7	0.000000	17.1	0.000000	14.6
12.08.2006	0.915681	16.0	0.000000	15.6	0.000000	16.4
13.08.2006	0.000000	13.7	0.761898	12.6	0.000000	19.2
14.08.2006	0.915688	12.7	1.523775	12.6	0.000000	19.5
15.08.2006	0.915691	12.5	0.000000	12.4	0.000000	11.8
16.08.2006	0.915695	16.9	0.761866	16.3	0.000000	12.9
17.08.2006	1.831396	20.0	0.000000	20.1	0.000000	15.1
18.08.2006	1.831403	20.7	0.761845	19.4	0.000000	13.3
19.08.2006	0.000000	21.3	0.761834	19.9	0.000000	14.7
20.08.2006	1.831417	21.1	1.523647	20.9	0.000000	16.0
21.08.2006	4.578559	16.6	1.523625	16.7	0.000000	20.6
22.08.2006	3.662861	14.9	0.761802	15.7	0.000000	19.2
23.08.2006	3.662875	14.9	0.000000	15.4	0.000000	14.6
24.08.2006	0.915722	15.1	0.761781	14.9	0.000000	13.4
25.08.2006	2.747177	15.5	1.523540	16.7	0.000000	13.7
26.08.2006	0.000000	16.8	1.523519	16.3	0.000000	9.8
27.08.2006	0.915733	17.8	0.761749	16.3	0.000000	12.5
28.08.2006	0.915736	16.1	0.761738	15.1	0.000000	14.0
29.08.2006	0.915739	11.4	0.000000	11.6	0.000000	15.3
30.08.2006	1.831486	12.1	0.000000	12.1	0.000000	7.6
31.08.2006	1.831493	17.6	0.761706	14.4	0.000000	7.8
01.09.2006	2.747249	21.2	0.761696	21.4	0.000000	11.4
02.09.2006	0.000000	22.5	1.523370	22.2	0.000000	14.4
03.09.2006	0.000000	20.4	0.000000	17.5	0.000000	19.1
04.09.2006	3.663040	19.6	1.523327	18.0	0.000000	12.9

Data	Chelyabinsk		Yekaterinburg		Barnaul	
	RI rates	Temperature	RI rates	Temperature	RI rates	Temperature
05.09.2006	1.831527	20.9	0.761653	21.7	0.000000	8.5
06.09.2006	5.494602	19.4	0.761642	21.4	0.000000	10.8
07.09.2006	3.663082	20.7	0.000000	19.9	0.000000	14.1
08.09.2006	1.831548	16.7	0.000000	16.3	0.000000	16.9
09.09.2006	2.747332	14.9	0.000000	14.2	0.000000	21.4
10.09.2006	1.831562	16.2	0.000000	14.4	0.000000	21.3
11.09.2006	3.663137	13.7	0.761589	12.9	0.000000	16.6
12.09.2006	0.000000	10.6	0.000000	10.4	0.000000	17.2
13.09.2006	0.000000	7.0	0.000000	6.4	0.000000	17.4
14.09.2006	2.747384	8.1	1.523114	7.7	0.000000	13.2
15.09.2006	1.831596	11.1	0.761546	10.3	0.000000	9.8
16.09.2006	0.915801	4.8	0.000000	3.6	0.000000	14.7
17.09.2006	0.000000	2.6	1.523050	1.9	0.000000	17.1
18.09.2006	0.915808	2.2	0.761515	0.8	0.000000	11.6
19.09.2006	2.747435	7.6	0.000000	6.7	0.000000	6.6
20.09.2006	2.747446	10.9	0.000000	10.7	0.000000	7.8
21.09.2006	0.915819	10.9	1.522965	11.3	0.000000	7.4
22.09.2006	1.831644	13.7	0.761472	13.6	0.000000	8.7
23.09.2006	2.747477	12.2	0.761461	10.6	0.000000	10.4
24.09.2006	0.915829	6.4	3.045803	6.5	0.000000	12.9
25.09.2006	2.747497	15.3	1.522880	12.7	0.000000	5.2
26.09.2006	0.915836	4.4	1.522859	4.3	0.000000	11.7
27.09.2006	0.915839	1.2	0.761419	1.9	0.000000	8.9
28.09.2006	0.915843	5.6	0.761408	4.1	0.000000	11.6
29.09.2006	0.915846	12.8	1.522795	11.3	0.000000	9.1
30.09.2006	1.831699	13.1	0.761387	8.6	1.663493	13.8
01.10.2006	1.831706	11.2	0.000000	2.9	0.000000	10.8
02.10.2006	0.000000	3.6	0.761366	0.9	0.000000	12.3
03.10.2006	0.000000	1.2	1.522710	-0.6	0.000000	17.5
04.10.2006	2.747591	1.8	0.000000	-0.2	0.000000	3.6
05.10.2006	0.915867	6.7	1.522667	5.7	0.000000	-0.8
06.10.2006	1.831741	9.6	2.283969	6.8	0.000000	0.2
07.10.2006	0.000000	4.3	0.000000	2.6	0.000000	4.4
08.10.2006	1.831755	2.2	2.283905	0.6	0.000000	2.1
09.10.2006	3.663523	-0.7	0.761291	-1.4	0.000000	3.8
10.10.2006	1.831768	-0.7	0.761280	-0.2	0.000000	5.5
11.10.2006	0.000000	1.0	0.761270	1.1	0.000000	0.8
12.10.2006	2.747673	3.0	0.000000	2.8	0.000000	-1.2
13.10.2006	2.747684	4.7	0.761248	4.7	0.000000	-2.2
14.10.2006	5.495388	3.2	0.761238	3.1	0.000000	0.6
15.10.2006	2.747704	0.6	0.761227	1.4	0.000000	2.7

Data	Chelyabinsk		Yekaterinburg		Barnaul	
	RI rates	Temperature	RI rates	Temperature	RI rates	Temperature
16.10.2006	1.831810	0.9	2.283650	-0.3	1.663991	4.0
17.10.2006	0.915908	-1.4	0.761206	-1.7	0.000000	1.5
18.10.2006	2.747735	1.1	0.000000	1.8	0.000000	0.8
19.10.2006	3.663661	3.4	0.761185	2.0	0.000000	0.3
20.10.2006	2.747756	-0.3	0.761174	-2.0	0.000000	2.6
21.10.2006	1.831844	-6.1	0.761163	-6.6	0.000000	2.2
22.10.2006	1.831851	-2.9	0.000000	-3.3	0.000000	-4.7
23.10.2006	2.747787	5.3	0.000000	5.5	3.328417	-6.6
24.10.2006	0.000000	8.2	0.761131	8.8	0.000000	-2.1
25.10.2006	1.831872	10.4	0.000000	10.4	0.000000	3.7
26.10.2006	3.663757	8.9	0.000000	9.1	0.000000	6.5
27.10.2006	3.663771	8.2	0.761100	4.8	0.000000	7.5
28.10.2006	1.831892	9.4	0.761089	7.7	0.000000	7.9
29.10.2006	2.747849	9.8	0.761078	6.8	0.000000	7.9
30.10.2006	5.495719	1.8	0.761068	1.7	0.000000	9.1
31.10.2006	1.831913	-0.8	0.761057	0.7	0.000000	8.9
01.11.2006	6.411720	0.9	0.000000	0.2	0.000000	9.4
02.11.2006	0.915963	1.6	3.805179	2.5	0.000000	0.2
03.11.2006	2.747901	2.9	1.522050	5.6	1.664551	1.7
04.11.2006	1.831941	2.9	3.805073	5.9	0.000000	2.1
05.11.2006	0.915974	2.3	1.522008	5.9	0.000000	2.4
06.11.2006	0.915977	6.8	0.000000	7.6	3.329288	2.4
07.11.2006	3.663923	-3.8	0.000000	-4.4	0.000000	4.7
08.11.2006	2.747953	-6.2	1.521944	-3.6	1.664706	7.1
09.11.2006	3.663950	-3.2	1.521923	-3.2	0.000000	9.1
10.11.2006	0.000000	0.6	2.282852	-2.0	0.000000	2.8
11.11.2006	0.915995	6.2	4.565641	3.3	0.000000	2.4
12.11.2006	0.915998	4.5	0.760930	3.2	0.000000	2.8
13.11.2006	1.832003	-3.4	0.000000	-3.5	0.000000	0.7
14.11.2006	2.748015	-6.2	1.521816	-5.7	0.000000	0.8
15.11.2006	1.832017	-3.3	1.521795	-4.3	0.000000	-2.0
16.11.2006	2.748035	-0.4	3.043548	-1.4	0.000000	-3.9
17.11.2006	1.832030	-0.7	1.521753	-3.2	0.000000	-2.8
18.11.2006	1.832037	-8.3	2.282597	-12.2	0.000000	-2.5
19.11.2006	0.000000	-16.9	0.760855	-20.1	0.000000	-7.3
20.11.2006	1.832051	-22.4	0.760844	-22.9	0.000000	-2.4
21.11.2006	3.664116	-18.7	4.565003	-17.7	0.000000	-4.5
22.11.2006	2.748097	-19.2	3.043293	-19.3	0.000000	-8.3
23.11.2006	5.496215	-13.3	1.521625	-12.1	0.000000	-15.7
24.11.2006	2.748118	-4.8	1.521604	-5.6	1.665205	-19.5
25.11.2006	2.748128	-16.4	1.521583	-16.6	0.000000	-13.6

Data	Chelyabinsk		Yekaterinburg		Barnaul	
	RI rates	Temperature	RI rates	Temperature	RI rates	Temperature
26.11.2006	3.664185	-23.6	2.282342	-21.2	0.000000	-11.4
27.11.2006	0.916050	-25.7	0.760770	-24.6	0.000000	-13.4
28.11.2006	2.748159	-27.3	0.760759	-24.3	0.000000	-15.9
29.11.2006	1.832113	-20.5	4.564493	-20.4	1.665360	-15.4
30.11.2006	4.580300	-21.9	2.282215	-16.8	0.000000	-10.6
01.12.2006	6.412444	-13.9	2.282183	-11.6	0.000000	-10.6
02.12.2006	1.832134	-4.4	1.521434	-5.4	1.665454	-16.0
03.12.2006	3.664282	-3.7	6.846357	-3.4	0.000000	-12.6
04.12.2006	3.664295	-1.3	4.564174	-1.1	0.000000	-9.9
05.12.2006	6.412541	0.4	3.803426	-0.4	0.000000	-5.8
06.12.2006	2.748242	-0.2	3.803373	-0.3	1.665578	-2.9
07.12.2006	7.328673	3.5	8.367303	2.2	0.000000	-2.9
08.12.2006	8.244789	2.7	5.324573	1.8	1.665641	-2.5
09.12.2006	4.580455	2.2	7.606426	2.0	0.000000	-2.4
10.12.2006	2.748284	2.8	4.563792	2.5	0.000000	-13.7
11.12.2006	3.664392	1.7	3.042486	1.7	1.665734	-9.1
12.12.2006	6.412710	0.5	5.324275	1.3	1.665765	-6.0
13.12.2006	5.496629	-0.3	3.042401	1.1	1.665797	-4.3
14.12.2006	6.412758	0.0	3.802948	-0.6	0.000000	-2.1
15.12.2006	8.245006	-4.6	3.042316	-7.0	0.000000	-4.1
16.12.2006	3.664461	-3.8	5.323978	-2.4	0.000000	-7.2
17.12.2006	4.580593	1.3	1.521115	0.4	0.000000	-1.9
18.12.2006	11.909588	0.9	2.281641	-0.3	1.665952	-0.9
19.12.2006	6.412879	-3.3	4.563219	-4.4	1.665984	-2.0
20.12.2006	4.580645	-7.7	6.084207	-8.7	3.332029	-4.1
21.12.2006	6.412927	-11.3	4.563091	-14.3	0.000000	-8.1
22.12.2006	10.993631	-14.3	3.042018	-14.5	1.666077	-4.0
23.12.2006	2.748418	-9.1	2.281482	-8.8	0.000000	-13.8
24.12.2006	3.664571	-8.7	5.323384	-7.9	0.000000	-18.1
25.12.2006	6.413024	-7.9	3.802364	-9.0	0.000000	-10.7
26.12.2006	6.413048	-15.3	2.281386	-12.4	0.000000	-6.1
27.12.2006	4.580766	-20.3	3.802258	-16.9	0.000000	-5.9
28.12.2006	1.832313	-14.2	3.802205	-14.7	0.000000	-4.8
29.12.2006	0.916160	-8.4	9.885594	-11.8	1.666295	-5.8
30.12.2006	4.580818	-12.7	5.322938	-15.1	0.000000	-8.6
31.12.2006	0.916167	-21.4	0.000000	-23.8	0.000000	-5.2
01.01.2007	2.748511	-14.9	2.281195	-16.7	0.000000	-11.1
02.01.2007	1.832336	-5.4	4.562316	-6.1	0.000000	-16.7
03.01.2007	1.832332	-0.5	3.041494	0.4	0.000000	-14.6
04.01.2007	7.329308	-2.7	3.801805	-0.4	0.000000	-8.6
05.01.2007	2.748484	-4.8	3.801742	-2.4	0.000000	-6.8

Data	Chelyabinsk		Yekaterinburg		Barnaul	
	RI rates	Temperature	RI rates	Temperature	RI rates	Temperature
06.01.2007	0.916159	-5.4	3.801679	-4.0	0.000000	-7.9
07.01.2007	5.496940	-8.7	7.603234	-3.1	0.000000	-9.8
08.01.2007	2.748463	-12.1	5.322176	-3.9	1.666543	-16.1
09.01.2007	7.329216	-12.1	1.520597	-8.6	1.666565	-15.3
10.01.2007	7.329198	-5.7	8.363144	-4.0	0.000000	-9.0
11.01.2007	6.413032	-2.6	7.602733	-0.8	0.000000	-9.2
12.01.2007	8.245306	1.1	5.321826	1.5	0.000000	-6.6
13.01.2007	2.748428	2.4	3.801242	1.6	0.000000	-3.5
14.01.2007	6.412984	-2.2	3.801179	-0.4	0.000000	-4.8
15.01.2007	8.245244	-4.1	9.122679	-2.0	0.000000	-10.7
16.01.2007	10.077495	-6.8	3.801054	-3.6	0.000000	-11.4
17.01.2007	6.412935	-5.1	9.122379	-4.8	0.000000	-11.8
18.01.2007	7.329051	-4.8	5.321300	-5.9	0.000000	-11.3
19.01.2007	7.329032	-3.1	7.601733	-2.7	0.000000	-5.7
20.01.2007	7.329014	0.4	5.321125	2.3	0.000000	-7.4
21.01.2007	7.328995	-3.1	7.601482	-1.9	0.000000	-9.7
22.01.2007	14.657954	-3.8	6.841222	-1.9	0.000000	-8.8
23.01.2007	12.825677	-3.8	7.601232	-2.7	8.334372	-10.1
24.01.2007	12.825645	-10.0	2.280332	-11.7	0.000000	-5.8
25.01.2007	8.245037	-15.7	9.881277	-15.6	1.666919	-7.3
26.01.2007	7.328903	-11.7	6.840771	-13.4	13.335526	-2.8
27.01.2007	10.077217	-12.6	8.360805	-14.9	0.000000	-3.7
28.01.2007	8.244975	-8.7	5.320425	-12.8	0.000000	-5.2
29.01.2007	17.406014	-4.8	5.320337	-7.5	1.667007	-2.9
30.01.2007	14.657660	-9.1	11.400535	-9.3	0.000000	-0.6
31.01.2007	15.573724	-12.1	3.040093	-10.6	1.667051	0.5
01.02.2007	11.909289	-10.6	6.840096	-11.0	0.000000	-2.9
02.02.2007	6.412678	-12.6	4.559989	-14.0	0.000000	-3.9
03.02.2007	3.664378	-17.3	6.079886	-18.7	0.000000	-8.0
04.02.2007	12.825291	-18.0	3.039893	-17.6	0.000000	-7.0
05.02.2007	6.412629	-15.2	4.559764	-12.8	0.000000	-8.3
06.02.2007	9.160876	-14.7	7.599482	-14.6	5.001551	-6.5
07.02.2007	8.244768	-9.5	6.079486	-8.9	1.667206	-2.4
08.02.2007	10.076913	-4.8	4.559539	-7.4	0.000000	-5.6
09.02.2007	5.496484	-8.5	3.039643	-11.0	0.000000	-6.6
10.02.2007	4.580392	-14.4	1.519796	-16.7	0.000000	-8.8
11.02.2007	1.832152	-4.4	2.279657	-9.1	0.000000	-13.9
12.02.2007	8.244664	-12.4	6.838859	-13.6	1.667316	-6.3
13.02.2007	10.076787	-13.2	4.559164	-14.4	1.667338	-7.0
14.02.2007	9.160692	-14.8	4.559089	-11.9	0.000000	-18.5
15.02.2007	6.412469	-7.6	4.559014	-6.8	0.000000	-19.8

Data	Chelyabinsk		Yekaterinburg		Barnaul	
	RI rates	Temperature	RI rates	Temperature	RI rates	Temperature
16.02.2007	6.412452	-14.8	6.078586	-18.6	0.000000	-13.3
17.02.2007	10.076686	-25.6	5.318675	-26.1	0.000000	-13.1
18.02.2007	1.832120	-26.9	6.078386	-26.3	0.000000	-13.3
19.02.2007	9.160577	-25.3	7.597857	-24.4	0.000000	-7.9
20.02.2007	10.076610	-21.3	4.558639	-21.7	1.667493	-13.5
21.02.2007	17.405010	-20.5	5.318325	-20.4	0.000000	-9.7
22.02.2007	3.664203	-19.1	0.759748	-21.3	0.000000	-7.6
23.02.2007	3.664194	-19.3	4.558415	-18.8	0.000000	-6.0
24.02.2007	10.076509	-17.1	2.279170	-18.5	0.000000	-15.4
25.02.2007	6.412308	-23.3	6.837397	-23.9	0.000000	-14.1
26.02.2007	4.580208	-21.8	4.558190	-21.7	0.000000	-12.1
27.02.2007	4.580197	-13.3	3.798429	-15.2	0.000000	-20.2
28.02.2007	5.496222	-10.8	1.519347	-12.6	0.000000	-20.6
01.03.2007	10.076382	-12.9	4.557965	-13.8	0.000000	-15.9
02.03.2007	9.160324	-13.2	1.519297	-9.6	1.667714	-16.9
03.03.2007	2.748090	-9.2	1.519272	-5.2	0.000000	-16.1
04.03.2007	3.664111	-7.7	6.076987	-6.8	0.000000	-15.4
05.03.2007	4.580128	-4.4	5.317276	-9.3	0.000000	-14.5
06.03.2007	6.412163	-4.7	3.797992	-9.1	0.000000	-11.6
07.03.2007	0.916021	-9.9	3.797929	-5.9	0.000000	-13.6
08.03.2007	0.916019	-5.9	3.038294	-0.2	0.000000	-14.7
09.03.2007	8.244147	-3.4	3.038244	-4.5	0.000000	-12.2
10.03.2007	3.664056	-5.1	6.076387	-8.9	0.000000	-7.7
11.03.2007	4.580059	-12.2	4.557216	-14.6	1.667913	-12.2
12.03.2007	5.496057	-14.9	9.114281	-11.3	0.000000	-14.8
13.03.2007	4.580036	-9.3	4.557066	-7.4	0.000000	-15.0
14.03.2007	6.412034	-7.3	4.556991	-4.6	1.667979	-13.4
15.03.2007	5.496015	-1.1	8.354346	-2.2	1.668001	-9.2
16.03.2007	3.664001	-2.1	5.316314	-0.1	1.668023	-8.7
17.03.2007	10.991976	-5.7	12.151376	-0.7	0.000000	-5.9
18.03.2007	2.747987	-3.1	7.594485	-1.4	0.000000	0.6
19.03.2007	5.495960	-3.0	5.316052	-4.1	1.668090	0.1
20.03.2007	5.495946	-2.2	4.556541	-1.4	1.668112	-0.4
21.03.2007	10.075876	2.8	5.315878	3.6	1.668134	-3.6
22.03.2007	9.159865	5.3	1.518797	5.2	0.000000	-2.2
23.03.2007	7.327873	4.3	3.796931	2.8	0.000000	0.5
24.03.2007	4.579909	2.7	1.518747	1.8	1.668200	-0.2
25.03.2007	2.747939	-1.9	3.037445	-2.4	0.000000	-1.7
26.03.2007	9.159773	-3.4	3.037395	-4.7	1.668244	1.3
27.03.2007	2.747925	-2.5	2.278009	-2.4	0.000000	2.8
28.03.2007	7.327781	2.5	3.796619	2.3	1.668289	-3.8

Data	Chelyabinsk		Yekaterinburg		Barnaul	
	RI rates	Temperature	RI rates	Temperature	RI rates	Temperature
29.03.2007	10.075674	3.4	6.074490	2.4	1.668311	-2.7
30.03.2007	3.663872	3.7	6.833689	3.7	0.000000	-5.2
31.03.2007	3.663863	5.0	3.796431	6.6	0.000000	-4.4
01.04.2007	3.663854	3.3	9.870559	3.2	0.000000	-0.9
02.04.2007	11.907495	1.7	7.592613	1.4	0.000000	2.1
03.04.2007	7.327671	5.0	8.351737	3.4	0.000000	1.2
04.04.2007	10.075522	6.9	6.073891	6.1	0.000000	1.4
05.04.2007	5.495726	6.8	9.110687	7.9	0.000000	3.7
06.04.2007	6.411664	3.1	3.796057	7.2	1.668488	9.3
07.04.2007	2.747849	6.8	3.795995	7.6	0.000000	10.2
08.04.2007	4.579737	5.1	9.110238	3.3	1.668532	6.6
09.04.2007	5.495671	4.9	4.555044	3.1	0.000000	7.2
10.04.2007	0.915943	1.3	6.832454	0.1	1.668576	10.8
11.04.2007	3.663762	3.8	3.795745	0.3	0.000000	8.6
12.04.2007	6.411567	6.3	4.554819	4.6	3.337241	3.1
13.04.2007	6.411551	3.4	3.795620	3.3	0.000000	4.5
14.04.2007	3.663734	5.6	3.036447	2.9	0.000000	7.9
15.04.2007	2.747794	5.2	4.554595	4.8	0.000000	7.1
16.04.2007	4.579645	5.4	6.831780	4.8	0.000000	11.7
17.04.2007	7.327414	4.2	5.313519	3.4	0.000000	14.7
18.04.2007	11.907017	5.7	5.313432	5.5	0.000000	4.2
19.04.2007	2.747766	7.3	1.518099	7.2	1.668775	4.5
20.04.2007	7.327359	6.9	11.385552	5.2	0.000000	7.4
21.04.2007	3.663670	7.9	6.072195	6.8	1.668819	11.3
22.04.2007	4.579576	10.8	2.277036	9.6	0.000000	13.2
23.04.2007	3.663652	10.8	6.071995	7.6	1.668864	15.4
24.04.2007	4.579553	11.1	15.938725	8.6	0.000000	17.7
25.04.2007	0.000000	4.2	1.517949	4.9	0.000000	17.4
26.04.2007	1.831812	5.7	6.830658	6.0	1.668930	19.5
27.04.2007	4.579519	9.2	4.553697	8.6	1.668952	16.1
28.04.2007	3.663606	14.5	4.553622	10.4	0.000000	10.9
29.04.2007	2.747697	10.2	3.794623	6.4	1.668996	14.1
30.04.2007	7.327175	4.6	3.035648	1.2	0.000000	14.1
01.05.2007	1.831789	4.9	0.758900	3.4	1.669041	16.7
02.05.2007	9.158922	6.7	7.588872	2.7	0.000000	19.4
03.05.2007	5.495340	5.6	3.794374	2.9	1.669085	16.0
04.05.2007	3.663551	6.3	3.035449	4.3	0.000000	18.8
05.05.2007	1.831771	6.6	3.035399	5.2	0.000000	7.3
06.05.2007	3.663532	5.9	2.276512	4.4	0.000000	11.8
07.05.2007	3.663523	5.2	3.794124	4.3	0.000000	14.2
08.05.2007	6.411149	8.9	3.035250	7.5	0.000000	10.3

Data	Chelyabinsk		Yekaterinburg		Barnaul	
	RI rates	Temperature	RI rates	Temperature	RI rates	Temperature
09.05.2007	1.831752	10.9	3.035200	8.9	0.000000	6.7
10.05.2007	3.663495	14.3	5.311512	13.2	0.000000	8.9
11.05.2007	1.831743	13.9	3.793875	10.7	1.669262	15.7
12.05.2007	2.747608	11.7	0.758763	7.3	0.000000	17.1
13.05.2007	0.000000	13.1	4.552501	10.1	0.000000	10.6
14.05.2007	3.663459	13.9	5.311164	7.3	1.669328	13.3
15.05.2007	5.495174	5.4	1.517450	2.6	3.338701	17.7
16.05.2007	1.831720	7.1	3.034851	8.7	0.000000	21.6
17.05.2007	0.915858	14.7	0.758700	15.8	1.669395	12.6
18.05.2007	1.831711	21.4	4.552127	18.6	0.000000	13.0
19.05.2007	0.915853	16.1	2.276026	15.0	0.000000	16.9
20.05.2007	2.747553	14.4	3.034652	13.7	0.000000	10.9
21.05.2007	1.831697	20.0	2.275951	19.8	1.669483	6.7
22.05.2007	3.663385	21.7	4.551828	20.5	1.669506	8.7
23.05.2007	3.663376	18.6	3.793128	16.1	0.000000	13.4
24.05.2007	0.915842	14.4	4.551678	12.8	0.000000	14.1
25.05.2007	3.663358	15.3	6.068805	14.4	0.000000	10.9
26.05.2007	3.663348	13.3	3.034353	14.4	0.000000	15.3
27.05.2007	4.579174	18.7	3.792878	18.4	0.000000	12.4
28.05.2007	2.747497	21.9	1.517126	22.6	0.000000	11.5
29.05.2007	4.579151	24.6	2.275652	23.9	0.000000	12.3
30.05.2007	6.410795	13.3	4.551230	9.9	0.000000	15.1
31.05.2007	2.747477	14.0	2.275578	13.2	0.000000	7.6
01.06.2007	5.494940	20.8	3.034054	17.1	0.000000	8.0
02.06.2007	3.663284	7.3	0.000000	4.8	0.000000	11.1
03.06.2007	2.747456	5.2	3.033954	3.5	0.000000	9.2
04.06.2007	0.915816	3.1	3.792380	2.2	1.669794	8.9
05.06.2007	1.831628	4.0	0.758464	4.0	0.000000	7.9
06.06.2007	0.000000	6.3	3.033805	6.5	0.000000	9.9
07.06.2007	1.831619	12.2	4.550632	9.1	1.669860	14.2
08.06.2007	4.579036	13.9	2.275279	11.1	0.000000	11.3
09.06.2007	0.915805	11.4	0.758414	9.9	0.000000	13.8
10.06.2007	0.000000	10.8	1.516803	10.5	0.000000	16.4
11.06.2007	0.915800	9.2	1.516778	7.3	0.000000	17.6
12.06.2007	0.915798	10.6	4.550259	9.2	0.000000	15.1
13.06.2007	3.663183	13.1	0.000000	12.2	0.000000	14.0
14.06.2007	0.915793	18.3	2.275055	15.1	0.000000	12.9
15.06.2007	1.831582	21.2	0.758339	18.2	0.000000	12.1
16.06.2007	5.494733	21.9	2.274980	19.2	0.000000	15.1
17.06.2007	1.831573	16.3	0.000000	13.6	0.000000	22.3
18.06.2007	2.747353	17.1	2.274905	16.6	0.000000	16.1

Data	Chelyabinsk		Yekaterinburg		Barnaul	
	RI rates	Temperature	RI rates	Temperature	RI rates	Temperature
19.06.2007	4.578910	24.1	0.758289	22.1	0.000000	19.4
20.06.2007	1.831559	23.7	2.274831	20.6	0.000000	20.5
21.06.2007	7.326219	25.9	1.516529	21.1	0.000000	22.9
22.06.2007	1.831550	23.3	0.758252	20.7	0.000000	23.7
23.06.2007	0.915773	16.1	0.758240	14.5	0.000000	24.9
24.06.2007	0.915770	14.2	1.516454	12.4	0.000000	22.9
25.06.2007	3.663073	16.1	2.274644	16.3	0.000000	16.0
26.06.2007	3.663063	19.8	0.000000	18.0	0.000000	17.1
27.06.2007	3.663054	19.4	0.000000	19.1	0.000000	19.8
28.06.2007	1.831523	19.8	0.758177	20.0	0.000000	21.3
29.06.2007	1.831518	20.9	0.000000	22.0	0.000000	18.0
30.06.2007	1.831513	21.4	1.516305	21.7	0.000000	17.8
01.07.2007	1.831509	22.7	0.758140	24.5	0.000000	18.3
02.07.2007	2.747256	23.6	0.758128	24.7	0.000000	20.3
03.07.2007	2.747249	23.6	1.516230	24.7	0.000000	21.3
04.07.2007	0.915747	20.3	0.758103	21.3	0.000000	21.2
05.07.2007	1.831490	18.8	0.758090	19.8	0.000000	21.4
06.07.2007	1.831486	20.6	0.758078	20.7	0.000000	22.3
07.07.2007	0.000000	22.0	0.000000	22.3	0.000000	25.0
08.07.2007	1.831477	22.7	2.274159	23.9	0.000000	23.9
09.07.2007	0.000000	20.8	0.758041	22.6	0.000000	24.6
10.07.2007	0.915734	21.0	1.516056	21.7	0.000000	21.1
11.07.2007	1.831463	17.8	0.758016	19.5	0.000000	21.2
12.07.2007	0.000000	20.1	0.758003	20.8	0.000000	23.2
13.07.2007	0.000000	21.2	0.757991	23.1	0.000000	21.4
14.07.2007	0.000000	20.1	3.789892	21.2	0.000000	22.6
15.07.2007	0.000000	21.9	0.000000	22.4	0.000000	21.6
16.07.2007	0.000000	23.1	0.757953	23.2	0.000000	21.7
17.07.2007	0.000000	23.1	0.757941	23.5	0.000000	20.8
18.07.2007	1.831431	19.3	0.757929	18.3	0.000000	22.9
19.07.2007	1.831426	19.5	0.000000	17.1	0.000000	24.6
20.07.2007	2.747132	18.1	3.031615	16.7	0.000000	25.8
21.07.2007	2.747125	16.6	0.000000	15.4	0.000000	22.5
22.07.2007	0.000000	14.3	0.757879	13.3	0.000000	21.3
23.07.2007	1.831408	12.3	0.757866	11.3	1.670880	22.9
24.07.2007	1.831403	12.0	0.757854	11.2	0.000000	24.2
25.07.2007	1.831398	12.0	0.757842	11.6	0.000000	20.7
26.07.2007	0.915697	15.6	2.273487	15.7	0.000000	19.1
27.07.2007	2.747084	18.6	1.515633	18.5	0.000000	17.9
28.07.2007	1.831385	19.1	0.757804	19.6	0.000000	17.8
29.07.2007	0.000000	19.6	1.515584	19.5	0.000000	18.4

Data	Chelyabinsk		Yekaterinburg		Barnaul	
	RI rates	Temperature	RI rates	Temperature	RI rates	Temperature
30.07.2007	0.915688	19.6	0.000000	19.9	1.671035	19.6
31.07.2007	0.000000	19.9	1.515534	19.0	0.000000	21.2
01.08.2007	0.915683	19.1	1.515509	19.8	0.000000	22.6
02.08.2007	1.831362	20.9	0.000000	20.9	0.000000	22.3
03.08.2007	0.915679	20.3	0.757730	20.0	0.000000	22.5
04.08.2007	0.915676	20.8	0.000000	19.1	0.000000	20.7
05.08.2007	2.747022	17.7	3.030819	17.8	0.000000	18.8
06.08.2007	2.747015	21.6	0.757692	21.2	0.000000	18.1
07.08.2007	0.915669	21.1	2.273040	20.6	0.000000	21.6
08.08.2007	0.915667	15.4	2.273003	14.3	0.000000	21.6
09.08.2007	2.746994	11.6	0.000000	11.6	0.000000	22.0
10.08.2007	1.831325	13.9	0.757643	14.3	0.000000	15.2
11.08.2007	1.831320	20.3	0.000000	19.6	0.000000	12.8
12.08.2007	0.000000	13.0	1.515236	11.7	0.000000	15.6
13.08.2007	1.831311	12.4	0.757605	12.0	0.000000	13.9
14.08.2007	0.915653	14.8	0.000000	12.9	0.000000	12.8
15.08.2007	0.915651	14.7	0.757581	14.8	0.000000	11.8
16.08.2007	1.831297	18.0	0.000000	19.3	0.000000	10.8
17.08.2007	2.746939	22.1	0.757556	22.4	0.000000	11.3
18.08.2007	0.000000	21.3	1.515087	21.2	0.000000	12.4
19.08.2007	0.915642	21.9	1.515062	22.9	0.000000	12.6
20.08.2007	0.915639	21.2	1.515037	22.3	0.000000	15.1
21.08.2007	4.578186	20.5	0.000000	18.8	0.000000	17.3
22.08.2007	2.746905	19.4	0.000000	18.1	0.000000	16.3
23.08.2007	2.746898	22.4	0.000000	20.6	0.000000	16.6
24.08.2007	1.831261	16.4	0.000000	14.5	0.000000	18.1
25.08.2007	0.915628	21.4	0.757456	19.1	0.000000	10.9
26.08.2007	1.831251	23.2	0.757444	19.9	0.000000	14.0
27.08.2007	0.915623	21.1	0.757432	19.5	0.000000	13.4
28.08.2007	5.493727	21.4	1.514838	19.8	0.000000	18.0
29.08.2007	0.915619	21.6	0.757407	20.1	0.000000	22.5
30.08.2007	2.746850	19.7	0.757394	16.9	0.000000	21.6
31.08.2007	2.746843	15.3	0.000000	13.1	0.000000	22.2
01.09.2007	2.746836	14.4	1.514739	12.3	0.000000	23.8
02.09.2007	2.746829	13.7	0.757357	13.7	0.000000	24.7
03.09.2007	0.915607	13.0	0.000000	11.8	0.000000	24.6
04.09.2007	2.746815	14.8	1.514665	12.9	0.000000	19.8
05.09.2007	2.746808	15.9	0.000000	14.7	0.000000	11.8
06.09.2007	0.000000	12.6	0.000000	10.5	0.000000	14.1
07.09.2007	1.831196	10.8	0.757295	9.3	0.000000	15.7
08.09.2007	2.746788	14.5	0.757283	11.6	0.000000	10.7

Data	Chelyabinsk		Yekaterinburg		Barnaul	
	RI rates	Temperature	RI rates	Temperature	RI rates	Temperature
09.09.2007	0.915594	20.3	0.757270	14.0	0.000000	12.3
10.09.2007	0.915591	18.5	1.514516	13.8	0.000000	18.2
11.09.2007	0.915589	15.8	0.000000	15.3	0.000000	20.9
12.09.2007	0.915587	10.1	2.271699	8.7	1.672012	22.7
13.09.2007	3.662337	6.7	2.271662	5.6	0.000000	15.8
14.09.2007	0.915582	8.8	1.514416	7.8	0.000000	10.2
15.09.2007	1.831160	11.8	0.000000	8.8	0.000000	8.7
16.09.2007	0.915577	11.9	0.757183	9.2	0.000000	9.7
17.09.2007	0.915575	12.4	2.271513	11.2	0.000000	13.1
18.09.2007	6.409010	12.3	0.757159	12.4	0.000000	14.1
19.09.2007	0.915571	11.0	0.757146	10.8	0.000000	12.6
20.09.2007	1.831137	7.7	0.757134	7.2	0.000000	14.2
21.09.2007	0.915566	5.9	1.514243	5.9	0.000000	7.7
22.09.2007	2.746691	10.2	0.000000	8.6	0.000000	6.0
23.09.2007	0.915561	8.8	0.757096	8.3	0.000000	4.9
24.09.2007	1.831118	12.3	0.000000	10.3	0.000000	5.9
25.09.2007	0.915557	8.6	3.028287	7.7	0.000000	8.9
26.09.2007	1.831109	9.6	0.000000	9.5	0.000000	10.5
27.09.2007	3.662209	11.9	0.757047	11.8	0.000000	6.3
28.09.2007	1.831100	12.8	1.514069	11.9	0.000000	7.9
29.09.2007	0.915548	12.3	0.757022	10.6	0.000000	10.8
30.09.2007	0.915545	7.6	0.757010	6.4	3.344823	12.3
01.10.2007	3.662172	4.6	1.513994	3.9	0.000000	12.6
02.10.2007	0.915541	2.9	0.000000	4.1	3.344912	11.9
03.10.2007	1.831077	6.7	2.270917	6.3	3.344957	0.6
04.10.2007	3.662145	7.5	0.756960	6.9	0.000000	1.7
05.10.2007	5.493203	8.7	0.756948	7.4	0.000000	4.2
06.10.2007	0.915532	10.2	0.756935	9.3	0.000000	5.6
07.10.2007	1.831059	8.6	0.756923	7.9	0.000000	7.3
08.10.2007	0.915527	8.2	3.027642	8.0	0.000000	2.7
09.10.2007	3.662099	9.1	1.513796	9.8	1.672612	-1.1
10.10.2007	2.746567	12.5	0.000000	12.6	0.000000	-0.2
11.10.2007	0.915520	7.6	0.756873	6.6	0.000000	1.8
12.10.2007	2.746553	6.4	0.000000	4.8	0.000000	7.1
13.10.2007	2.746546	2.9	1.513697	2.9	0.000000	10.9
14.10.2007	5.493079	4.9	2.270508	3.7	0.000000	6.8
15.10.2007	1.831022	8.9	0.000000	4.2	3.345490	4.6
16.10.2007	0.915509	12.2	0.756811	8.0	0.000000	0.8
17.10.2007	2.746519	5.3	0.000000	2.9	0.000000	5.9
18.10.2007	0.915504	2.8	3.027146	3.1	0.000000	11.9
19.10.2007	2.746505	7.6	3.027096	6.2	3.345668	6.8

Data	Chelyabinsk		Yekaterinburg		Barnaul	
	RI rates	Temperature	RI rates	Temperature	RI rates	Temperature
20.10.2007	0.915499	10.1	0.756762	7.2	0.000000	5.2
21.10.2007	1.830994	5.7	1.513499	3.5	0.000000	6.4
22.10.2007	1.830990	2.3	0.000000	0.5	0.000000	0.3
23.10.2007	2.746478	2.8	2.270173	1.6	0.000000	-1.8
24.10.2007	3.661961	1.6	1.513424	0.9	0.000000	-0.6
25.10.2007	3.661952	1.2	2.270099	1.5	0.000000	-1.9
26.10.2007	4.577428	3.9	3.783437	3.4	0.000000	-2.6
27.10.2007	0.915483	5.4	0.000000	4.7	0.000000	-0.2
28.10.2007	0.915481	4.3	0.756663	3.7	0.000000	-2.4
29.10.2007	1.830957	2.6	2.269950	2.3	0.000000	-1.6
30.10.2007	2.746429	0.8	0.000000	0.5	0.000000	1.1
31.10.2007	4.577371	1.4	2.269876	0.3	0.000000	1.5
01.11.2007	3.661887	2.8	1.513226	2.0	0.000000	0.2
02.11.2007	2.746409	5.6	2.269802	5.9	0.000000	-0.8
03.11.2007	0.915467	5.1	2.269765	5.8	0.000000	-1.4
04.11.2007	1.830930	2.9	1.513152	2.7	0.000000	-0.6
05.11.2007	2.746388	1.1	4.539380	-1.1	0.000000	3.9
06.11.2007	3.661841	-4.3	0.756551	-6.3	0.000000	0.7
07.11.2007	2.746374	-9.8	0.000000	-10.7	1.673257	3.4
08.11.2007	2.746367	-10.1	1.513052	-9.1	0.000000	3.9
09.11.2007	0.000000	-8.5	3.782569	-9.1	0.000000	-7.9
10.11.2007	0.915451	-9.3	4.539009	-9.3	0.000000	-13.2
11.11.2007	0.915449	-12.4	1.512978	-11.0	1.673346	-13.1
12.11.2007	2.746340	-12.9	4.538860	-12.2	0.000000	-10.7
13.11.2007	6.408110	-10.5	2.269393	-11.4	0.000000	-12.0
14.11.2007	1.830884	-10.2	1.512904	-9.1	1.673412	-9.9
15.11.2007	4.577199	-6.9	0.756440	-6.9	0.000000	-6.3
16.11.2007	2.746312	-5.2	2.269281	-4.6	1.673457	-5.1
17.11.2007	0.915435	-2.0	3.025659	-2.1	1.673479	-1.4
18.11.2007	1.830866	1.1	3.025610	-1.7	1.673501	-1.0
19.11.2007	2.746292	-6.9	4.538340	-9.0	0.000000	-0.8
20.11.2007	2.746285	-10.3	1.512755	-12.9	0.000000	-3.2
21.11.2007	3.661704	-8.0	2.269096	-12.7	0.000000	-5.0
22.11.2007	9.154236	-13.6	0.000000	-13.6	0.000000	-1.8
23.11.2007	2.746264	-3.1	3.781702	-4.6	0.000000	-15.9
24.11.2007	3.661676	-1.1	0.756328	-3.9	1.673635	-9.6
25.11.2007	4.577084	-5.9	1.512631	-7.3	1.673657	-4.9
26.11.2007	2.746243	-7.9	3.025213	-4.5	1.673679	-9.2
27.11.2007	3.661649	-7.3	3.025164	-5.3	0.000000	-10.4
28.11.2007	4.577049	-12.1	3.781393	-11.5	0.000000	-12.9
29.11.2007	2.746223	-10.1	4.537597	-11.5	0.000000	-9.2

Data	Chelyabinsk		Yekaterinburg		Barnaul	
	RI rates	Temperature	RI rates	Temperature	RI rates	Temperature
30.11.2007	3.661621	-11.8	1.512508	-13.9	0.000000	-7.3
01.12.2007	1.830806	-5.3	1.512483	-8.2	0.000000	-5.7
02.12.2007	3.661603	-4.3	3.781145	-7.8	0.000000	-9.6
03.12.2007	4.576992	-14.4	3.781083	-15.5	0.000000	-7.1
04.12.2007	2.746188	-16.3	5.293430	-18.5	0.000000	-6.6
05.12.2007	2.746181	-14.8	3.780960	-11.9	3.347759	-6.8
06.12.2007	2.746174	-6.4	3.780898	-4.9	0.000000	-6.8
07.12.2007	1.830778	-4.0	4.537003	-5.5	3.347848	-10.4
08.12.2007	2.746161	-5.6	5.293084	-7.9	0.000000	-15.6
09.12.2007	2.746154	-11.7	1.512285	-10.8	0.000000	-16.9
10.12.2007	5.492294	-11.3	3.024520	-9.6	0.000000	-15.2
11.12.2007	3.661520	-9.9	2.268353	-10.8	0.000000	-15.5
12.12.2007	7.323022	-11.1	0.756105	-11.3	1.674036	-12.1
13.12.2007	5.492253	-9.8	6.804836	-9.9	1.674058	-9.8
14.12.2007	4.576866	-7.1	2.268242	-8.4	0.000000	-11.7
15.12.2007	4.576854	-6.7	2.268205	-7.7	0.000000	-12.2
16.12.2007	1.830737	-9.0	0.000000	-9.5	1.674125	-9.9
17.12.2007	2.746099	-7.7	3.780217	-8.6	0.000000	-10.0
18.12.2007	4.576820	-6.4	3.780155	-5.9	1.674169	-11.2
19.12.2007	3.661447	-8.7	5.292131	-7.7	5.022574	-6.9
20.12.2007	4.576797	-15.8	7.560063	-18.4	1.674214	-5.3
21.12.2007	4.576785	-13.6	5.291958	-15.7	1.674236	-6.2
22.12.2007	10.068902	-16.8	0.000000	-18.4	0.000000	-2.7
23.12.2007	4.576762	-20.1	0.000000	-19.5	1.674280	-5.2
24.12.2007	10.984202	-22.8	5.291698	-24.6	1.674303	-4.2
25.12.2007	2.746044	-22.1	3.779722	-22.9	3.348650	-16.8
26.12.2007	8.238110	-8.5	3.779660	-8.2	0.000000	-23.4
27.12.2007	7.322746	-7.6	3.023679	-6.8	0.000000	-16.4
28.12.2007	3.661364	-5.1	3.779537	-7.6	0.000000	-13.6
29.12.2007	9.153387	-7.7	2.267685	-9.6	0.000000	-13.7
30.12.2007	3.661346	-7.7	3.779413	-8.6	0.000000	-15.8
31.12.2007	4.576671	-5.3	0.000000	-8.4	0.000000	-16.9
01.01.2008	7.322654	-8.7	2.267574	-11.1	0.000000	-11.4
02.01.2008	2.745987	-16.3	2.267530	-17.2	0.000000	-13.3
03.01.2008	7.322611	-18.9	3.023316	-17.1	0.000000	-23.8
04.01.2008	6.407265	-10.9	3.779073	-11.1	0.000000	-32.4
05.01.2008	0.915321	-12.7	3.023200	-14.1	0.000000	-25.8
06.01.2008	3.661272	-7.9	3.023142	-11.1	0.000000	-17.6
07.01.2008	5.491892	-8.9	6.801940	-13.6	0.000000	-17.8
08.01.2008	9.153126	-15.3	2.267270	-18.4	6.697903	-21.9
09.01.2008	8.237789	-21.8	2.267227	-20.7	8.372375	-18.3

Data	Chelyabinsk		Yekaterinburg		Barnaul	
	RI rates	Temperature	RI rates	Temperature	RI rates	Temperature
10.01.2008	10.983685	-18.8	0.755728	-20.5	3.348949	-17.0
11.01.2008	2.745913	-21.8	7.557133	-23.3	5.023421	-25.2
12.01.2008	5.491810	-25.6	1.511398	-23.3	1.674473	-24.1
13.01.2008	6.407092	-21.2	1.511369	-14.4	1.674472	-23.3
14.01.2008	9.152961	-19.5	3.778350	-14.4	5.023414	-20.4
15.01.2008	8.237640	-22.0	2.266966	-19.7	1.674471	-16.2
16.01.2008	10.068197	-27.8	5.289487	-22.9	1.674470	-20.8
17.01.2008	4.576439	-14.9	9.823146	-10.3	15.070223	-30.2
18.01.2008	11.898707	-14.0	6.800509	-9.7	10.046811	-30.0
19.01.2008	10.068106	-16.5	6.044782	-11.7	1.674468	-22.8
20.01.2008	6.406958	-15.1	6.044666	-12.9	0.000000	-15.9
21.01.2008	13.729154	-11.6	6.800119	-11.5	1.674466	-13.9
22.01.2008	9.152742	-17.1	4.533326	-13.9	10.046793	-13.7
23.01.2008	3.661086	-15.6	6.044319	-11.2	3.348930	-13.3
24.01.2008	9.152687	-7.9	6.044204	-4.7	8.372320	-15.2
25.01.2008	10.067925	-10.2	5.288577	-9.4	11.721243	-16.1
26.01.2008	9.152632	-12.6	3.021986	-12.9	6.697850	-20.9
27.01.2008	7.322084	-18.1	5.288375	-14.3	3.348924	-21.2
28.01.2008	6.406804	-16.4	3.021871	-13.9	18.419072	-20.9
29.01.2008	12.813569	-10.1	3.777266	-7.7	8.372302	-21.6
30.01.2008	5.491513	-11.9	6.798949	-11.4	20.093515	-21.0
31.01.2008	9.152495	-10.3	7.554243	-11.8	5.023377	-17.9
01.02.2008	4.576234	-15.3	6.798689	-11.8	6.697833	-16.7
02.02.2008	2.745732	-13.9	6.043163	-11.1	3.348915	-18.3
03.02.2008	2.745724	-16.7	3.021524	-13.2	1.674457	-17.8
04.02.2008	4.576192	-9.6	4.532199	-6.6	1.674456	-18.8
05.02.2008	6.406650	-6.4	1.510704	-5.6	0.000000	-18.8
06.02.2008	6.406631	-6.1	3.776688	-7.4	6.697818	-13.8
07.02.2008	5.491382	-19.3	4.531939	-19.7	3.348907	-11.5
08.02.2008	9.152275	-22.7	3.776544	-24.7	10.046718	-15.3
09.02.2008	3.660899	-14.2	5.287060	-13.2	0.000000	-18.2
10.02.2008	4.576110	-6.1	4.531679	-10.7	0.000000	-20.2
11.02.2008	3.660877	-10.5	8.307920	-10.9	8.372254	-13.8
12.02.2008	7.321732	-8.1	7.552510	-8.3	5.023350	-14.7
13.02.2008	2.745641	-4.8	10.573312	-3.4	15.070044	-17.0
14.02.2008	5.491266	-4.8	6.041777	-4.1	15.070037	-17.1
15.02.2008	5.491250	-8.4	6.796869	-9.4	10.046687	-16.7
16.02.2008	3.660822	-12.2	12.083092	-12.7	6.697788	-13.5
17.02.2008	5.491217	-14.8	12.082861	-17.6	10.046678	-12.2
18.02.2008	8.236801	-13.0	16.613617	-17.2	6.697783	-10.0
19.02.2008	8.236776	-13.8	6.796350	-11.5	1.674445	-17.8

Data	Chelyabinsk		Yekaterinburg		Barnaul	
	RI rates	Temperature	RI rates	Temperature	RI rates	Temperature
20.02.2008	7.321557	-14.4	6.041084	-14.2	6.697777	-15.8
21.02.2008	3.660767	-11.6	5.285848	-12.7	13.395547	-6.5
22.02.2008	8.236702	-11.6	5.285747	-11.8	3.348885	-4.7
23.02.2008	2.745559	-5.0	4.530553	-4.2	5.023326	-9.6
24.02.2008	3.660734	2.4	9.060934	0.9	3.348882	-6.6
25.02.2008	7.321447	-6.5	5.285444	-9.9	6.697762	-5.3
26.02.2008	10.066959	-0.2	5.285343	-2.6	6.697759	-4.4
27.02.2008	4.575877	-1.9	9.815449	-6.0	3.348878	-0.1
28.02.2008	13.727590	-0.7	4.530121	-2.8	5.023315	-2.8
29.02.2008	7.321359	3.1	9.060068	1.6	15.069938	-2.8
01.03.2008	5.491003	-0.4	4.529947	-0.9	10.046621	-2.3
02.03.2008	4.575822	-0.2	5.284838	-0.4	5.023308	-2.9
03.03.2008	4.575808	-3.3	9.814511	-0.2	5.023306	-5.7
04.03.2008	9.151589	-0.9	5.284636	0.3	3.348869	-5.8
05.03.2008	9.151562	0.8	4.529601	0.1	6.697735	-3.1
06.03.2008	10.066688	-1.6	5.284434	-2.5	5.023299	-0.1
07.03.2008	5.490904	-2.5	4.529428	-2.8	0.000000	-1.9
08.03.2008	5.490888	-2.2	8.303793	-1.9	3.348863	-0.3
09.03.2008	5.490871	-2.3	6.039007	-3.3	1.674431	0.7
10.03.2008	3.660570	-5.2	6.038891	-7.9	5.023291	-3.4
11.03.2008	8.236257	-7.4	7.548470	-8.4	0.000000	-8.9
12.03.2008	9.151370	-4.8	5.283828	-5.0	8.372144	-5.3
13.03.2008	5.490805	-0.8	7.548181	-1.4	8.372140	-3.4
14.03.2008	4.575657	0.8	9.812448	0.1	5.023282	-2.6
15.03.2008	3.660515	-4.3	6.038314	-7.5	0.000000	-2.1
16.03.2008	3.660504	-11.2	10.566848	-13.4	3.348852	-13.2
17.03.2008	6.405863	-10.5	9.811886	-11.4	11.720975	-5.2
18.03.2008	8.236085	-7.1	10.566445	-9.1	5.023273	1.9
19.03.2008	5.490707	-2.8	6.037853	-4.6	0.000000	-0.8
20.03.2008	5.490690	0.4	6.037738	-0.3	8.372114	-2.3
21.03.2008	10.981347	0.8	7.547028	0.6	8.372111	1.7
22.03.2008	7.320876	-1.8	6.792195	-0.9	0.000000	4.9
23.03.2008	5.490641	-2.6	3.018696	-3.8	1.674421	9.0
24.03.2008	3.660416	-0.6	9.810574	-0.1	10.046519	-0.2
25.03.2008	16.471824	2.5	6.037161	4.8	8.372096	-1.6
26.03.2008	8.235887	0.9	6.037046	4.6	6.697674	-0.8
27.03.2008	12.811342	1.3	10.564628	4.3	0.000000	1.3
28.03.2008	6.405652	2.4	5.282213	4.3	5.023251	3.7
29.03.2008	2.745271	3.7	5.282112	4.6	3.348832	2.6
30.03.2008	8.235788	4.3	6.791158	4.3	0.000000	4.1
31.03.2008	10.065933	5.4	9.054704	4.0	3.348830	3.2

Data	Chelyabinsk		Yekaterinburg		Barnaul	
	RI rates	Temperature	RI rates	Temperature	RI rates	Temperature
01.04.2008	7.320657	5.7	6.036354	2.7	1.674414	3.8
02.04.2008	10.065873	3.2	6.036239	4.8	5.023240	4.8
03.04.2008	3.660306	6.4	8.299670	5.7	3.348825	5.3
04.04.2008	2.745222	6.4	7.545010	4.3	6.697647	6.3
05.04.2008	4.575356	-2.9	6.035893	-4.5	0.000000	2.8
06.04.2008	1.830137	-0.2	3.017889	0.1	1.674410	-7.2
07.04.2008	1.830131	8.7	9.053493	7.1	5.023229	-3.4
08.04.2008	19.216321	11.2	6.789990	6.7	10.046453	-0.7
09.04.2008	10.065662	13.8	8.298719	11.2	3.348816	-5.8
10.04.2008	4.575287	15.4	7.544146	12.3	5.023222	0.1
11.04.2008	9.150547	15.8	5.280801	13.1	5.023220	9.6
12.04.2008	10.065571	16.7	7.543858	14.4	11.720841	13.1
13.04.2008	4.575246	9.1	7.543713	2.9	1.674405	14.6
14.04.2008	6.405325	-5.0	4.526142	-5.9	1.674404	11.5
15.04.2008	12.810612	-5.9	4.526055	-6.0	3.348807	-2.7
16.04.2008	8.235369	-4.3	11.314922	-4.6	3.348806	-2.9
17.04.2008	5.490229	2.9	5.280196	2.1	3.348805	-7.1
18.04.2008	5.490213	8.3	8.297293	6.4	3.348803	-8.6
19.04.2008	6.405229	10.8	7.542849	9.0	8.372004	-0.3
20.04.2008	3.660120	10.9	9.051246	10.4	1.674400	5.3
21.04.2008	12.810381	12.6	8.296817	9.9	1.674399	8.3
22.04.2008	6.405172	10.8	9.050901	10.0	3.348797	12.3
23.04.2008	2.745065	12.8	9.050728	10.3	5.023194	16.7
24.04.2008	5.490114	2.9	3.771065	4.2	6.697588	16.6
25.04.2008	9.150163	5.8	6.787787	5.3	5.023189	15.7
26.04.2008	2.745041	8.4	6.787657	4.2	3.348791	7.1
27.04.2008	7.320086	2.4	3.770849	1.3	0.000000	9.3
28.04.2008	8.235072	3.8	11.312330	1.9	3.348788	1.9
29.04.2008	6.405037	4.2	5.278987	1.9	6.697574	3.2
30.04.2008	10.065028	4.9	9.049519	3.0	6.697571	0.7
01.05.2008	5.489999	6.0	5.278785	3.7	1.674392	2.4
02.05.2008	9.149971	1.3	5.278684	0.9	1.674391	3.8
03.05.2008	7.319955	0.6	5.278584	0.2	1.674390	5.0
04.05.2008	5.489950	6.6	8.294759	6.6	3.348779	5.7
05.05.2008	8.234900	13.3	8.294600	12.2	8.371945	6.8
06.05.2008	6.404903	13.3	5.278281	12.9	5.023165	9.9
07.05.2008	6.404884	11.1	5.278181	13.1	5.023163	14.4
08.05.2008	7.319845	14.3	6.032091	15.5	0.000000	16.9
09.05.2008	3.659912	8.4	4.523982	8.5	3.348772	21.4
10.05.2008	5.489851	12.9	3.769913	11.2	0.000000	22.2
11.05.2008	1.829945	15.9	5.277778	13.9	3.348769	15.2

**Data**	**Chelyabinsk**		**Yekaterinburg**		**Barnaul**	
	RI rates	Temperature	RI rates	Temperature	RI rates	Temperature
12.05.2008	1.829939	16.1	5.277677	15.6	1.674384	15.6
13.05.2008	8.234702	16.3	6.785455	16.2	1.674383	18.8
14.05.2008	6.404749	18.8	3.015700	18.3	8.371912	19.4
15.05.2008	3.659846	14.8	3.769553	12.1	3.348763	19.8
16.05.2008	6.404711	11.7	1.507793	7.8	5.023143	22.8
17.05.2008	1.829912	16.1	1.507764	10.7	0.000000	16.9
18.05.2008	3.659813	15.1	4.523205	11.7	0.000000	17.1
19.05.2008	3.659802	17.8	3.015413	17.1	1.674379	18.2
20.05.2008	0.914948	22.6	1.507678	20.9	0.000000	13.1
21.05.2008	4.574725	21.3	3.769122	17.4	6.697509	8.3
22.05.2008	4.574711	17.4	3.015240	13.1	11.720636	12.6
23.05.2008	3.659758	8.9	5.276569	6.9	1.674376	14.4
24.05.2008	2.744810	11.8	2.261344	9.3	3.348750	6.2
25.05.2008	0.914934	8.9	4.522601	8.2	3.348749	4.8
26.05.2008	5.489588	9.4	2.261257	6.7	0.000000	7.2
27.05.2008	5.489571	14.2	4.522429	8.2	3.348746	12.6
28.05.2008	1.829852	8.9	5.276066	7.6	3.348744	19.3
29.05.2008	3.659692	9.4	4.522256	8.3	0.000000	20.3
30.05.2008	0.914920	14.3	3.014780	12.0	0.000000	20.4
31.05.2008	2.744753	11.9	1.507361	10.7	0.000000	26.1
01.06.2008	0.914915	13.6	0.753666	12.2	0.000000	22.3
02.06.2008	1.829824	14.2	5.275563	11.6	0.000000	20.6
03.06.2008	0.914909	14.1	0.000000	11.4	1.674368	22.7
04.06.2008	6.404346	14.8	2.260869	14.0	0.000000	19.5
05.06.2008	4.574519	16.2	2.260826	14.7	1.674366	25.6
06.06.2008	3.659604	11.2	0.753594	7.7	0.000000	15.0
07.06.2008	3.659593	7.7	1.507160	7.4	0.000000	20.1
08.06.2008	4.574478	7.0	0.753566	5.8	0.000000	22.6
09.06.2008	0.914893	5.7	2.260654	5.8	1.674363	18.8
10.06.2008	2.744670	10.2	2.260610	10.1	0.000000	11.4
11.06.2008	0.914887	13.3	3.014090	12.7	0.000000	13.9
12.06.2008	0.000000	18.7	1.507016	16.1	0.000000	14.5
13.06.2008	4.574409	22.7	0.753494	20.9	1.674360	14.6
14.06.2008	3.659517	22.2	1.506959	19.8	0.000000	15.3
15.06.2008	3.659506	18.4	3.767325	19.1	0.000000	17.6
16.06.2008	9.148737	17.3	4.520704	17.8	0.000000	17.0
17.06.2008	4.574355	21.1	0.753436	21.7	0.000000	11.6
18.06.2008	6.404077	23.1	1.506844	23.1	0.000000	13.3
19.06.2008	5.489193	24.4	2.260223	23.9	0.000000	15.7
20.06.2008	2.744588	26.2	1.506786	23.7	0.000000	19.7
21.06.2008	1.829720	21.8	0.000000	21.3	1.674354	21.9

Data	Chelyabinsk		Yekaterinburg		Barnaul	
	RI rates	Temperature	RI rates	Temperature	RI rates	Temperature
22.06.2008	2.744572	20.1	1.506729	19.0	0.000000	22.2
23.06.2008	5.489127	20.8	2.260050	20.1	0.000000	18.9
24.06.2008	2.744555	21.4	1.506671	21.1	0.000000	17.8
25.06.2008	3.659396	20.9	3.013285	21.5	0.000000	17.2
26.06.2008	5.489078	18.9	0.753307	21.2	0.000000	19.3
27.06.2008	3.659374	19.1	1.506585	20.3	0.000000	22.9
28.06.2008	5.489045	15.6	1.506556	18.4	0.000000	20.7
29.06.2008	2.744514	17.8	0.753264	19.9	0.000000	18.7
30.06.2008	0.914835	16.9	1.506499	15.8	0.000000	19.3
01.07.2008	4.574163	15.7	3.766176	13.2	0.000000	20.3
02.07.2008	5.488979	17.7	0.000000	16.8	0.000000	21.9
03.07.2008	3.659308	16.4	2.259619	16.0	3.348691	24.0
04.07.2008	2.744473	13.4	2.259576	14.3	1.674345	20.6
05.07.2008	1.829643	15.6	0.753178	15.7	0.000000	16.3
06.07.2008	1.829638	17.9	2.259490	18.1	0.000000	16.7
07.07.2008	0.000000	21.1	1.506298	21.3	0.000000	15.4
08.07.2008	1.829627	20.1	1.506269	21.2	0.000000	16.9
09.07.2008	4.574053	21.8	0.000000	22.0	0.000000	18.9
10.07.2008	1.829616	25.2	1.506212	24.6	0.000000	22.7
11.07.2008	3.659221	24.5	0.753092	24.2	0.000000	22.5
12.07.2008	1.829605	23.7	0.000000	24.7	0.000000	20.2
13.07.2008	1.829599	21.6	1.506126	23.6	0.000000	21.6
14.07.2008	2.744391	21.3	3.765242	21.1	1.674338	23.3
15.07.2008	6.403559	21.7	3.012137	23.6	0.000000	24.7
16.07.2008	1.829583	23.2	1.506040	23.8	0.000000	24.8
17.07.2008	6.403521	24.3	1.506011	24.2	0.000000	23.9
18.07.2008	3.659144	20.1	3.764955	20.7	1.674335	25.0
19.07.2008	0.914783	19.9	1.505953	21.5	0.000000	21.3
20.07.2008	0.914780	19.9	3.764812	21.2	0.000000	21.4
21.07.2008	0.914778	22.0	2.258844	22.6	0.000000	20.4
22.07.2008	0.914775	22.2	1.505867	23.1	0.000000	20.8
23.07.2008	2.744317	23.0	0.000000	24.5	0.000000	17.2
24.07.2008	1.829539	24.5	2.258715	24.5	0.000000	15.4
25.07.2008	1.829533	23.3	3.011563	24.2	0.000000	17.8
26.07.2008	0.000000	18.4	1.505753	19.2	0.000000	21.1
27.07.2008	1.829523	19.4	1.505724	20.1	0.000000	25.2
28.07.2008	2.744276	19.7	1.505695	18.2	0.000000	26.0
29.07.2008	8.232802	19.7	0.752833	18.3	0.000000	23.6
30.07.2008	0.914753	18.9	2.258457	17.6	0.000000	25.7
31.07.2008	1.829501	17.9	0.000000	14.9	0.000000	25.2
01.08.2008	0.914748	15.7	1.505580	16.7	0.000000	21.7

Data	Chelyabinsk		Yekaterinburg		Barnaul	
	RI rates	Temperature	RI rates	Temperature	RI rates	Temperature
02.08.2008	0.914745	16.8	0.752776	15.3	0.000000	17.8
03.08.2008	0.914742	16.9	0.752762	15.2	0.000000	20.0
04.08.2008	1.829479	14.4	0.000000	12.2	0.000000	16.7
05.08.2008	2.744210	15.5	2.258199	10.8	0.000000	20.4
06.08.2008	0.914734	15.5	1.505437	11.2	0.000000	21.3
07.08.2008	1.829462	18.6	2.258112	15.5	0.000000	17.4
08.08.2008	0.914728	14.8	0.000000	12.8	0.000000	20.1
09.08.2008	0.914726	13.2	0.752675	12.7	0.000000	26.0
10.08.2008	3.658892	16.4	0.752661	16.3	0.000000	19.5
11.08.2008	1.829440	20.3	1.505294	18.3	0.000000	15.8
12.08.2008	1.829435	21.6	0.752632	20.1	0.000000	19.8
13.08.2008	0.000000	21.7	0.000000	21.2	0.000000	20.4
14.08.2008	2.744136	18.9	0.752604	17.9	0.000000	20.5
15.08.2008	3.658837	22.4	1.505179	21.7	0.000000	20.8
16.08.2008	0.000000	20.2	3.762876	20.4	0.000000	16.6
17.08.2008	0.914704	18.0	4.515365	17.4	1.674313	15.3
18.08.2008	2.744103	18.0	2.257639	19.6	0.000000	14.9
19.08.2008	5.488189	18.4	0.752532	16.9	0.000000	15.3
20.08.2008	5.488173	17.6	0.000000	14.7	0.000000	12.0
21.08.2008	2.744078	18.7	1.505007	16.3	0.000000	11.1
22.08.2008	4.573450	14.4	0.752489	14.4	1.674309	9.8
23.08.2008	1.829375	15.8	1.504950	13.8	0.000000	9.9
24.08.2008	0.914685	15.3	1.504921	14.5	0.000000	12.2
25.08.2008	4.573409	15.4	3.762231	16.5	0.000000	14.6
26.08.2008	1.829358	18.9	0.752432	19.1	0.000000	9.3
27.08.2008	6.402734	19.4	5.266922	17.9	0.000000	13.1
28.08.2008	2.744021	15.9	1.504806	14.7	0.000000	18.8
29.08.2008	3.658683	13.4	1.504778	12.6	0.000000	20.9
30.08.2008	1.829336	17.8	2.257123	16.3	0.000000	20.7
31.08.2008	3.658661	23.1	2.257080	20.1	0.000000	17.7
01.09.2008	2.743988	13.8	1.504692	11.6	0.000000	17.8
02.09.2008	1.829320	9.2	2.256994	8.2	0.000000	17.9
03.09.2008	3.658628	7.1	3.009269	5.1	1.674300	12.9
04.09.2008	0.914654	7.6	3.009211	7.2	0.000000	12.9
05.09.2008	0.914652	14.1	1.504577	12.3	0.000000	13.1
06.09.2008	2.743947	18.6	4.513645	16.5	0.000000	11.7
07.09.2008	0.000000	19.6	0.000000	16.3	0.000000	11.6
08.09.2008	1.829287	18.3	3.761228	12.9	0.000000	14.2
09.09.2008	4.573203	14.4	1.504462	11.9	0.000000	12.7
10.09.2008	0.914638	7.6	1.504434	7.3	0.000000	15.3
11.09.2008	1.829270	9.1	0.000000	8.2	0.000000	11.5

Data	Chelyabinsk		Yekaterinburg		Barnaul	
	RI rates	Temperature	RI rates	Temperature	RI rates	Temperature
12.09.2008	2.743897	9.7	0.752188	9.2	0.000000	9.2
13.09.2008	0.914630	8.4	0.000000	7.0	0.000000	8.7
14.09.2008	1.829254	8.2	1.504319	6.9	0.000000	10.5
15.09.2008	0.000000	6.8	3.008581	5.4	0.000000	8.3
16.09.2008	1.829243	3.5	0.752131	4.8	1.674290	5.8
17.09.2008	3.658475	5.4	0.752117	5.9	0.000000	5.5
18.09.2008	0.914616	6.2	0.752102	6.4	1.674289	4.2
19.09.2008	5.487680	5.9	4.512528	4.9	0.000000	3.1
20.09.2008	1.829221	4.8	5.264516	4.8	1.674288	4.9
21.09.2008	1.829216	4.9	3.760297	5.3	0.000000	5.9
22.09.2008	1.829210	5.4	3.760225	6.6	0.000000	5.9
23.09.2008	1.829205	7.6	3.008123	7.4	1.674285	5.0
24.09.2008	0.000000	8.6	4.512099	6.5	3.348569	5.1
25.09.2008	2.743790	9.2	1.504004	6.8	0.000000	5.9
26.09.2008	0.914594	6.6	3.759939	4.9	0.000000	4.6
27.09.2008	1.829183	1.3	1.503947	1.0	0.000000	7.5
28.09.2008	1.829177	1.4	0.751959	1.1	0.000000	8.2
29.09.2008	0.914586	4.7	1.503890	3.9	0.000000	2.6
30.09.2008	0.914583	9.4	2.255792	8.3	0.000000	3.1
01.10.2008	2.743741	9.1	3.759581	9.8	0.000000	4.7
02.10.2008	0.914578	9.1	0.000000	8.7	0.000000	6.9
03.10.2008	1.829150	9.6	2.255663	9.8	0.000000	10.1
04.10.2008	3.658289	12.4	1.503747	12.4	0.000000	10.7
05.10.2008	0.000000	13.9	0.751859	13.1	0.000000	11.3
06.10.2008	3.658267	12.8	2.255534	11.2	0.000000	8.7
07.10.2008	4.572820	11.1	0.751830	10.5	0.000000	10.0
08.10.2008	2.743684	12.2	2.255448	10.0	0.000000	3.8
09.10.2008	4.572792	13.4	3.007207	9.7	0.000000	6.8
10.10.2008	5.487334	3.3	0.751787	3.7	1.674273	10.5
11.10.2008	1.829106	1.9	4.510639	1.5	0.000000	15.5
12.10.2008	1.829100	2.2	0.751759	1.0	0.000000	4.1
13.10.2008	3.658190	6.8	0.751745	4.8	0.000000	3.3
14.10.2008	3.658179	5.5	0.000000	4.3	1.674270	7.3
15.10.2008	3.658168	5.8	0.751716	3.6	0.000000	7.2
16.10.2008	1.829079	6.5	1.503403	4.3	0.000000	5.7
17.10.2008	0.000000	0.9	0.751687	0.7	0.000000	5.9
18.10.2008	1.829068	0.4	3.006692	-0.1	0.000000	1.8
19.10.2008	0.914531	2.9	3.006635	3.3	0.000000	-0.8
20.10.2008	1.829057	2.7	1.503289	3.7	0.000000	-3.4
21.10.2008	1.829051	4.9	2.254890	3.7	1.674265	-1.6
22.10.2008	2.743569	4.1	0.751616	3.6	1.674264	2.4

Data	Chelyabinsk		Yekaterinburg		Barnaul	
	RI rates	Temperature	RI rates	Temperature	RI rates	Temperature
23.10.2008	1.829040	5.2	2.254805	5.1	0.000000	5.9
24.10.2008	2.743552	6.6	0.751587	7.6	1.674263	4.9
25.10.2008	2.743544	5.0	0.751573	3.1	0.000000	4.6
26.10.2008	0.000000	2.4	6.764028	2.5	0.000000	5.7
27.10.2008	0.914509	4.9	3.757722	4.4	0.000000	4.6
28.10.2008	3.658026	4.5	1.503060	5.1	3.348519	5.1
29.10.2008	4.572518	6.6	3.757579	5.5	0.000000	3.1
30.10.2008	1.829002	4.3	2.254504	5.1	1.674258	4.8
31.10.2008	3.657993	5.7	3.757436	6.4	0.000000	3.2
01.11.2008	4.572477	9.0	3.005892	8.3	5.022770	4.3
02.11.2008	2.743478	7.7	4.508752	7.7	0.000000	6.3
03.11.2008	3.657960	6.2	4.508666	5.1	0.000000	4.5
04.11.2008	1.828974	1.4	6.011440	0.9	0.000000	5.4
05.11.2008	2.743453	0.5	3.005663	-0.4	0.000000	5.7
06.11.2008	2.743445	-0.2	3.005606	-0.4	0.000000	1.4
07.11.2008	1.828958	-3.2	9.016646	-3.6	0.000000	7.4
08.11.2008	2.743429	-5.4	7.513728	-5.1	0.000000	1.7
09.11.2008	1.828947	-4.3	4.508151	-4.9	0.000000	-5.8
10.11.2008	4.572354	-1.1	13.524197	-1.3	0.000000	-4.4
11.11.2008	2.743404	2.0	4.507980	1.1	3.348499	-3.9
12.11.2008	2.743396	4.4	6.010526	3.3	0.000000	-2.7
13.11.2008	2.743388	2.3	13.523425	1.6	0.000000	-1.1
14.11.2008	4.572299	1.3	6.010297	-0.2	0.000000	-0.8
15.11.2008	1.828914	0.3	21.035639	-0.1	0.000000	-1.4
16.11.2008	2.743363	3.6	3.005034	2.7	0.000000	-2.3
17.11.2008	8.230064	4.1	13.522397	3.5	0.000000	-4.7
18.11.2008	3.657795	4.7	10.517220	3.8	0.000000	-5.9
19.11.2008	5.486677	3.0	9.014588	4.9	0.000000	-7.8
20.11.2008	6.401104	-0.9	9.014417	-0.7	0.000000	-7.8
21.11.2008	4.572203	1.1	12.018994	0.2	1.674242	-5.9
22.11.2008	4.572190	4.8	10.516420	3.6	0.000000	-6.4
23.11.2008	4.572176	5.1	10.516220	1.7	0.000000	-1.1
24.11.2008	6.401027	2.1	6.009154	3.8	0.000000	0.1
25.11.2008	3.657719	0.1	12.018080	-1.6	0.000000	-2.9
26.11.2008	9.144270	-1.7	9.013389	-3.3	0.000000	-4.1
27.11.2008	2.743273	2.5	12.017623	0.9	0.000000	-3.7
28.11.2008	6.400950	-0.7	4.506523	-2.0	0.000000	1.7
29.11.2008	1.828837	1.1	1.502146	0.8	0.000000	-3.4
30.11.2008	5.486496	1.5	7.510586	0.8	0.000000	-3.7
01.12.2008	7.315306	-2.9	6.008355	-4.8	0.000000	-20.1
02.12.2008	1.828821	-0.9	3.004120	-0.6	0.000000	-27.6

Data	Chelyabinsk		Yekaterinburg		Barnaul	
	RI rates	Temperature	RI rates	Temperature	RI rates	Temperature
03.12.2008	3.657631	0.7	3.004063	1.1	0.000000	-24.1
04.12.2008	5.486430	0.2	4.506009	-0.1	0.000000	-16.1
05.12.2008	7.315218	1.4	3.003949	-0.5	0.000000	-8.4
06.12.2008	0.914400	6.5	0.000000	3.8	0.000000	-3.1
07.12.2008	4.571984	2.1	0.000000	1.8	0.000000	-1.7
08.12.2008	6.400759	1.2	0.000000	2.2	0.000000	-2.9
09.12.2008	8.229522	3.2	0.000000	1.3	0.000000	-0.6
10.12.2008	5.486332	-0.8	1.501832	-2.1	0.000000	-2.8
11.12.2008	3.657543	-2.3	3.003606	-2.9	0.000000	-14.1
12.12.2008	3.657532	-1.4	0.000000	-1.4	0.000000	-15.1
13.12.2008	1.828761	-4.2	0.000000	-4.5	0.000000	-14.2
14.12.2008	5.486266	-11.9	0.000000	-13.8	0.000000	-7.7
15.12.2008	9.143749	-15.5	1.501689	-14.5	0.000000	-20.6
16.12.2008	7.314977	-10.7	3.003321	-11.9	0.000000	-15.4
17.12.2008	5.486217	-9.2	1.501632	-12.8	0.000000	-10.4
18.12.2008	3.657467	-14.1	7.508017	-15.4	0.000000	-16.6
19.12.2008	7.314912	-15.7	13.514173	-16.2	0.000000	-17.9
20.12.2008	3.657445	-17.1	6.006185	-15.3	0.000000	-19.0
21.12.2008	3.657434	-16.1	3.003036	-16.6	0.000000	-21.7
22.12.2008	5.486134	-14.7	4.504468	-15.5	0.000000	-15.4
23.12.2008	4.571765	-13.2	3.002921	-15.7	0.000000	-13.9
24.12.2008	13.715254	-13.7	3.002864	-16.3	0.000000	-15.9
25.12.2008	6.400433	-14.4	6.005615	-16.3	0.000000	-18.3
26.12.2008	6.400413	-11.6	4.504125	-13.2	0.000000	-18.9
27.12.2008	4.571710	-8.5	7.506733	-10.3	0.000000	-21.1
28.12.2008	5.486036	-7.1	7.506590	-9.5	0.000000	-19.1
29.12.2008	8.229029	-11.1	4.503869	-10.5	0.000000	-11.1
30.12.2008	4.571669	-9.2	10.508827	-8.8	0.000000	-11.6
31.12.2008	6.400318	-5.9	3.002465	-8.1	0.000000	-11.7
01.01.2009	4.571642	-8.9	1.501204	-7.3	0.000000	-12.3
02.01.2009	5.485469	-13.1	1.501150	-11.9	0.000000	-15.3
03.01.2009	4.570807	-12.8	3.002192	-13.2	0.000000	-15.7
04.01.2009	2.742234	-18.9	5.253646	-19.3	0.000000	-11.6
05.01.2009	7.311957	-17.7	6.754445	-21.2	0.000000	-11.3
06.01.2009	4.569557	-14.1	4.502801	-19.9	0.000000	-15.8
07.01.2009	8.224451	-14.1	3.001759	-16.0	0.000000	-12.3
08.01.2009	5.482468	-11.1	5.252890	-13.3	0.000000	-12.3
09.01.2009	4.568306	-14.6	4.502315	-17.3	0.000000	-17.6
10.01.2009	8.222202	-15.6	6.753230	-15.6	0.000000	-13.9
11.01.2009	10.961936	-17.2	3.751659	-20.4	0.000000	-8.1
12.01.2009	9.134114	-15.0	5.252134	-15.0	0.000000	-6.6

Data	Chelyabinsk		Yekaterinburg		Barnaul	
	RI rates	Temperature	RI rates	Temperature	RI rates	Temperature
13.01.2009	4.566640	-16.7	9.003334	-15.4	1.672821	-8.9
14.01.2009	13.698672	-10.9	6.752257	-10.8	0.000000	-9.3
15.01.2009	7.305293	-1.1	6.001791	-2.1	0.000000	-12.3
16.01.2009	6.391549	-5.4	10.502756	-9.4	0.000000	-11.9
17.01.2009	14.607922	-10.4	5.251189	-12.5	1.672358	-13.9
18.01.2009	10.042032	-9.9	8.251571	-11.7	0.000000	-12.8
19.01.2009	9.128288	-10.6	12.001854	-13.4	6.688506	-14.9
20.01.2009	10.040202	-13.6	6.000711	-15.1	0.000000	-24.9
21.01.2009	10.951950	-14.4	7.500619	-15.4	3.343790	-27.7
22.01.2009	5.475476	-16.1	9.750454	-16.4	3.343559	-22.1
23.01.2009	5.474977	-10.5	9.750103	-8.9	5.014991	-19.8
24.01.2009	6.386892	-2.2	8.249790	-2.7	1.671548	-18.7
25.01.2009	3.649320	-1.4	6.749586	-3.9	3.342865	-7.6
26.01.2009	9.122469	-5.1	9.749051	-9.9	3.342634	-8.8
27.01.2009	2.736492	-9.1	6.749100	-12.7	3.342403	-20.2
28.01.2009	10.944970	-11.7	13.497714	-10.9	6.684343	-22.7
29.01.2009	1.823996	-18.8	8.248307	-20.4	1.670970	-19.8
30.01.2009	11.854892	-21.9	9.747648	-23.4	3.341709	-20.3
31.01.2009	7.294654	-16.5	13.496258	-20.7	1.670739	-25.6
01.02.2009	6.382242	-16.2	5.998121	-24.5	3.341247	-23.9
02.02.2009	9.116658	-17.4	9.746596	-19.7	3.341016	-19.3
03.02.2009	6.381080	-10.1	7.497112	-10.8	3.340785	-13.3
04.02.2009	5.469000	-7.9	5.997474	-9.3	5.010831	-7.7
05.02.2009	10.025587	-6.7	10.495202	-9.2	1.670162	-9.2
06.02.2009	4.556671	-13.3	11.244455	-19.3	1.670046	-5.3
07.02.2009	7.290010	-23.8	14.242464	-26.9	5.009793	-5.9
08.02.2009	3.644673	-25.9	8.994917	-25.1	3.339631	-6.8
09.02.2009	4.555427	-16.2	11.992792	-14.5	3.339400	-22.4
10.02.2009	4.555013	-12.8	8.244748	-15.1	5.008754	-24.9
11.02.2009	2.732759	-9.7	10.492938	-11.9	5.008408	-29.3
12.02.2009	5.465022	-2.3	8.993624	-5.1	3.338708	-31.4
13.02.2009	7.286034	-1.8	11.241626	-3.9	8.346194	-28.9
14.02.2009	5.464028	-7.4	8.992977	-5.4	8.345617	-20.7
15.02.2009	3.642355	-10.2	10.491430	-11.7	5.007024	-24.2
16.02.2009	8.194553	-13.7	14.987218	-10.8	3.337786	-28.3
17.02.2009	2.731269	-9.8	7.493340	-8.9	0.000000	-27.2
18.02.2009	12.744766	-7.9	14.986141	-8.3	3.337325	-17.3
19.02.2009	8.192319	-10.4	11.988482	-11.0	0.000000	-13.9
20.02.2009	5.461050	-18.9	7.492532	-20.8	3.336864	-20.9
21.02.2009	6.370646	-13.7	5.244584	-17.2	1.668317	-24.3
22.02.2009	4.550048	-10.3	8.241192	-14.3	1.668202	-21.0

Data	Chelyabinsk		Yekaterinburg		Barnaul	
	RI rates	Temperature	RI rates	Temperature	RI rates	Temperature
23.02.2009	9.099270	-8.6	8.990069	-10.0	1.668086	-19.4
24.02.2009	9.098444	-8.5	7.491455	-10.8	5.003914	-19.4
25.02.2009	11.826902	-8.8	13.484134	-10.1	0.000000	-17.3
26.02.2009	8.187112	-12.1	7.490916	-7.9	0.000000	-13.7
27.02.2009	13.643948	-8.4	11.235971	-7.7	8.338130	-11.2
28.02.2009	7.276112	-7.5	8.239416	-5.4	0.000000	-14.3
01.03.2009	7.275451	-6.5	9.737142	-4.4	5.002187	-13.5
02.03.2009	10.002837	-7.4	10.485776	-5.6	0.000000	-10.5
03.03.2009	5.455598	-6.6	7.489571	-9.1	0.000000	-11.1
04.03.2009	5.455103	-3.9	14.978603	-5.2	6.668203	-9.7
05.03.2009	4.545506	-5.8	5.991226	-5.7	5.000807	-5.8
06.03.2009	8.181169	-4.1	7.488764	-2.3	5.000462	-8.1
07.03.2009	2.726809	-5.1	6.739645	-6.4	1.666706	-8.0
08.03.2009	5.453123	-6.3	8.237048	-7.4	1.666591	-7.8
09.03.2009	6.361400	-9.1	5.990365	-4.2	6.665904	-8.8
10.03.2009	9.995579	-7.4	11.231532	-3.3	1.666361	-13.1
11.03.2009	11.811886	-1.1	7.487419	-0.1	1.666246	-12.2
12.03.2009	13.627863	1.2	8.235865	0.6	6.664525	-4.4
13.03.2009	14.535069	-0.6	8.984257	-1.7	1.666016	0.2
14.03.2009	6.358516	-7.3	9.732596	-7.8	4.997704	-1.2
15.03.2009	9.082771	-7.8	10.480881	-9.6	6.663146	-5.0
16.03.2009	6.357363	-7.8	7.486075	-8.6	8.328359	-10.6
17.03.2009	10.897349	-5.5	14.223031	-5.2	8.327785	-13.7
18.03.2009	9.080301	-3.6	17.216735	-5.2	3.330884	-11.0
19.03.2009	12.711270	-3.3	12.724956	-6.2	8.326637	-10.2
20.03.2009	8.170790	-6.0	11.975999	-5.8	11.656488	-10.7
21.03.2009	7.262266	-6.3	8.233204	-4.3	1.665098	-10.4
22.03.2009	11.800114	-7.0	12.723585	-2.8	8.324916	-7.0
23.03.2009	8.168569	-1.2	10.477871	-2.3	6.659474	-0.2
24.03.2009	16.335659	-3.5	13.471064	-5.3	8.323769	-0.8
25.03.2009	16.334179	0.9	6.735290	-0.7	1.664639	-0.4
26.03.2009	6.351605	0.4	6.735049	-2.6	8.322622	2.3
27.03.2009	10.887480	3.0	5.986495	2.1	9.986458	6.8
28.03.2009	8.164871	3.6	10.475990	2.7	6.657180	7.1
29.03.2009	4.535629	8.4	7.482582	6.4	4.992541	4.4
30.03.2009	12.698610	10.3	11.971701	7.4	6.656264	2.6
31.03.2009	7.255692	10.9	15.712294	8.4	3.327903	2.8
01.04.2009	9.068794	10.9	8.978131	9.1	6.655347	1.8
02.04.2009	5.440784	8.6	9.725960	8.9	6.654889	5.4
03.04.2009	6.347007	9.6	9.725611	8.4	8.318038	8.1
04.04.2009	8.159698	0.6	6.732874	-2.5	1.663493	9.3

Data	Chelyabinsk		Yekaterinburg		Barnaul	
	RI rates	Temperature	RI rates	Temperature	RI rates	Temperature
05.04.2009	5.439307	-2.8	7.480702	-6.1	3.326757	4.0
06.04.2009	9.064691	-3.2	11.220651	-5.2	4.989792	5.3
07.04.2009	3.625548	-1.3	7.480165	-3.2	0.000000	0.4
08.04.2009	2.718915	-3.9	14.211804	-6.0	1.663035	2.8
09.04.2009	4.531116	-5.3	9.723517	-7.0	8.314604	6.3
10.04.2009	9.061411	-2.7	11.966976	-5.6	11.639644	0.3
11.04.2009	3.624237	-1.4	12.714456	-2.3	6.650767	2.3
12.04.2009	1.811955	-1.1	8.226706	-2.0	6.650310	5.4
13.04.2009	5.435372	-0.8	13.461400	-1.1	9.974779	6.7
14.04.2009	5.434881	1.1	11.965259	0.8	9.974092	5.2
15.04.2009	3.622926	4.6	5.982415	4.9	3.324469	2.0
16.04.2009	7.245197	8.6	11.964401	7.9	4.986360	2.8
17.04.2009	9.961246	2.8	11.963972	-1.2	8.310029	2.9
18.04.2009	4.527430	-0.2	8.224936	0.2	6.647566	7.8
19.04.2009	9.054041	9.4	11.963114	6.6	4.985331	2.6
20.04.2009	13.579835	9.8	14.953356	5.4	9.969977	6.6
21.04.2009	7.241924	1.7	8.971692	1.6	3.323097	10.5
22.04.2009	8.146428	3.1	14.204669	0.8	6.645737	7.3
23.04.2009	8.145692	0.9	7.475874	-0.4	6.645281	9.7
24.04.2009	5.429971	2.3	10.465848	0.8	4.983618	10.1
25.04.2009	1.809827	2.2	8.970405	1.5	3.322183	5.2
26.04.2009	4.524159	1.5	8.222576	0.9	3.321955	4.6
27.04.2009	8.142750	3.9	8.222282	1.3	4.982590	8.4
28.04.2009	9.951351	10.5	8.221987	7.5	3.321498	7.9
29.04.2009	9.950453	14.6	8.969118	12.1	3.321270	6.7
30.04.2009	6.331535	14.8	5.231798	8.3	1.660521	10.4
01.05.2009	2.713270	3.7	7.473730	-0.1	3.320814	11.6
02.05.2009	3.617367	1.5	6.726115	1.9	3.320586	15.6
03.05.2009	7.234080	3.7	10.462471	1.4	9.961072	9.7
04.05.2009	11.754319	5.7	3.736463	3.1	4.980194	5.1
05.05.2009	5.424581	7.9	12.703519	2.5	3.319901	5.1
06.05.2009	8.136137	10.0	7.472390	8.9	8.299183	5.4
07.05.2009	1.807867	15.9	5.977698	15.8	4.979168	8.3
08.05.2009	2.711556	17.3	5.230298	14.9	0.000000	12.6
09.05.2009	4.518853	17.9	7.471587	17.3	0.000000	16.3
10.05.2009	2.711067	15.8	5.977055	14.4	1.659381	19.3
11.05.2009	3.614430	14.7	5.976841	13.7	3.318533	20.1
12.05.2009	7.228208	17.8	6.723705	15.3	6.636611	20.9
13.05.2009	3.613778	18.6	14.941032	18.3	6.636156	14.7
14.05.2009	1.806726	19.3	11.205372	18.4	4.976775	12.1
15.05.2009	2.709845	18.2	9.710975	16.7	1.658811	16.6

Data	Chelyabinsk		Yekaterinburg		Barnaul	
	RI rates	Temperature	RI rates	Temperature	RI rates	Temperature
16.05.2009	3.612801	13.4	9.710627	12.4	3.317395	19.8
17.05.2009	4.515594	6.9	5.228612	4.6	6.634334	18.7
18.05.2009	6.321262	5.2	8.216095	5.5	8.292348	13.5
19.05.2009	6.320692	11.9	8.215801	12.5	6.633423	12.1
20.05.2009	4.514373	7.3	6.721778	5.4	3.316484	16.4
21.05.2009	2.708380	4.1	4.481025	2.6	3.316256	14.6
22.05.2009	0.902712	5.7	12.695782	5.1	6.632058	4.8
23.05.2009	0.902631	8.2	5.974272	7.5	1.657901	4.7
24.05.2009	3.610197	13.7	2.987029	12.2	1.657787	5.0
25.05.2009	0.902468	16.1	1.493461	16.6	3.315346	5.5
26.05.2009	6.316707	16.6	3.733519	15.3	3.315119	6.4
27.05.2009	7.218443	15.6	2.986708	12.4	4.972337	10.9
28.05.2009	3.608897	16.4	5.226552	14.9	6.629328	16.9
29.05.2009	4.510715	20.3	2.239871	20.1	1.657218	22.8
30.05.2009	3.608247	19.3	0.746597	18.3	0.000000	21.2
31.05.2009	2.705941	12.9	1.493140	12.2	0.000000	15.5
01.06.2009	3.607597	9.8	2.986173	9.5	4.970633	19.3
02.06.2009	4.509090	16.0	2.986066	14.0	1.656764	17.9
03.06.2009	9.017369	17.8	2.239470	15.2	0.000000	14.7
04.06.2009	7.213246	22.9	1.492926	21.2	1.656537	12.8
05.06.2009	3.606298	24.9	1.492873	23.2	3.312846	15.9
06.06.2009	0.000000	24.9	0.000000	24.1	0.000000	10.9
07.06.2009	2.704237	26.1	3.731915	24.6	1.656196	16.4
08.06.2009	7.210650	18.6	3.731781	17.9	0.000000	21.0
09.06.2009	4.506251	21.6	0.746329	19.5	1.655969	18.7
10.06.2009	4.505846	19.9	3.731514	17.6	1.655856	20.8
11.06.2009	3.604352	15.1	2.238828	15.4	3.311484	18.3
12.06.2009	1.802014	18.7	2.984997	17.5	6.622515	8.7
13.06.2009	2.702778	21.4	1.492445	21.5	1.655515	7.3
14.06.2009	2.702535	18.1	1.492392	16.9	1.655402	10.1
15.06.2009	3.603056	18.2	5.223185	18.5	1.655289	13.2
16.06.2009	1.801366	18.6	3.730713	19.5	4.965526	9.9
17.06.2009	4.503010	24.9	2.238347	24.3	6.620247	8.9
18.06.2009	2.701563	24.4	0.746089	24.1	0.000000	11.5
19.06.2009	1.800880	23.8	3.730312	23.4	1.654835	14.9
20.06.2009	4.501796	17.1	0.000000	16.3	0.000000	14.3
21.06.2009	3.601113	17.8	1.492018	17.1	1.654608	15.7
22.06.2009	4.500987	18.8	2.983929	18.2	3.308990	15.4
23.06.2009	7.200932	14.6	0.745956	13.4	1.654382	16.8
24.06.2009	5.400214	11.4	2.237787	10.3	4.962806	15.3
25.06.2009	5.399729	14.9	0.000000	13.9	3.308311	13.4

Data	Chelyabinsk		Yekaterinburg		Barnaul	
	RI rates	Temperature	RI rates	Temperature	RI rates	Temperature
26.06.2009	5.399244	13.3	2.237627	11.2	8.270211	12.5
27.06.2009	3.599173	15.4	0.000000	15.6	1.653929	12.6
28.06.2009	1.799425	17.4	3.729111	16.8	8.269079	10.4
29.06.2009	3.598526	17.3	0.745796	14.6	1.653703	12.9
30.06.2009	0.899551	10.6	0.000000	9.8	3.307179	18.3
01.07.2009	2.698410	11.4	0.000000	12.1	3.306953	13.9
02.07.2009	5.396336	16.4	0.745715	14.2	9.920179	12.9
03.07.2009	0.899309	18.6	2.237066	13.7	1.653250	16.6
04.07.2009	4.496139	16.6	2.236986	15.5	6.612548	20.1
05.07.2009	3.596588	19.0	1.491271	15.6	1.653024	19.9
06.07.2009	2.697199	17.6	1.491218	14.3	0.000000	22.0
07.07.2009	3.595943	17.8	1.491164	15.3	3.305595	22.0
08.07.2009	1.797810	15.6	2.236666	14.9	3.305369	17.8
09.07.2009	1.797649	13.7	0.745529	14.4	0.000000	20.6
10.07.2009	1.797488	14.8	1.491004	17.7	0.000000	22.7
11.07.2009	1.797326	19.3	0.745475	20.3	0.000000	19.3
12.07.2009	0.898583	19.1	0.000000	20.4	1.652233	19.6
13.07.2009	5.391012	17.6	1.490844	18.4	3.304239	19.7
14.07.2009	1.796843	18.2	2.236187	19.2	6.608027	20.7
15.07.2009	5.390045	19.7	0.745369	20.8	1.651894	21.7
16.07.2009	0.898260	20.9	1.490684	20.0	4.955343	21.3
17.07.2009	3.592719	18.2	0.000000	18.4	1.651668	19.9
18.07.2009	0.000000	20.8	0.745289	19.8	0.000000	20.6
19.07.2009	4.490094	21.3	0.745262	18.2	4.954327	19.8
20.07.2009	1.795877	14.9	0.745236	13.4	1.651329	19.4
21.07.2009	0.000000	14.3	0.745209	12.9	3.302433	18.9
22.07.2009	2.693332	17.6	1.490365	16.8	1.651104	11.2
23.07.2009	1.795394	20.6	1.490311	19.6	4.952973	13.2
24.07.2009	2.692849	18.1	1.490258	16.8	6.603512	15.3
25.07.2009	2.692608	16.1	2.235307	15.2	3.301531	15.6
26.07.2009	0.000000	16.6	0.000000	16.6	3.301305	18.3
27.07.2009	4.486876	19.4	0.000000	19.6	1.650540	19.7
28.07.2009	1.794590	21.8	0.745023	20.8	3.300854	19.4
29.07.2009	2.691644	15.1	1.489992	16.0	4.950943	19.3
30.07.2009	0.897134	16.7	0.000000	15.9	0.000000	18.1
31.07.2009	1.794108	15.9	0.000000	15.2	0.000000	17.1
01.08.2009	1.793947	13.8	0.000000	13.4	3.299953	18.4
02.08.2009	1.793786	15.2	0.744889	16.6	3.299727	16.6
03.08.2009	1.793626	15.4	0.000000	16.2	3.299502	18.6
04.08.2009	2.690198	18.4	1.489673	17.8	0.000000	17.5
05.08.2009	2.689957	19.8	1.489619	16.2	1.649526	15.8

Data	Chelyabinsk		Yekaterinburg		Barnaul	
	RI rates	Temperature	RI rates	Temperature	RI rates	Temperature
06.08.2009	2.689717	17.2	0.744783	14.1	0.000000	15.1
07.08.2009	0.000000	11.9	0.744756	10.7	0.000000	16.9
08.08.2009	1.792823	12.2	0.744730	10.9	3.298376	20.8
09.08.2009	0.896332	10.5	0.000000	10.4	0.000000	19.3
10.08.2009	0.000000	11.4	0.744677	10.2	0.000000	19.7
11.08.2009	1.792342	12.0	1.489300	11.4	0.000000	16.2
12.08.2009	1.792182	11.4	0.744623	11.1	3.297476	13.2
13.08.2009	2.688033	16.0	1.489194	15.6	1.648625	12.1
14.08.2009	1.791861	17.9	0.744570	17.9	0.000000	13.0
15.08.2009	1.791701	17.5	0.000000	16.1	0.000000	13.9
16.08.2009	1.791541	18.3	2.978068	17.1	3.296576	15.0
17.08.2009	0.895690	15.5	2.977962	15.2	1.648176	16.7
18.08.2009	1.791221	16.0	0.744464	16.2	0.000000	17.7
19.08.2009	0.895530	18.5	0.000000	18.1	0.000000	16.7
20.08.2009	2.686351	19.7	0.744411	19.0	1.647838	17.0
21.08.2009	4.476851	21.2	1.488768	19.6	6.590904	14.9
22.08.2009	0.000000	20.6	0.744358	19.2	3.295228	15.7
23.08.2009	3.580841	19.6	0.000000	19.3	0.000000	16.0
24.08.2009	1.790260	17.2	1.488609	16.7	0.000000	17.1
25.08.2009	2.685151	16.1	0.000000	13.6	0.000000	17.4
26.08.2009	4.474852	17.7	1.488503	18.4	0.000000	18.1
27.08.2009	0.894890	17.3	0.000000	17.4	0.000000	18.6
28.08.2009	0.894810	17.3	0.000000	16.7	0.000000	17.4
29.08.2009	0.894731	16.7	0.744172	15.4	3.293656	16.6
30.08.2009	4.473253	12.9	1.488290	13.2	1.646716	16.9
31.08.2009	0.000000	8.8	2.232355	7.4	3.293207	18.3
01.09.2009	3.577963	12.3	0.000000	10.7	0.000000	7.3
02.09.2009	0.894411	15.4	0.744065	14.3	1.646379	7.9
03.09.2009	5.365987	15.7	1.488078	15.5	4.938800	10.1
04.09.2009	0.894251	16.2	0.000000	14.4	0.000000	9.7
05.09.2009	1.788343	16.9	0.743986	15.9	1.646042	9.3
06.09.2009	3.576367	17.0	0.743959	15.9	4.937791	9.1
07.09.2009	1.788024	16.8	1.487865	15.4	0.000000	9.9
08.09.2009	3.575729	17.4	0.743906	15.8	0.000000	11.3
09.09.2009	6.256967	17.4	4.463277	17.1	1.645594	12.7
10.09.2009	1.787545	15.5	1.487706	14.3	3.290964	13.6
11.09.2009	0.893693	16.2	0.743826	14.9	0.000000	13.8
12.09.2009	1.787226	16.0	0.000000	14.3	1.645258	15.4
13.09.2009	0.893534	15.7	0.743773	13.1	3.290292	15.1
14.09.2009	0.893454	11.6	2.974987	10.3	0.000000	11.8
15.09.2009	0.893374	11.6	0.000000	10.3	3.289844	10.0

Data	Chelyabinsk		Yekaterinburg		Barnaul	
	RI rates	Temperature	RI rates	Temperature	RI rates	Temperature
16.09.2009	0.893295	10.3	0.743694	8.8	0.000000	12.4
17.09.2009	0.893215	10.4	0.743667	8.9	0.000000	6.2
18.09.2009	1.786270	14.6	1.487281	12.9	1.644586	6.4
19.09.2009	0.000000	14.8	2.230842	12.1	4.933423	8.3
20.09.2009	1.785952	7.4	0.743588	6.7	3.288725	13.8
21.09.2009	0.892896	4.7	0.743561	5.6	0.000000	15.9
22.09.2009	1.785634	8.2	1.487069	7.0	0.000000	11.2
23.09.2009	3.570949	11.8	2.974032	10.5	3.288053	7.5
24.09.2009	0.000000	11.4	1.486963	10.2	1.643915	11.3
25.09.2009	0.892578	11.7	2.973820	9.4	3.287606	13.5
26.09.2009	0.000000	9.4	0.743429	7.3	0.000000	16.0
27.09.2009	0.892419	10.4	2.230206	7.7	0.000000	14.2
28.09.2009	2.677019	12.3	2.973502	10.8	3.286935	11.3
29.09.2009	0.892260	13.4	1.486698	11.7	1.643356	10.9
30.09.2009	4.460904	12.9	0.000000	14.2	0.000000	12.8
01.10.2009	1.784203	10.8	0.743296	11.5	0.000000	13.7
02.10.2009	0.892022	5.9	0.000000	5.4	1.643021	16.1
03.10.2009	1.783885	8.3	2.229729	6.3	1.642909	13.4
04.10.2009	2.675589	4.0	0.743217	2.1	0.000000	7.6
05.10.2009	2.675351	5.0	1.486380	3.9	0.000000	8.9
06.10.2009	4.458522	6.1	4.458981	4.6	4.927722	5.3
07.10.2009	0.891625	4.4	5.201959	3.2	1.642462	7.4
08.10.2009	0.000000	5.4	2.972442	5.7	1.642351	10.8
09.10.2009	3.565865	8.4	0.000000	8.9	0.000000	11.3
10.10.2009	0.891387	8.5	2.972230	8.4	0.000000	11.3
11.10.2009	0.000000	2.8	3.715155	0.6	0.000000	10.3
12.10.2009	1.782457	1.8	1.486009	-1.0	1.641904	4.2
13.10.2009	5.346895	6.5	2.228934	3.1	0.000000	1.2
14.10.2009	4.455349	15.4	0.000000	12.6	0.000000	2.2
15.10.2009	0.000000	15.3	2.228776	11.3	4.924709	7.9
16.10.2009	0.890911	9.4	2.228696	6.8	4.924374	1.9
17.10.2009	0.890832	11.4	1.485744	9.9	1.641347	1.7
18.10.2009	1.781506	12.8	0.000000	11.5	3.282470	5.2
19.10.2009	2.672021	14.2	2.228458	12.6	1.641124	2.5
20.10.2009	1.781189	15.0	0.742793	11.3	3.282024	6.1
21.10.2009	8.014639	6.3	1.485533	3.9	0.000000	8.3
22.10.2009	0.890436	1.1	2.228220	0.1	1.640789	0.3
23.10.2009	4.451786	-0.8	0.000000	-0.5	0.000000	-4.9
24.10.2009	1.780556	1.0	0.742687	-0.6	3.281133	-3.4
25.10.2009	1.780398	-5.1	0.000000	-6.3	0.000000	-1.0
26.10.2009	0.000000	-6.4	1.485268	-5.8	0.000000	-5.9

Data	Chelyabinsk		Yekaterinburg		Barnaul	
	RI rates	Temperature	RI rates	Temperature	RI rates	Temperature
27.10.2009	4.450204	-2.4	0.000000	-3.6	3.280465	-10.4
28.10.2009	0.000000	-0.3	0.000000	-1.2	6.560485	-9.3
29.10.2009	3.559530	2.3	0.000000	0.8	1.640010	-8.2
30.10.2009	0.889804	2.3	0.000000	1.2	6.559594	-5.1
31.10.2009	2.669174	1.2	0.000000	0.1	0.000000	-5.1
01.11.2009	1.779291	-2.1	0.000000	-2.3	3.279352	-0.6
02.11.2009	2.668700	-4.2	1.484898	-7.0	3.279129	1.9
03.11.2009	3.557950	-6.7	2.969690	-7.0	11.476174	1.7
04.11.2009	5.336451	-3.8	0.000000	-3.2	1.639342	0.6
05.11.2009	6.225307	-2.9	1.484739	-4.2	6.556924	-7.8
06.11.2009	2.667752	-3.5	2.227030	-4.3	6.556479	-16.8
07.11.2009	3.556687	-9.4	0.742317	-6.8	4.917026	-17.6
08.11.2009	6.223650	-12.0	0.000000	-11.2	6.555590	-18.7
09.11.2009	2.667042	-6.4	2.969056	-6.0	1.638786	-18.5
10.11.2009	3.555740	-2.6	2.226712	-3.7	1.638675	-18.5
11.11.2009	4.444281	-9.3	2.226633	-11.9	1.638564	-16.3
12.11.2009	5.332664	-9.7	1.484369	-12.4	4.915359	-15.1
13.11.2009	4.443493	-4.7	0.000000	-5.8	4.915026	-18.3
14.11.2009	3.554479	-5.3	1.484264	-2.3	1.638231	-17.9
15.11.2009	0.888541	-3.9	0.742105	-1.4	4.914359	-13.2
16.11.2009	4.442310	-5.9	4.452474	-6.2	4.914026	-7.2
17.11.2009	3.553533	-7.3	0.742053	-11.9	4.913693	-7.7
18.11.2009	3.553218	-2.1	0.000000	-5.2	1.637787	-9.2
19.11.2009	4.441129	-1.9	0.742000	-2.4	1.637676	-5.9
20.11.2009	5.328882	-5.3	2.967893	-5.6	1.637565	-5.6
21.11.2009	3.552273	-6.7	2.967788	-3.3	3.274908	-6.5
22.11.2009	3.551958	-2.8	2.967682	-3.9	4.912028	-0.9
23.11.2009	2.663733	-5.1	0.741894	-8.8	3.274464	0.0
24.11.2009	4.439161	-0.9	2.225603	-6.9	4.911363	-7.9
25.11.2009	2.663261	1.9	1.483683	0.9	6.548040	-5.1
26.11.2009	0.000000	1.8	3.709074	0.7	6.547597	-0.4
27.11.2009	7.100770	0.6	1.483577	-8.4	14.731095	0.0
28.11.2009	5.325105	1.6	3.708811	0.4	4.910032	-4.1
29.11.2009	2.662317	1.3	6.675621	2.1	11.455966	-3.2
30.11.2009	3.549441	3.0	5.191965	1.6	1.636456	-0.3
01.12.2009	5.323691	0.2	4.450098	-0.1	0.000000	-1.6
02.12.2009	10.646438	-0.3	3.708283	-0.1	0.000000	-19.4
03.12.2009	4.435623	0.7	2.966521	1.2	6.544493	-14.7
04.12.2009	3.548184	-0.9	0.741604	-2.3	1.636013	-4.7
05.12.2009	4.434838	-8.7	2.966310	-10.1	11.451313	-4.1
06.12.2009	3.547556	-19.1	4.449306	-20.1	6.543164	-11.3

Data	Chelyabinsk		Yekaterinburg		Barnaul	
	RI rates	Temperature	RI rates	Temperature	RI rates	Temperature
07.12.2009	5.320863	-18.2	2.966099	-19.4	8.178402	-21.2
08.12.2009	7.093856	-12.9	2.224495	-14.2	6.542279	-30.1
09.12.2009	5.319921	-11.9	2.224416	-12.8	1.635459	-27.1
10.12.2009	12.412051	-19.6	2.224337	-18.8	1.635348	-18.8
11.12.2009	4.432483	-14.9	3.707096	-15.7	13.081901	-19.9
12.12.2009	3.545673	-16.8	0.741393	-16.7	14.716142	-16.4
13.12.2009	2.659019	-10.5	5.930932	-14.1	6.540065	-19.3
14.12.2009	7.090091	-12.9	5.189381	-13.3	1.634906	-16.8
15.12.2009	4.430915	-23.7	3.706569	-29.8	6.539180	-13.3
16.12.2009	9.747151	-25.8	4.447724	-28.3	9.808107	-10.1
17.12.2009	4.430131	-27.1	2.223783	-27.7	8.172869	-12.2
18.12.2009	5.315687	-26.8	4.447408	-21.6	4.903390	-14.9
19.12.2009	3.543478	-25.9	2.964833	-23.7	1.634353	-11.8
20.12.2009	4.428956	-21.3	5.188274	-23.5	13.073938	-7.4
21.12.2009	2.657139	-22.6	3.705778	-25.1	11.438922	-19.4
22.12.2009	3.542538	-22.9	2.964517	-22.8	4.902064	-33.4
23.12.2009	7.084451	-18.3	5.928823	-18.7	6.535643	-35.9
24.12.2009	9.740259	-9.2	4.446459	-8.8	1.633800	-26.6
25.12.2009	4.426999	-4.8	6.669452	-5.7	1.633690	-22.1
26.12.2009	0.885322	-6.6	8.151263	-11.7	8.167897	-11.3
27.12.2009	1.770487	-8.4	3.704988	-13.9	3.266938	-14.7
28.12.2009	0.885165	-1.9	5.186798	-5.7	0.000000	-26.4
29.12.2009	4.425435	-7.2	2.222835	-16.3	0.000000	-14.0
30.12.2009	7.080070	-20.3	3.704593	-23.3	0.000000	-8.1
31.12.2009	4.424653	-29.6	0.000000	-28.9	0.000000	-29.7
01.01.2010	7.078819	-28.9	0.000000	-29.6	0.000000	-26.7
02.01.2010	8.848503	-23.7	4.445192	-23.7	0.000000	-29.9
03.01.2010	4.424241	-19.7	2.222595	-20.2	0.000000	-22.9
04.01.2010	4.424231	-19.4	5.926915	-19.2	0.000000	-20.6
05.01.2010	5.309064	-16.0	8.149503	-16.6	0.000000	-21.2
06.01.2010	6.193893	-22.6	2.963454	-23.7	0.000000	-30.3
07.01.2010	7.963558	-22.1	4.445177	-25.6	0.000000	-35.6
08.01.2010	8.848377	-29.3	3.704312	-32.3	0.000000	-35.8
09.01.2010	4.424178	-21.7	0.740862	-22.4	0.000000	-34.2
10.01.2010	2.654501	-13.9	7.408614	-15.7	0.000000	-25.9
11.01.2010	9.733146	-19.4	6.667748	-22.0	3.265878	-11.1
12.01.2010	5.308976	-16.3	9.631186	-17.3	4.898824	-15.2
13.01.2010	3.539309	-14.2	7.408599	-15.2	9.797660	-20.7
14.01.2010	3.539301	-15.8	9.631173	-17.0	11.430619	-10.2
15.01.2010	6.193762	-19.3	4.445154	-21.1	1.632948	-14.8
16.01.2010	6.193747	-21.4	0.740858	-25.3	9.797700	-24.4

Data	Chelyabinsk		Yekaterinburg		Barnaul	
	RI rates	Temperature	RI rates	Temperature	RI rates	Temperature
17.01.2010	8.848189	-14.4	5.186006	-15.8	1.632952	-31.1
18.01.2010	4.424084	-15.9	8.149432	-18.8	14.696590	-33.7
19.01.2010	12.387406	-23.9	5.926856	-25.6	9.797740	-32.8
20.01.2010	10.617751	-29.0	9.631134	-30.0	9.797753	-27.1
21.01.2010	7.078484	-29.3	6.667704	-29.6	8.164805	-24.6
22.01.2010	15.926552	-26.0	5.926844	-28.9	8.164816	-23.8
23.01.2010	10.617676	-23.1	8.149405	-29.1	13.063723	-23.8
24.01.2010	5.308825	-22.6	8.890254	-24.5	8.164838	-21.6
25.01.2010	5.308813	-19.5	4.445124	-19.9	8.164849	-17.6
26.01.2010	7.078400	-25.5	11.112803	-28.2	1.632972	-27.8
27.01.2010	10.617575	-30.4	9.631090	-28.9	11.430819	-30.8
28.01.2010	14.156734	-27.8	8.149378	-26.9	6.531906	-25.8
29.01.2010	14.156700	-18.2	5.926816	-19.8	9.797872	-22.5
30.01.2010	7.078333	-20.8	3.704258	-22.8	14.696827	-19.2
31.01.2010	7.078317	-10.9	5.185957	-13.6	8.164915	-23.0
01.02.2010	9.732662	-14.4	15.557862	-20.2	11.430896	-24.1
02.02.2010	17.695708	-22.3	6.667651	-22.4	14.696887	-24.8
03.02.2010	13.271750	-21.6	11.853593	-20.8	6.531958	-25.2
04.02.2010	8.847812	-18.8	19.262076	-18.1	6.531967	-27.6
05.02.2010	18.580361	-14.1	14.076123	-14.3	6.531976	-28.4
06.02.2010	12.386878	-18.2	8.890177	-20.1	13.063969	-27.7
07.02.2010	9.732524	-17.9	14.076105	-18.6	4.898995	-31.6
08.02.2010	12.386820	-25.6	6.667624	-25.8	4.899002	-29.9
09.02.2010	8.847707	-26.6	13.335239	-25.5	8.165014	-31.2
10.02.2010	10.617224	-25.8	16.298615	-23.5	9.798030	-30.0
11.02.2010	9.732432	-18.9	16.298604	-15.2	1.633007	-28.4
12.02.2010	14.156231	-12.9	15.557748	-12.2	6.532038	-26.8
13.02.2010	7.078099	-8.8	10.371825	-9.4	8.165058	-26.7
14.02.2010	12.386644	-8.4	14.076039	-8.8	11.431096	-16.3
15.02.2010	6.193307	-6.6	17.780248	-4.5	9.798096	-14.2
16.02.2010	14.156097	-7.2	20.002766	-5.7	3.266036	-11.1
17.02.2010	12.386556	-9.9	14.816854	-12.5	11.431143	-8.1
18.02.2010	12.386527	-15.1	12.594317	-19.2	4.899068	-4.5
19.02.2010	8.847498	-15.7	8.890100	-18.2	13.064198	-19.1
20.02.2010	4.423739	-16.5	8.149253	-20.6	8.165135	-15.4
21.02.2010	4.423728	-27.9	14.075973	-29.7	3.266058	-15.5
22.02.2010	7.962692	-25.8	13.335124	-27.7	11.431220	-27.7
23.02.2010	11.501639	-21.1	8.149237	-20.9	6.532134	-28.6
24.02.2010	20.349005	-19.1	6.667553	-16.3	6.532143	-27.1
25.02.2010	12.386322	-22.9	16.298452	-21.4	6.532152	-24.8
26.02.2010	10.616822	-21.9	10.371735	-22.8	9.798241	-22.1

**Data**	**Chelyabinsk**		**Yekaterinburg**		**Barnaul**	
	RI rates	Temperature	RI rates	Temperature	RI rates	Temperature
27.02.2010	15.925195	-9.7	13.335080	-11.1	4.899127	-16.6
28.02.2010	7.077848	-5.6	8.890047	-7.5	13.064357	-16.6
01.03.2010	9.732018	-17.3	8.890041	-17.3	4.899140	-14.2
02.03.2010	23.002896	-12.5	12.594217	-10.7	8.165245	-22.7
03.03.2010	21.233393	-11.7	14.075880	-5.7	4.899153	-21.1
04.03.2010	15.925007	-8.8	11.853365	-1.9	6.532213	-16.8
05.03.2010	23.002733	-7.8	10.371687	-4.4	8.165278	-17.9
06.03.2010	4.423592	-7.2	11.853349	-7.9	9.798347	-15.0
07.03.2010	13.270745	-9.8	12.594175	-10.9	11.431420	-12.1
08.03.2010	6.193000	-7.8	9.630833	-6.9	9.798373	-7.6
09.03.2010	17.694243	-6.1	15.557489	-7.4	21.229837	-2.3
10.03.2010	15.924781	-8.1	8.889988	-8.6	9.798399	-4.8
11.03.2010	10.616495	-9.4	14.075805	-9.2	4.899206	-8.9
12.03.2010	13.270588	-6.6	11.853301	-8.1	13.064568	-7.1
13.03.2010	7.077630	-3.7	9.630801	-2.8	16.330732	-9.2
14.03.2010	5.308210	-2.0	11.853286	-3.0	6.532301	-5.8
15.03.2010	12.385794	-2.3	7.408299	-3.7	9.798465	-3.4
16.03.2010	13.270462	-4.3	14.075758	-3.6	8.165399	-0.3
17.03.2010	15.039822	-5.2	7.408289	-5.0	3.266164	-0.7
18.03.2010	8.846933	-10.0	10.371597	-9.6	4.899252	-3.5
19.03.2010	14.155059	-8.0	11.112418	-8.7	1.633086	-5.2
20.03.2010	5.308135	-9.9	9.630756	-11.3	17.963974	-8.3
21.03.2010	9.731557	-11.8	7.408269	-11.0	8.165454	-10.2
22.03.2010	13.270274	-6.3	11.112396	-5.8	8.165465	-10.4
23.03.2010	9.731511	2.3	8.149085	-0.3	8.165476	-6.8
24.03.2010	10.616169	-2.2	5.926603	-4.1	4.899292	0.6
25.03.2010	10.616144	-2.3	8.149074	-3.1	14.697896	-2.6
26.03.2010	15.924178	-7.3	10.371542	-8.3	9.798611	0.9
27.03.2010	7.962070	-4.9	6.667415	-5.5	19.597247	-4.9
28.03.2010	7.962051	-0.6	11.853175	-0.6	8.165531	-7.0
29.03.2010	11.500714	1.1	12.593990	2.3	9.798650	-7.0
30.03.2010	7.077345	0.1	7.408225	4.9	8.165553	-7.4
31.03.2010	3.538664	3.7	5.926576	4.5	8.165564	-3.5
01.04.2010	7.077312	2.7	8.889858	2.3	8.165575	-0.1
02.04.2010	7.961957	2.7	5.926568	2.0	13.064937	-0.1
03.04.2010	3.538639	4.9	8.149025	3.9	1.633119	-0.6
04.04.2010	5.307946	6.7	9.630660	5.0	3.266243	0.3
05.04.2010	7.077245	7.4	14.075571	5.8	9.798742	0.8
06.04.2010	4.423268	-0.2	7.408190	-0.4	13.065008	-4.4
07.04.2010	8.846514	-2.9	4.444911	-1.5	13.065025	-8.3
08.04.2010	4.423247	1.7	13.334724	4.1	4.899391	-6.2

Data	Chelyabinsk		Yekaterinburg		Barnaul	
	RI rates	Temperature	RI rates	Temperature	RI rates	Temperature
09.04.2010	11.500414	4.2	9.630628	6.0	9.798795	-8.2
10.04.2010	3.538581	2.8	3.704085	5.1	6.532539	-6.0
11.04.2010	4.423215	1.2	8.889799	4.8	8.165685	-4.9
12.04.2010	13.269615	2.7	14.075505	5.6	1.633139	-1.8
13.04.2010	5.307833	2.4	8.889787	1.7	9.798848	1.6
14.04.2010	1.769274	1.2	9.630596	-0.3	9.798861	1.2
15.04.2010	2.653904	2.4	14.075477	1.9	8.165729	2.6
16.04.2010	5.307796	6.6	5.185699	5.2	14.698331	1.9
17.04.2010	1.769261	7.6	8.148949	6.0	4.899450	3.4
18.04.2010	1.769257	12.0	6.667318	9.6	6.532609	4.6
19.04.2010	7.961637	11.6	6.667313	8.9	6.532618	5.4
20.04.2010	2.653873	8.4	3.704061	7.5	3.266313	9.1
21.04.2010	6.192355	13.7	8.889739	11.2	11.432113	10.2
22.04.2010	5.307720	14.9	5.185678	14.9	8.165806	7.4
23.04.2010	3.538472	17.3	10.371349	18.6	11.432143	5.0
24.04.2010	6.192311	12.4	9.630532	10.6	4.899497	8.3
25.04.2010	6.192296	9.9	8.148906	9.2	3.266335	14.9
26.04.2010	1.769223	15.9	10.371328	12.1	6.532680	16.3
27.04.2010	11.499925	6.2	5.926469	3.9	8.165861	20.1
28.04.2010	4.423038	1.6	5.185657	1.2	3.266349	15.1
29.04.2010	5.307632	4.9	7.408077	1.7	4.899530	12.6
30.04.2010	5.307620	10.8	7.408072	7.5	1.633179	5.7
01.05.2010	5.307607	11.2	2.963227	8.4	1.633181	12.5
02.05.2010	2.653797	14.9	10.371287	13.6	4.899549	9.8
03.05.2010	7.076776	17.4	5.185640	15.8	8.165927	11.6
04.05.2010	4.422975	19.4	8.889662	16.4	9.799125	4.4
05.05.2010	14.153486	20.1	7.408047	17.5	6.532759	6.6
06.05.2010	7.961317	21.1	5.926434	19.5	6.532768	8.0
07.05.2010	6.192121	20.6	5.926430	15.6	6.532777	10.2
08.05.2010	2.653760	10.3	2.963213	7.9	3.266393	11.7
09.05.2010	1.769169	11.1	2.963211	12.1	3.266397	6.7
10.05.2010	7.076659	14.3	4.444813	13.4	4.899602	5.2
11.05.2010	3.538321	15.9	4.444810	15.8	4.899609	1.6
12.05.2010	0.884578	20.4	5.185609	20.3	3.266410	1.9
13.05.2010	4.422881	16.9	2.222402	18.6	16.332073	6.5
14.05.2010	4.422870	18.1	2.222401	18.7	3.266419	10.7
15.05.2010	1.769144	21.1	14.075196	20.6	3.266423	13.8
16.05.2010	2.653710	20.4	5.185595	17.7	1.633214	17.1
17.05.2010	4.422839	7.3	8.148787	4.5	4.899648	10.7
18.05.2010	2.653697	5.1	4.444790	5.9	6.532873	4.6
19.05.2010	7.961072	9.8	2.963191	7.8	4.899662	0.8

Data	Chelyabinsk		Yekaterinburg		Barnaul	
	RI rates	Temperature	RI rates	Temperature	RI rates	Temperature
20.05.2010	4.422807	4.2	2.963189	4.4	1.633223	3.7
21.05.2010	1.769119	11.8	5.185578	12.2	3.266450	3.9
22.05.2010	6.191901	17.7	5.926371	16.5	0.000000	7.1
23.05.2010	2.653666	12.8	7.407958	8.4	4.899688	9.6
24.05.2010	7.960978	14.2	6.667158	12.9	4.899695	11.1
25.05.2010	7.076408	18.6	5.185564	16.5	0.000000	13.9
26.05.2010	12.383685	21.7	9.630327	19.7	1.633236	19.0
27.05.2010	7.960921	16.7	7.407939	17.2	3.266476	22.4
28.05.2010	5.307268	14.3	6.667140	13.4	3.266481	19.1
29.05.2010	2.653628	11.1	8.889514	7.6	3.266485	19.7
30.05.2010	0.000000	6.9	3.703962	7.1	1.633245	20.3
31.05.2010	6.191769	14.1	5.185543	13.9	0.000000	13.3
01.06.2010	7.076291	15.9	3.703957	13.8	3.266498	10.0
02.06.2010	5.307206	19.4	2.963164	17.6	8.166257	16.2
03.06.2010	5.307193	22.4	5.926323	16.9	8.166268	18.1
04.06.2010	1.769060	22.3	7.407899	17.7	8.166279	16.5
05.06.2010	3.538112	27.0	2.963158	24.7	0.000000	15.1
06.06.2010	1.769052	22.2	5.185522	18.7	1.633260	16.7
07.06.2010	3.538095	14.1	4.444731	11.4	1.633262	21.7
08.06.2010	4.422609	17.3	5.926304	13.3	1.633265	24.6
09.06.2010	6.191637	18.2	5.185512	15.8	1.633267	20.5
10.06.2010	3.538070	13.8	5.185509	11.9	3.266538	18.1
11.06.2010	3.538062	11.4	1.481573	10.3	4.899813	21.7
12.06.2010	3.538053	17.1	1.481572	15.4	1.633273	16.7
13.06.2010	2.653534	22.7	3.703927	20.3	4.899827	16.7
14.06.2010	3.538037	25.6	2.963140	21.2	0.000000	20.8
15.06.2010	2.653521	20.4	3.703922	18.7	4.899840	27.6
16.06.2010	5.307030	21.4	3.703920	18.2	3.266564	21.9
17.06.2010	6.191520	26.3	2.963134	22.0	1.633284	17.8
18.06.2010	1.769002	23.1	0.740783	19.4	4.899860	20.5
19.06.2010	3.537995	16.2	0.740783	14.6	1.633289	25.1
20.06.2010	4.422483	14.8	2.963128	14.8	3.266582	27.8
21.06.2010	6.191462	16.6	3.703908	17.1	4.899879	20.5
22.06.2010	4.422462	20.1	3.703905	18.9	4.899886	11.9
23.06.2010	4.422452	22.4	2.963122	21.3	6.533190	12.4
24.06.2010	2.653465	24.7	5.926240	23.4	3.266599	15.0
25.06.2010	4.422431	22.3	2.963118	20.7	0.000000	15.3
26.06.2010	5.306904	20.8	2.963116	18.8	1.633304	16.2
27.06.2010	3.537928	20.2	2.963114	19.7	0.000000	17.6
28.06.2010	8.844799	24.4	2.963112	24.1	3.266617	13.8
29.06.2010	6.191345	25.1	0.740778	21.4	1.633311	14.3

Data	Chelyabinsk		Yekaterinburg		Barnaul	
	RI rates	Temperature	RI rates	Temperature	RI rates	Temperature
30.06.2010	7.075806	19.8	0.740777	18.4	4.899939	15.3
01.07.2010	0.884474	17.9	2.222330	15.7	4.899945	18.9
02.07.2010	4.422358	18.8	2.222328	17.6	0.000000	17.0
03.07.2010	0.884469	23.2	4.444654	21.7	3.266639	16.1
04.07.2010	2.653402	25.7	3.703876	20.8	0.000000	19.3
05.07.2010	7.075722	18.3	2.963099	16.8	3.266648	21.8
06.07.2010	1.768926	16.2	2.963097	16.4	1.633326	20.6
07.07.2010	6.191227	17.1	2.963095	17.4	4.899985	17.1
08.07.2010	5.306754	19.3	5.926185	17.9	0.000000	15.2
09.07.2010	2.653371	22.1	2.963091	21.3	1.633333	13.1
10.07.2010	1.768910	22.6	2.963089	21.0	1.633335	14.2
11.07.2010	2.653358	24.4	2.963087	23.2	1.633337	15.2
12.07.2010	1.768901	18.8	6.666941	19.8	1.633339	16.1
13.07.2010	2.653346	16.7	1.481541	15.8	8.166708	17.6
14.07.2010	5.306678	14.2	2.222311	14.3	4.900031	21.2
15.07.2010	0.000000	18.8	3.703848	19.8	1.633346	23.3
16.07.2010	6.191096	21.2	1.481538	21.4	0.000000	23.9
17.07.2010	4.422201	19.9	2.222306	20.2	4.900051	24.3
18.07.2010	0.884438	19.3	2.222305	18.4	6.533410	20.9
19.07.2010	3.537744	14.4	3.703839	13.8	6.533419	16.5
20.07.2010	2.653302	17.1	3.703836	16.9	1.633357	16.2
21.07.2010	7.959886	21.4	2.963067	20.2	0.000000	14.1
22.07.2010	11.497586	22.5	2.222299	21.1	1.633361	14.7
23.07.2010	3.537710	23.0	2.222297	22.6	0.000000	13.4
24.07.2010	2.653276	24.4	0.740765	24.7	1.633366	16.6
25.07.2010	5.306540	24.8	1.481530	24.8	1.633368	20.6
26.07.2010	9.728634	19.0	3.703821	18.2	0.000000	20.7
27.07.2010	7.959773	19.2	5.185346	17.2	1.633372	10.4
28.07.2010	6.190920	21.1	2.222290	18.7	3.266749	10.1
29.07.2010	5.306490	20.7	4.444577	20.9	1.633377	11.6
30.07.2010	4.422065	23.9	7.407623	25.1	0.000000	15.2
31.07.2010	5.306465	25.7	4.444571	25.9	0.000000	16.9
01.08.2010	3.537635	25.6	7.407613	25.3	0.000000	14.9
02.08.2010	6.190847	26.0	2.963043	23.7	3.266771	13.8
03.08.2010	8.844046	25.8	2.222281	23.8	3.266775	17.1
04.08.2010	4.422012	24.3	5.185319	23.1	3.266780	19.1
05.08.2010	7.959604	23.2	5.185315	22.5	0.000000	19.8
06.08.2010	7.959585	25.4	5.185312	23.7	0.000000	17.4
07.08.2010	3.537585	27.2	3.703792	25.3	0.000000	21.4
08.08.2010	5.306365	22.4	3.703789	20.5	1.633399	21.8
09.08.2010	3.537568	18.1	3.703787	16.9	0.000000	15.7

Data	Chelyabinsk		Yekaterinburg		Barnaul	
	RI rates	Temperature	RI rates	Temperature	RI rates	Temperature
10.08.2010	2.653170	22.9	2.222271	23.1	3.266806	14.3
11.08.2010	4.421939	28.4	3.703782	26.9	0.000000	11.3
12.08.2010	7.959472	28.6	2.963024	25.8	3.266815	15.1
13.08.2010	4.421918	22.4	2.222266	22.3	4.900229	17.9
14.08.2010	4.421908	18.1	4.444529	15.6	1.633412	18.7
15.08.2010	3.537518	16.2	2.963018	14.8	6.533656	13.6
16.08.2010	5.306264	20.4	3.703770	19.5	8.167082	12.3
17.08.2010	8.843753	15.1	0.740753	14.1	4.900256	15.3
18.08.2010	1.768746	14.3	2.963012	13.2	0.000000	17.9
19.08.2010	7.074969	20.4	2.963010	16.2	3.266846	13.6
20.08.2010	4.421845	18.2	7.407519	12.4	3.266850	19.1
21.08.2010	3.537468	10.4	2.963006	7.1	3.266855	23.3
22.08.2010	5.306189	9.5	4.444506	8.2	0.000000	25.1
23.08.2010	6.190539	12.8	4.444503	8.9	11.434022	17.4
24.08.2010	4.421803	16.2	4.444500	13.2	1.633434	12.9
25.08.2010	6.190510	15.1	6.666745	10.8	1.633436	15.4
26.08.2010	1.768713	16.0	6.666741	15.8	0.000000	19.2
27.08.2010	3.537418	22.8	6.666736	19.5	1.633441	18.5
28.08.2010	5.306114	16.6	4.444488	14.6	1.633443	20.4
29.08.2010	0.000000	20.6	5.925980	14.9	3.266890	22.2
30.08.2010	9.727829	26.9	8.148217	23.4	4.900341	21.0
31.08.2010	5.306076	18.4	5.925972	15.6	0.000000	22.0
01.09.2010	2.653032	9.4	2.222238	8.0	1.633452	24.9
02.09.2010	4.421709	10.8	1.481491	9.9	3.266907	13.6
03.09.2010	2.653019	16.2	6.666705	16.4	0.000000	7.4
04.09.2010	2.653013	20.3	3.703723	17.7	1.633458	9.6
05.09.2010	5.306013	17.6	2.962976	13.8	4.900381	15.4
06.09.2010	5.306001	13.7	2.962974	11.0	3.266925	19.9
07.09.2010	3.537326	6.9	2.962972	6.7	0.000000	19.4
08.09.2010	6.190305	8.1	2.222228	7.0	0.000000	21.3
09.09.2010	2.652982	8.4	2.962968	7.4	3.266938	10.3
10.09.2010	1.768650	5.3	1.481483	5.3	0.000000	10.6
11.09.2010	1.768646	4.1	2.222223	3.2	0.000000	7.5
12.09.2010	0.000000	2.9	2.962962	2.5	1.633476	3.6
13.09.2010	3.537275	5.4	4.444441	4.2	3.266956	4.9
14.09.2010	2.652950	7.1	0.000000	6.5	3.266960	7.8
15.09.2010	7.074517	12.4	0.740739	10.9	1.633482	5.1
16.09.2010	5.305875	14.9	2.962955	13.6	1.633485	9.7
17.09.2010	4.421552	16.1	2.962953	14.7	0.000000	9.7
18.09.2010	1.768617	16.9	2.222213	14.3	0.000000	4.3
19.09.2010	0.000000	17.4	2.962949	15.6	1.633491	3.3

Data	Chelyabinsk		Yekaterinburg		Barnaul	
	RI rates	Temperature	RI rates	Temperature	RI rates	Temperature
20.09.2010	0.000000	17.1	4.444420	15.7	0.000000	7.8
21.09.2010	3.537208	19.4	5.185153	17.2	4.900487	13.2
22.09.2010	7.074400	13.1	4.444414	10.6	1.633498	15.2
23.09.2010	0.884298	12.8	2.962941	12.7	0.000000	9.8
24.09.2010	1.768592	16.1	7.407347	13.3	3.267004	9.3
25.09.2010	1.768587	13.4	2.962937	10.9	1.633504	9.7
26.09.2010	0.884292	10.8	4.444402	8.1	8.167533	12.2
27.09.2010	2.652869	12.3	5.185132	10.3	6.534035	14.7
28.09.2010	5.305725	8.4	2.962931	5.7	3.267022	11.0
29.09.2010	3.537142	14.1	2.962929	9.6	0.000000	8.7
30.09.2010	2.652850	11.3	2.962927	7.3	3.267031	12.8
01.10.2010	1.768562	2.1	3.703656	1.2	0.000000	16.8
02.10.2010	3.537116	0.9	3.703654	-0.7	0.000000	18.8
03.10.2010	0.000000	2.7	3.703651	2.7	1.633522	11.8
04.10.2010	5.305650	3.8	2.222189	2.0	0.000000	4.7
05.10.2010	5.305637	3.3	5.925834	3.7	1.633526	2.7
06.10.2010	7.074166	4.1	1.481458	3.4	3.267057	2.9
07.10.2010	1.768537	6.6	2.222185	6.6	1.633531	3.1
08.10.2010	3.537066	6.7	1.481456	5.4	3.267066	3.3
09.10.2010	2.652793	7.4	5.185091	5.6	0.000000	5.4
10.10.2010	2.652787	6.1	2.962907	4.1	0.000000	6.4
11.10.2010	3.537041	7.2	3.703632	3.9	1.633540	7.7
12.10.2010	2.652775	8.7	3.703629	8.9	1.633542	8.4
13.10.2010	4.421281	6.9	2.962901	5.3	1.633544	8.2
14.10.2010	5.305524	4.3	7.407248	2.9	4.900638	9.2
15.10.2010	2.652756	3.6	5.185070	1.7	3.267097	10.3
16.10.2010	5.305499	4.7	0.740724	3.6	1.633551	4.3
17.10.2010	0.884248	2.4	1.481447	2.1	1.633553	5.1
18.10.2010	5.305474	0.5	2.962891	-0.3	1.633555	6.1
19.10.2010	8.842436	0.9	4.444334	-0.1	0.000000	1.6
20.10.2010	7.073932	3.1	5.925775	2.9	3.267119	0.1
21.10.2010	2.652718	2.2	3.703607	1.2	3.267123	0.3
22.10.2010	8.842373	6.8	3.703604	6.3	1.633564	2.1
23.10.2010	1.768470	6.9	5.185043	6.1	4.900698	1.7
24.10.2010	4.421166	3.4	2.222160	3.3	3.267136	1.7
25.10.2010	3.536924	0.9	4.444316	0.1	4.900711	2.1
26.10.2010	5.305374	2.1	5.185032	1.3	0.000000	3.7
27.10.2010	8.842268	2.2	2.222155	3.6	3.267150	4.3
28.10.2010	5.305348	3.6	2.962872	4.0	6.534308	4.7
29.10.2010	3.536891	5.6	2.962870	6.0	3.267158	3.8
30.10.2010	7.957985	1.9	2.962868	2.2	0.000000	4.8

Data	Chelyabinsk		Yekaterinburg		Barnaul	
	RI rates	Temperature	RI rates	Temperature	RI rates	Temperature
31.10.2010	7.073748	0.0	3.703582	-1.3	0.000000	7.2
01.11.2010	6.189515	-3.0	2.962864	-2.7	0.000000	5.9
02.11.2010	4.421071	1.3	2.222146	-0.9	3.267176	8.6
03.11.2010	5.305273	1.6	3.703575	1.2	4.900770	4.6
04.11.2010	4.421051	1.9	2.962858	3.2	3.267185	1.2
05.11.2010	2.652624	7.1	1.481428	4.6	1.633595	2.6
06.11.2010	5.305236	2.9	7.407135	2.0	1.633597	3.3
07.11.2010	7.073631	0.2	1.481426	-0.8	3.267198	5.3
08.11.2010	3.536807	0.0	5.184987	0.1	3.267202	2.9
09.11.2010	0.884200	0.9	2.962848	2.4	1.633603	-0.3
10.11.2010	6.189383	2.4	2.962846	6.2	3.267211	0.8
11.11.2010	5.305173	2.2	2.222133	6.8	6.534431	-4.2
12.11.2010	5.305160	2.9	2.962842	6.1	3.267220	-4.1
13.11.2010	2.652574	4.9	7.407100	5.9	3.267224	2.3
14.11.2010	2.652568	6.6	2.962838	5.5	4.900843	6.7
15.11.2010	3.536748	5.0	7.407090	4.2	3.267233	5.3
16.11.2010	7.957665	4.9	4.444251	4.4	3.267238	2.1
17.11.2010	10.610195	4.7	8.888497	3.7	0.000000	0.8
18.11.2010	8.841808	-2.6	5.925661	-3.2	9.801739	-2.6
19.11.2010	6.189251	-2.1	4.444242	-2.6	3.267251	-6.1
20.11.2010	0.884177	-1.5	3.703533	-4.5	4.900883	-2.9
21.11.2010	2.652524	-12.4	7.407061	-15.5	4.900889	-6.6
22.11.2010	7.957552	-11.2	4.444234	-12.6	1.633632	-15.1
23.11.2010	6.189193	-7.2	3.703526	-11.1	4.900903	-13.9
24.11.2010	11.494188	-4.9	5.925637	-8.9	4.900909	-7.7
25.11.2010	6.189163	-3.4	5.925633	-11.2	0.000000	-10.5
26.11.2010	10.609969	-1.4	7.407036	-11.6	6.534563	-4.9
27.11.2010	5.304972	-3.8	7.407031	-14.6	4.900929	-0.8
28.11.2010	2.652480	-11.4	6.666324	-16.6	4.900936	-4.0
29.11.2010	7.073263	-19.1	2.962809	-22.4	1.633647	0.1
30.11.2010	8.841558	-18.7	2.962807	-25.9	8.168248	-13.1
01.12.2010	5.304922	-18.4	5.925609	-22.1	4.900955	-12.7
02.12.2010	4.420758	-22.9	8.147707	-24.7	6.534616	-6.0
03.12.2010	0.884149	-13.9	5.184901	-17.0	4.900969	-20.9
04.12.2010	0.884147	-10.6	2.222099	-13.3	3.267317	-16.5
05.12.2010	1.768291	-2.4	6.666293	-2.4	3.267321	-11.1
06.12.2010	7.957289	-2.6	8.147686	-3.5	4.900988	-6.2
07.12.2010	6.188988	-4.6	5.184887	-7.9	1.633665	-3.6
08.12.2010	5.304834	-9.1	8.888372	-11.4	3.267334	-9.4
09.12.2010	8.841369	-3.3	5.925578	-5.5	3.267339	-21.9
10.12.2010	9.725483	-3.3	8.888361	-5.8	1.633672	-15.5

Data	Chelyabinsk		Yekaterinburg		Barnaul	
	RI rates	Temperature	RI rates	Temperature	RI rates	Temperature
11.12.2010	4.420664	-5.6	9.629051	-4.0	4.901021	-23.9
12.12.2010	7.957176	-12.8	8.147653	-10.7	1.633676	-26.9
13.12.2010	6.188900	-14.6	5.925562	-16.1	1.633678	-21.8
14.12.2010	6.188885	-8.6	5.925558	-12.7	6.534722	-19.5
15.12.2010	12.377742	-9.7	11.110414	-7.5	1.633683	-13.7
16.12.2010	8.841223	-6.8	3.703469	-3.0	1.633685	-11.7
17.12.2010	13.261803	-5.7	7.406933	-8.7	4.901061	-6.7
18.12.2010	2.652354	-20.7	10.369699	-23.5	3.267378	-7.3
19.12.2010	8.841160	-26.1	7.406923	-29.4	6.534766	-25.3
20.12.2010	6.188798	-29.3	5.925534	-31.8	3.267387	-31.2
21.12.2010	11.493454	-30.0	8.888296	-30.3	4.901087	-32.1
22.12.2010	11.493427	-13.4	8.147599	-13.7	1.633698	-34.2
23.12.2010	7.956969	-12.3	8.147593	-12.3	11.435901	-26.2
24.12.2010	7.956950	-15.3	11.851037	-17.6	3.267405	-8.4
25.12.2010	7.956932	-18.0	9.628961	-20.8	0.000000	-21.4
26.12.2010	5.304608	-16.6	4.444133	-24.0	0.000000	-21.7
27.12.2010	8.840993	-19.6	6.666195	-23.6	3.267418	-21.9
28.12.2010	3.536389	-12.8	7.406878	-15.6	1.633711	-25.1
29.12.2010	5.304571	-15.1	6.666186	-16.8	1.633713	-19.4
30.12.2010	2.652279	-18.8	6.666182	-23.7	1.633716	-17.2
31.12.2010	3.536364	-23.1	2.222059	-18.8	0.000000	-24.3
01.01.2011	1.768178	-17.8	5.184801	-12.8	0.000000	-28.3
02.01.2011	2.652187	-21.9	3.703222	-19.2	1.633650	-32.3
03.01.2011	3.536144	-20.3	0.740603	-18.2	0.000000	-32.8
04.01.2011	4.420048	-21.4	2.221684	-19.1	0.000000	-33.5
05.01.2011	10.607797	-25.6	2.962079	-21.9	0.000000	-33.1
06.01.2011	4.419783	-26.9	1.480956	-21.6	0.000000	-32.5
07.01.2011	9.723232	-20.4	2.961747	-25.1	1.633300	-30.3
08.01.2011	11.490749	-21.8	5.182766	-25.2	0.000000	-28.3
09.01.2011	7.954896	-20.6	2.221061	-18.1	0.000000	-26.8
10.01.2011	9.722360	-9.0	2.961249	-8.9	0.000000	-24.1
11.01.2011	15.025016	-2.5	5.922166	-5.8	3.266041	-19.6
12.01.2011	9.721779	-6.8	8.882750	-9.4	1.632951	-20.9
13.01.2011	14.140346	-11.4	10.362628	-10.5	1.632881	-14.1
14.01.2011	10.604942	-19.1	12.582486	-15.7	4.898433	-11.4
15.01.2011	9.720907	-22.6	8.881257	-21.9	0.000000	-14.3
16.01.2011	13.255385	-24.2	14.801266	-23.3	4.898013	-15.4
17.01.2011	16.789653	-20.4	14.060414	-20.9	16.326012	-16.9
18.01.2011	14.138232	-18.2	11.839685	-21.9	16.325313	-22.1
19.01.2011	12.370583	-23.6	8.879267	-22.9	8.162307	-25.1
20.01.2011	5.301520	-27.6	7.398974	-25.9	13.059133	-25.2

Data	Chelyabinsk		Yekaterinburg		Barnaul	
	RI rates	Temperature	RI rates	Temperature	RI rates	Temperature
21.01.2011	25.623248	-23.3	8.878272	-24.4	9.793931	-25.1
22.01.2011	13.253008	-23.2	4.438887	-23.7	0.000000	-23.1
23.01.2011	8.835074	-25.5	10.356823	-24.8	6.528728	-22.5
24.01.2011	27.387912	-24.3	18.493291	-23.8	9.792673	-22.2
25.01.2011	15.902183	-13.5	11.835044	-13.7	14.688382	-23.1
26.01.2011	20.318849	-9.8	10.355083	-13.6	14.687753	-22.3
27.01.2011	15.017831	-15.8	14.792147	-10.4	8.159514	-14.8
28.01.2011	22.967760	-12.9	10.353923	-11.6	14.686496	-13.1
29.01.2011	10.600188	-13.6	15.530015	-13.4	6.527052	-11.8
30.01.2011	14.133162	-8.4	10.352764	-9.1	1.631693	-6.9
31.01.2011	14.132739	-5.2	20.704368	-7.3	8.158118	-10.4
01.02.2011	19.431936	-13.2	21.442609	-16.4	6.526215	-10.2
02.02.2011	12.365408	-15.6	15.526538	-21.7	1.631484	-10.8
03.02.2011	17.664340	-10.3	11.829081	-10.7	9.788485	-5.8
04.02.2011	13.247859	-9.8	11.828418	-10.2	8.156722	-13.3
05.02.2011	14.130628	-10.1	17.002400	-9.2	16.312746	-11.1
06.02.2011	11.480792	-10.7	9.609514	-9.9	1.631205	-11.1
07.02.2011	12.363560	-6.7	12.565584	-7.9	6.524540	-14.1
08.02.2011	15.012446	-7.7	15.521324	-11.3	6.524261	-11.4
09.02.2011	13.245880	-15.4	11.825109	-18.1	9.785974	-5.9
10.02.2011	9.713355	-12.8	14.780559	-15.1	6.523704	-10.9
11.02.2011	9.713065	-13.6	12.562772	-16.4	3.261712	-9.5
12.02.2011	11.478734	-17.3	15.517850	-20.1	6.523146	-8.2
13.02.2011	7.946578	-17.2	13.300270	-20.0	8.153584	-10.7
14.02.2011	17.658535	-18.8	16.993839	-18.4	0.000000	-14.5
15.02.2011	6.180303	-17.3	11.821139	-19.5	3.261155	-11.9
16.02.2011	7.945866	-14.4	10.342918	-17.2	4.891523	-5.4
17.02.2011	10.594172	-19.6	12.558555	-21.5	1.630438	-2.3
18.02.2011	16.773604	-23.1	17.728734	-25.4	14.673314	-11.2
19.02.2011	12.359129	-23.6	12.557151	-27.9	8.151493	-16.1
20.02.2011	6.179380	-26.0	16.988136	-30.9	6.520915	-17.0
21.02.2011	8.827422	-25.9	19.202906	-29.2	11.411114	-21.9
22.02.2011	14.123453	-23.4	8.862384	-21.2	9.780537	-24.1
23.02.2011	16.771099	-15.7	12.554342	-13.4	8.150099	-22.5
24.02.2011	15.887935	-12.2	17.722786	-13.2	1.629950	-20.0
25.02.2011	12.356914	-14.1	13.291346	-14.3	6.519522	-20.4
26.02.2011	10.591324	-16.1	14.767337	-16.4	6.519244	-18.6
27.02.2011	9.708424	-14.9	15.504837	-16.2	6.518965	-20.1
28.02.2011	16.768595	-13.4	14.027401	-14.0	6.518687	-17.9
01.03.2011	15.885563	-12.9	18.456075	-15.2	11.407214	-16.2
02.03.2011	20.297614	-13.4	16.240438	-13.4	0.000000	-15.9

Data	Chelyabinsk		Yekaterinburg		Barnaul	
	RI rates	Temperature	RI rates	Temperature	RI rates	Temperature
03.03.2011	16.767093	-10.6	23.621135	-10.9	3.258926	-15.6
04.03.2011	19.413949	-16.3	17.714861	-10.8	4.888180	-14.5
05.03.2011	10.589111	-16.4	17.713871	-11.6	1.629324	-12.1
06.03.2011	5.294397	-7.3	13.284661	-7.9	14.663286	-8.2
07.03.2011	5.294239	-4.2	12.545923	-5.6	9.775106	-4.2
08.03.2011	13.235203	-7.4	14.759085	-11.7	8.145574	-2.6
09.03.2011	20.293372	-8.3	18.447825	-7.4	9.774272	-1.2
10.03.2011	11.469824	-7.1	13.281692	-6.2	8.144878	-10.0
11.03.2011	10.587214	-6.3	11.805289	-4.4	4.886718	-14.1
12.03.2011	6.175690	-9.1	11.804629	-6.1	3.257673	-13.3
13.03.2011	19.408734	-6.6	20.656948	-6.2	6.515068	-8.7
14.03.2011	17.643777	-5.4	19.180380	-5.9	8.143487	-4.7
15.03.2011	15.878925	-3.1	16.228646	-3.6	11.400395	-1.4
16.03.2011	9.703498	-0.4	20.653486	-1.4	3.257117	-5.6
17.03.2011	12.349538	-4.7	12.538916	-7.9	1.628489	-6.1
18.03.2011	8.820835	-9.6	22.126264	-9.6	8.142097	-11.4
19.03.2011	6.174400	-13.0	15.487520	-9.4	13.026799	-10.6
20.03.2011	5.292185	-13.0	7.374598	-8.1	4.884841	-8.7
21.03.2011	13.230068	-10.0	31.708999	-5.4	11.397476	-6.3
22.03.2011	12.347695	-9.7	12.535416	-5.8	3.256283	-4.6
23.03.2011	14.993182	-4.2	16.958733	-0.3	9.768431	-5.1
24.03.2011	19.402363	-1.6	16.220491	1.3	3.256005	-1.9
25.03.2011	11.464690	-1.4	11.058809	-1.1	14.651396	2.7
26.03.2011	10.582475	-4.9	11.795404	-4.6	8.139317	2.6
27.03.2011	11.464006	-6.9	11.057574	-6.9	8.138970	-1.3
28.03.2011	14.990945	-5.7	10.319826	-6.2	4.883173	0.1
29.03.2011	12.345116	-6.9	10.319250	-5.1	6.510620	-5.7
30.03.2011	9.699445	-8.7	14.740964	-6.6	3.255171	-5.0
31.03.2011	7.053931	-3.7	10.318099	-3.7	17.902677	-2.9
01.04.2011	14.989157	-2.1	12.528421	-2.9	9.764680	3.8
02.04.2011	7.053510	-4.1	9.580022	-5.3	8.136886	2.4
03.04.2011	3.526650	-0.5	12.527023	-0.7	4.881923	-2.2
04.04.2011	12.342907	3.4	12.526324	3.7	4.881715	-4.3
05.04.2011	11.460929	5.2	13.262426	4.9	4.881507	0.3
06.04.2011	8.815836	5.9	2.947041	4.8	3.254199	2.2
07.04.2011	7.052458	3.3	0.000000	4.3	3.254060	3.3
08.04.2011	9.696841	4.0	0.000000	4.7	3.253921	2.9
09.04.2011	10.578056	4.8	0.000000	4.1	4.880674	5.5
10.04.2011	6.170349	3.9	0.000000	3.6	13.014576	9.9
11.04.2011	14.984686	7.0	0.000000	5.1	8.133763	11.6
12.04.2011	15.865665	6.9	0.000000	5.6	3.253366	14.9

Data	Chelyabinsk		Yekaterinburg		Barnaul	
	RI rates	Temperature	RI rates	Temperature	RI rates	Temperature
13.04.2011	12.339594	5.9	0.000000	6.2	6.506455	14.6
14.04.2011	9.695106	8.4	0.000000	6.8	4.879633	14.0
15.04.2011	7.932123	7.9	0.736391	5.9	8.132376	14.9
16.04.2011	2.643962	7.7	0.000000	5.7	4.879217	16.9
17.04.2011	7.050355	11.5	0.000000	10.0	9.758019	9.4
18.04.2011	9.693950	7.4	0.000000	5.8	4.878801	10.6
19.04.2011	12.337386	5.1	0.000000	3.5	1.626198	14.7
20.04.2011	5.287294	5.5	0.000000	3.4	6.504514	12.4
21.04.2011	6.168325	5.7	0.000000	3.7	8.130296	14.4
22.04.2011	7.930467	1.9	0.736103	1.4	4.877969	20.9
23.04.2011	4.405684	4.9	0.000000	3.9	4.877761	8.7
24.04.2011	6.167774	6.7	0.000000	5.3	9.755107	5.2
25.04.2011	5.286505	4.6	0.000000	2.1	3.251564	9.1
26.04.2011	6.167406	4.2	0.000000	2.3	3.251425	8.9
27.04.2011	8.810317	8.9	0.735898	7.8	3.251287	6.4
28.04.2011	7.929049	13.2	0.000000	11.6	3.251148	3.9
29.04.2011	7.047833	13.3	0.000000	11.6	8.127524	7.7
30.04.2011	7.047623	9.9	1.471550	7.7	6.501742	11.2
01.05.2011	9.690193	15.4	5.150140	10.6	4.876099	12.8
02.05.2011	2.642701	14.1	6.621239	9.9	0.000000	8.5
03.05.2011	9.689616	10.5	8.092175	7.9	3.250455	6.6
04.05.2011	4.404239	12.3	3.678056	11.2	3.250317	7.5
05.05.2011	5.284930	10.3	5.884562	9.4	8.125446	4.4
06.05.2011	4.403977	10.1	14.710587	9.5	8.125100	6.2
07.05.2011	4.403846	13.8	8.825861	12.9	1.624951	11.4
08.05.2011	5.284457	13.7	8.089922	12.9	9.749289	2.7
09.05.2011	4.403583	16.1	11.766503	16.3	3.249625	2.1
10.05.2011	1.761381	19.1	4.412193	17.3	4.874229	9.5
11.05.2011	6.164649	11.7	8.088570	7.4	11.372717	15.7
12.05.2011	6.164465	4.5	10.293971	3.2	3.249209	19.1
13.05.2011	8.806117	6.7	6.617184	4.6	4.873607	16.9
14.05.2011	7.044684	7.1	6.616816	4.5	1.624466	11.7
15.05.2011	6.163914	8.3	8.086769	6.3	1.624397	12.6
16.05.2011	6.163731	11.3	6.616079	9.2	9.745968	7.8
17.05.2011	8.805067	14.2	2.940316	12.4	3.248518	7.5
18.05.2011	5.282883	16.1	5.880305	16.7	4.872569	11.4
19.05.2011	4.402271	17.4	7.349972	15.2	8.120603	14.7
20.05.2011	7.923852	11.7	7.349563	8.7	8.120257	19.8
21.05.2011	7.043215	16.3	6.614238	14.4	4.871947	15.3
22.05.2011	4.401878	17.3	6.613870	14.1	4.871739	14.4
23.05.2011	7.042795	14.8	5.878669	12.8	6.495376	13.9

Data	Chelyabinsk		Yekaterinburg		Barnaul	
	RI rates	Temperature	RI rates	Temperature	RI rates	Temperature
24.05.2011	6.162262	7.1	9.552305	7.1	4.871324	15.5
25.05.2011	4.401485	12.4	2.939007	12.9	0.000000	19.6
26.05.2011	3.521083	17.5	5.877688	16.0	1.623637	18.3
27.05.2011	5.281467	19.0	6.612031	18.9	1.623567	11.9
28.05.2011	3.520873	11.1	3.673146	14.8	3.246997	13.9
29.05.2011	0.880192	11.8	8.080472	12.8	0.000000	17.5
30.05.2011	7.041327	16.7	3.672737	17.4	3.246720	22.9
31.05.2011	8.801396	15.8	5.876053	13.4	4.869873	21.8
01.06.2011	7.921021	12.8	9.548055	13.8	1.623222	15.8
02.06.2011	7.040698	15.2	6.609825	14.0	4.869459	21.0
03.06.2011	6.160427	15.8	6.609457	17.2	4.869252	20.8
04.06.2011	5.280209	17.2	4.406060	16.8	3.246030	19.3
05.06.2011	4.400043	20.1	0.734302	19.5	4.868837	20.1
06.06.2011	3.519930	19.7	5.874093	19.4	4.868630	18.1
07.06.2011	6.159693	17.6	2.202662	18.0	3.245615	21.6
08.06.2011	7.919370	16.8	2.202540	16.1	4.868216	23.3
09.06.2011	2.639711	18.1	0.734139	17.9	3.245339	22.8
10.06.2011	2.639633	20.3	2.202295	16.2	3.245201	22.9
11.06.2011	3.519406	19.3	2.202173	18.7	3.245063	22.7
12.06.2011	0.879825	13.3	3.670084	10.8	6.489850	20.9
13.06.2011	1.759598	9.6	1.467952	8.4	4.867181	22.5
14.06.2011	7.917956	11.0	2.201805	9.4	1.622325	20.2
15.06.2011	4.398733	15.9	2.935578	13.5	0.000000	18.1
16.06.2011	3.518882	18.6	2.935414	14.8	1.622187	18.7
17.06.2011	1.759389	18.3	4.402877	16.3	1.622118	21.4
18.06.2011	0.879668	14.3	1.467544	15.4	1.622049	23.7
19.06.2011	1.759284	14.3	1.467463	13.3	4.865939	24.6
20.06.2011	1.759231	14.7	5.135834	13.4	1.621911	20.9
21.06.2011	4.397948	16.1	3.668249	15.5	4.865525	19.0
22.06.2011	0.879563	15.3	2.934436	16.8	1.621773	15.2
23.06.2011	1.759074	15.7	2.200705	16.5	3.243408	15.9
24.06.2011	1.759022	17.0	0.733528	17.7	3.243270	20.3
25.06.2011	1.758970	17.9	0.733487	18.2	27.566620	17.6
26.06.2011	1.758917	16.6	2.200338	17.4	0.000000	16.6
27.06.2011	4.397163	18.8	0.733405	19.7	3.242856	18.2
28.06.2011	6.155844	20.9	2.933458	21.2	3.242718	20.1
29.06.2011	0.000000	22.7	1.466648	22.8	3.242580	23.3
30.06.2011	2.638062	18.1	1.466566	17.9	0.000000	25.2
01.07.2011			2.932970	19.6	6.484609	20.2
02.07.2011			4.399210	20.2	3.242167	14.7
03.07.2011			2.932644	22.7	1.621015	12.3

Data	Chelyabinsk		Yekaterinburg		Barnaul	
	RI rates	Temperature	RI rates	Temperature	RI rates	Temperature
04.07.2011			2.199361	24.6	6.483783	13.6
05.07.2011			2.199239	17.1	6.483507	18.2
06.07.2011			2.932156	17.5	4.862424	19.7
07.07.2011			2.931993	21.8	3.241478	15.1
08.07.2011			1.465915	23.2	0.000000	17.1
09.07.2011			1.465834	15.9	3.241203	16.2
10.07.2011			0.000000	13.4	3.241065	17.9
11.07.2011			2.931342	12.3	6.481855	21.8
12.07.2011			1.465590	11.8	0.000000	22.7
13.07.2011			2.931017	15.8	3.240652	21.4
14.07.2011			2.930854	17.3	4.860771	17.9
15.07.2011			3.663365	18.8	1.620188	19.9
16.07.2011			0.732632	16.3	3.240239	17.7
17.07.2011			4.395550	18.8	3.240101	21.0
18.07.2011			1.465102	22.1	1.619982	21.2
19.07.2011			2.197531	18.8	0.000000	22.2
20.07.2011			1.464939	16.7	3.239689	22.3
21.07.2011			3.662146	15.3	0.000000	16.4
22.07.2011			1.464777	19.0	3.239414	15.3
23.07.2011			2.929392	23.6	1.619638	16.8
24.07.2011			1.464615	23.8	0.000000	19.4
25.07.2011			0.000000	23.2	3.239001	19.7
26.07.2011			1.464452	25.1	3.238863	17.3
27.07.2011			3.660927	23.1	1.619363	19.1
28.07.2011			3.660724	21.1	0.000000	16.2
29.07.2011			3.660521	20.6	3.238451	13.5
30.07.2011			2.928255	15.3	1.619157	16.2
31.07.2011			1.464046	12.7	0.000000	21.4
01.08.2011					3.238039	25.3
02.08.2011					9.713703	20.8
03.08.2011					3.237764	19.8
04.08.2011					3.237626	15.6
05.08.2011					6.474978	13.4
06.08.2011					3.237351	12.7
07.08.2011					1.618607	15.1
08.08.2011					3.237077	14.8
09.08.2011					6.473879	14.7
10.08.2011					1.618401	18.4
11.08.2011					1.618332	17.3
12.08.2011					1.618264	17.4
13.08.2011					1.618195	19.4

Data	Chelyabinsk		Yekaterinburg		Barnaul	
	RI rates	Temperature	RI rates	Temperature	RI rates	Temperature
14.08.2011					0.000000	20.4
15.08.2011					0.000000	21.9
16.08.2011					1.617989	23.9
17.08.2011					3.235841	18.2
18.08.2011					1.617852	13.4
19.08.2011					1.617783	12.7
20.08.2011					1.617715	10.5
21.08.2011					4.852938	14.2
22.08.2011					1.617577	14.5
23.08.2011					3.235018	16.2
24.08.2011					0.000000	20.6
25.08.2011					1.617372	15.2
26.08.2011					3.234606	11.9
27.08.2011					3.234469	10.4
28.08.2011					3.234332	11.1
29.08.2011					8.085487	12.8
30.08.2011					0.000000	15.3
31.08.2011					0.000000	15.6
01.09.2011					1.616892	18.1
02.09.2011					3.233646	8.9
03.09.2011					1.616755	15.6
04.09.2011					1.616686	11.7
05.09.2011					3.233235	11.0
06.09.2011					0.000000	10.9
07.09.2011					1.616481	12.1
08.09.2011					3.232824	15.7
09.09.2011					0.000000	19.1
10.09.2011					6.465101	12.4
11.09.2011					1.616207	8.4
12.09.2011					0.000000	9.1
13.09.2011					1.616070	10.3
14.09.2011					0.000000	10.4
15.09.2011					1.615933	12.1
16.09.2011					0.000000	13.1
17.09.2011					1.615796	15.2
18.09.2011					4.847182	16.2
19.09.2011					1.615659	17.1
20.09.2011					3.231181	19.3
21.09.2011					3.231044	17.2
22.09.2011					3.230907	11.8
23.09.2011					0.000000	12.3

Data	Chelyabinsk		Yekaterinburg		Barnaul	
	RI rates	Temperature	RI rates	Temperature	RI rates	Temperature
24.09.2011					3.230634	9.2
25.09.2011					1.615249	4.9
26.09.2011					0.000000	7.3
27.09.2011					3.230223	9.1
28.09.2011					4.845130	9.9
29.09.2011					9.689850	11.4
30.09.2011					3.229813	10.6
01.10.2011					3.229676	11.8
02.10.2011					1.614770	14.7
03.10.2011					1.614702	15.1
04.10.2011					6.458533	11.9
05.10.2011					1.614565	12.2
06.10.2011					1.614496	8.3
07.10.2011					4.843284	9.0
08.10.2011					3.228720	11.3
09.10.2011					3.228583	5.4
10.10.2011					1.614223	4.8
11.10.2011					4.842465	8.4
12.10.2011					0.000000	11.7
13.10.2011					1.614018	12.6
14.10.2011					0.000000	12.9
15.10.2011					3.227763	13.1
16.10.2011					0.000000	13.4
17.10.2011					0.000000	13.9
18.10.2011					6.454707	3.4
19.10.2011					3.227217	2.2
20.10.2011					3.227081	2.0
21.10.2011					4.840416	3.9
22.10.2011					1.613404	2.7
23.10.2011					0.000000	2.7
24.10.2011					1.613267	5.2
25.10.2011					0.000000	7.2
26.10.2011					1.613131	8.4
27.10.2011					0.000000	4.2
28.10.2011					3.225989	-0.1
29.10.2011					1.612926	-1.7
30.10.2011					0.000000	-5.4
31.10.2011					1.612790	-3.7
01.11.2011					3.225443	-2.2
02.11.2011					3.225307	-4.4
03.11.2011					3.225171	0.7

Data	Chelyabinsk		Yekaterinburg		Barnaul	
	RI rates	Temperature	RI rates	Temperature	RI rates	Temperature
04.11.2011					8.062586	1.9
05.11.2011					0.000000	3.2
06.11.2011					0.000000	1.8
07.11.2011					0.000000	-5.7
08.11.2011					3.224489	-6.1
09.11.2011					1.612176	-12.3
10.11.2011					1.612108	-11.5
11.11.2011					4.836120	-13.1
12.11.2011					4.835916	-6.3
13.11.2011					4.835712	-4.2
14.11.2011					3.223672	-11.9
15.11.2011					3.223535	-10.3
16.11.2011					3.223399	-9.5
17.11.2011					3.223263	-9.7
18.11.2011					1.611563	-7.5
19.11.2011					8.057476	-7.9
20.11.2011					4.834282	-11.4
21.11.2011					1.611359	-10.5
22.11.2011					1.611291	-6.3
23.11.2011					1.611223	-9.8
24.11.2011					4.833465	-13.8
25.11.2011					3.222174	-15.8
26.11.2011					0.000000	-14.3
27.11.2011					6.443803	-15.8
28.11.2011					3.221766	-15.1
29.11.2011					4.832444	-13.3
30.11.2011					1.610747	-13.0
01.12.2011					1.610679	-11.7
02.12.2011					4.831832	-7.3
03.12.2011					1.610543	-8.4
04.12.2011					3.220949	-9.8
05.12.2011					3.220813	-10.7
06.12.2011					3.220677	-8.2
07.12.2011					6.441083	-8.2
08.12.2011					3.220406	-11.8
09.12.2011					1.610135	-12.9
10.12.2011					3.220134	-13.1
11.12.2011					3.219998	-18.2
12.12.2011					4.829793	-17.2
13.12.2011					1.609863	-15.1
14.12.2011					1.609795	-11.7

Data	Chelyabinsk		Yekaterinburg		Barnaul	
	RI rates	Temperature	RI rates	Temperature	RI rates	Temperature
15.12.2011					16.097271	-12.2
16.12.2011					9.657955	-17.0
17.12.2011					3.219183	-20.1
18.12.2011					3.219047	-21.4
19.12.2011					4.828366	-17.3
20.12.2011					3.218775	-15.9
21.12.2011					1.609320	-18.0
22.12.2011					6.437007	-14.2
23.12.2011					9.655103	-12.2
24.12.2011					3.218232	-14.8
25.12.2011					6.436192	-15.6
26.12.2011					6.435921	-11.3
27.12.2011					1.608912	-7.1
28.12.2011					0.000000	-10.2
29.12.2011					0.000000	-5.7
30.12.2011					1.608709	-3.8
31.12.2011					0.000000	-7.3
